# Adsorption of Surfactants and Polymers to Biomimetic Hair Model Surfaces

**DOI:** 10.1021/acs.langmuir.5c06252

**Published:** 2026-03-06

**Authors:** Serena Cozzolino, Philipp Gutfreund, Inger Odnevall, Raam Ibrahim, Alexei Vorobiev, Rebecca J. L. Welbourn, Francesca Zuttion, Andrew Greaves, Gustavo S. Luengo, Mark W. Rutland

**Affiliations:** † Division of Surface and Corrosion Science, School of Engineering Sciences in Chemistry, Biotechnology and Health, 7655 KTH Royal Institute of Technology, SE-100 44 Stockholm, Sweden; ‡ 56053 Institut Laue-Langevin, 71 avenue des Martyrs, CS 20156, 38042 Grenoble cedex 9, France; § Department of Physics and Astronomy, Materials Physics, Uppsala University, SE-751 20 Uppsala, Sweden; ∥ ISIS Pulsed Neutron and Muon Facility, Rutherford Appleton Laboratory, Didcot, Oxfordshire OX11 0QX, U.K.; ⊥ L’Oréal Research and Innovation, 1 avenue Eugène Schueller, 93600 Aulnay-sous-Bois, France; # Bioeconomy and Health Department, Materials and Surface Design, RISE Research Institutes of Sweden, SE-114 28 Stockholm, Sweden; ∇ School of Chemistry, University of New South Wales, Sydney, NSW 2052, Australia; ○ Laboratoire de Tribologie et Dynamique des Systèmes, École Centrale de Lyon, 69134 Ecully CEDEX, France

## Abstract

Improving the sustainability of cosmetic products while maintaining a good performance requires a deeper understanding on the way that new eco-respectful ingredients interact with hair or skin. In the case of shampoos, the surface science is dominated by the diverse changes on the hair fiber due to both chemical and physical damages that particularly affect physicochemical properties such as hydrophobicity. A native, undamaged fiber is covered with a monolayer of lipids, mainly 18-methyleicosanoic acid (18-MEA), while a highly damaged hair surface, having completely lost the protective lipids, is hydrophilic and negatively charged. Intermediate states exist, where there is a partial loss of 18-MEA (“partially damaged hair”). Here, four model surfaces have been produced, to mimic different types of hair surfaces. Their interaction with selected surfactants and polyelectrolytes (natural and synthetic) has been studied by neutron reflectometry (NR). NR can reveal hierarchical adsorption from mixtures thanks to the scattering contrast between deuterated and hydrogenous molecules. Atomic force microscopy (AFM) measurements complement the study by adding information about the in-plane structure of adsorbed species. The presence of the methyl branch of 18-MEA is found to affect the interaction of the surface with adsorbates. For surfactant/polyelectrolyte mixtures, for example, the adsorption of polymer is enhanced. Of particular interest are the results on the partially damaged hair model, as it manifests patches of hydrophobic and hydrophilic moieties; it is possible to separately observe the different adsorption behaviors to the different sites in a single experiment.

## Graphical abstract

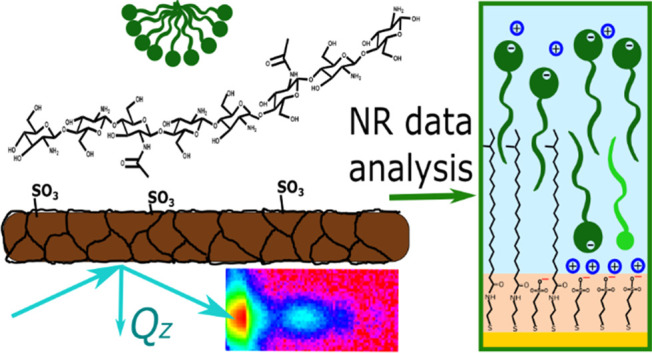

## 1 Introduction

Human hair is normally covered by a monolayer of covalently bound lipids that creates a hydrophobic surface.^1,2^ Lipid composition and density vary even along the same fiber, as a response to weathering or chemical treatments, that can remove part of the hydrophobic layer.^3–6^ Lipid removal implies breaking the thioester bond with cysteine residues from the underlying proteins; the cysteine thiol is then oxidized to a sulfonate moiety, which makes the damaged hair surface hydrophilic and negatively charged.^4,7,8^ The most abundant lipid is normally 18-methyleicosanoic acid (18-MEA),^1,9^ a fatty acid with an antepenultimate methyl branch that has a different molecular cross-section and packing compared to straight-chain lipids.^10^ It is thus responsible for layer fluidity, besides having possible bacteriostatic properties.^11–13^ The specific structure of hair lipids can affect the interaction of the fiber with hair-care products. A lot of research is currently being carried out on this topic, as a deeper understanding of the interaction properties of hair is essential to design improved and eco-friendly formulations. Traditional formulations are often based on petrochemical ingredients which have been optimized over many decades. In the last 20 years or so, massive investment has been made to replace such ingredients with performant, eco-friendly versions. Even so, there is still a need to mechanistically understand how these new ingredients work so performance is not compromised.^14–16^ Part of the research in the field is implemented *in silico*: the interaction of hair with model actives is simulated to quickly screen potential candidates for more sustainable formulations.^3,17–19^ Although the hair surface can be represented in its simplest form as hydrophobic and hydrophilic patches, very sophisticated models are now available, reproducing the outermost part of the protein core and grafted 18-MEA molecules at varying density.^20–22^ Experimental research, in contrast, is limited to mimicry of the surface charge and hydrophobicity,^6,23,24^ at least if not performed directly on tresses (e.g.,^25^). Very few examples are found in the literature of more complex hair-mimetics,^26,27^ and none yet that specifically consider 18-MEA to better understand the role of the methyl branch in adsorption of hair-care components.

In this paper, the production of such biomimetic models, shown in Figure 1, is presented. A thiol derivative of 18-MEA is used to easily obtain the hair model by functionalizing a gold-coated surface thanks to its known affinity to sulfur.^28–31^ A similar model obtained by use of the straight chain analogue eicosanoic acid (EA) helps in defining the contribution of the methyl branch to the interaction properties of hair, while the effect of partial damage of the lipid layer is addressed by mixing 18-MEA with a sulfonate-terminated thiol.

**Figure 1:**
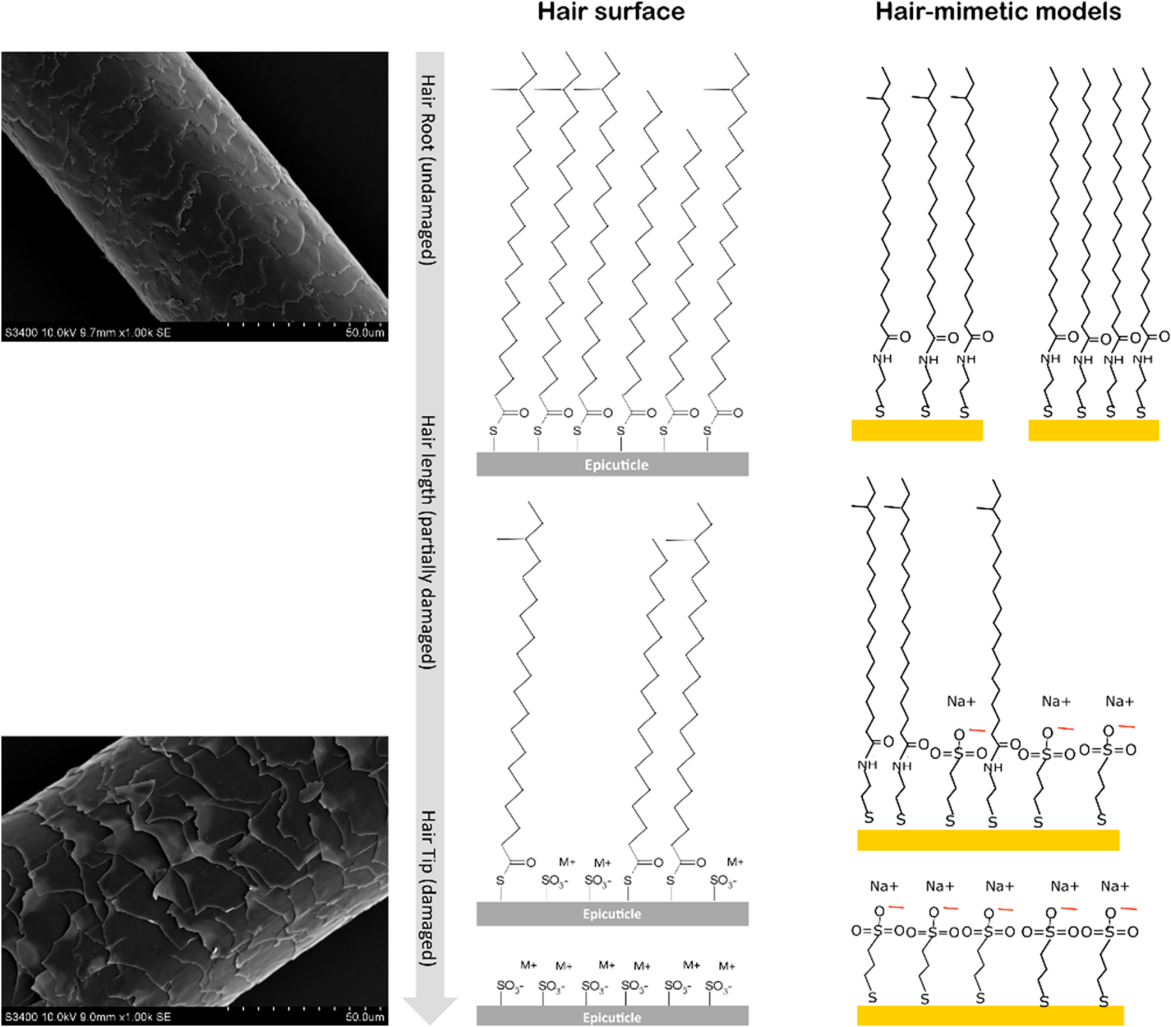
On the left, SEM (Scanning Electron Microscopy) images of native (top) and damaged (bottom) hair fiber. The structures in the middle show the best models to describe the hair surface, while those on the right represent the models produced in this paper.

Adsorption is studied by Neutron Reflectometry (NR), a technique capable of resolving a layered structure, in the direction normal to the surface, at a subnanometre resolution, while adding information on chemical composition thanks to the hydrogen/deuterium neutron scattering contrast.^32^ However, the description obtained by NR is averaged in-plane, so experiments are complemented by Atomic Force Microscopy (AFM) to add topographical information about the adsorbed structures. The adsorption behavior of model actives is evaluated by comparing an anionic (sodium dodecyl sulfate, SDS) and a cationic (cetyltrimethylammonium chloride, CTAC) surfactant at different concentrations and in mixture with a natural polysaccharide (chitosan). Despite not being the most common surfactant found in hair-care formulations (sodium laureth sulfate (SLES) being the preferred one), SDS was chosen because it is easier to obtain in deuterated form, has an unambiguous atomic composition (while the ethoxy spacer in SLES has a variable number of repeats) and has a good environmental profile. The first two factors are necessary features to fully harness the potential of NR in determining the hierarchy of adsorption from complex solutions. Note that we deliberately limit the study to model systems; real formulations have many more components and the composition of the thiol layers is simplified compared to the range of chain lengths found on real hair. Nonetheless, we would like to emphasize that the hair models presented below are, to the best of our knowledge, the first ones to specifically include the peculiar methyl branch of 18-MEA. Compared to our previous study,^26^ we here use a longer hydrocarbon chain to increase mimicry of the 18-MEA layer packing. Finally, poly­(diallyl dimethylammonium chloride) (pDADMAC) is studied as an example of a common synthetic polymer, for which chitosan constitutes an interesting and more sustainable alternative.^33^ In hair-care, a plethora of cationic polymers is commonly used, and some are derived from natural polymers, e.g., polyquaternium-10 (from cellulose) and guar hydroxypropyltrimonium chloride (from guar gum). However, they have been chemically modified often using nonbiobased reagents.^34^ On the contrary, chitosan does not undergo such chemical transformations and it is prepared from natural sources of chitin.

## 2 Materials and Methods

### 2.1 Chemicals and Production of Hair-Mimetic Surfaces

Thiol derivatives of 18-MEA and EA (both with purity >95%) were provided by L’Oréal. Their structures are shown in Figure 2. They were used to produce two types of “healthy hair” model surfaces and study the effect of the methyl branch on the interaction with cosmetic ingredients. Please note that an amide moiety is present in the structure rather than the thioester group that is found on the hair surface. This was due to the synthetic route employed, aimed at maintaining the carboxylic carbon to better mimic the molecular cross Sections. Instead, for the “damaged hair” models, sodium 3-mercapto-1-propanesulfonate (PS, 90% purity, Sigma-Aldrich) was used, pure or mixed with 18-MEA thiol, to mimic the sulfonate moiety that forms when 18-MEA is removed from the fiber, as already done previously.^26,27,33^ It is worth mentioning that the ethyl spacer visible in the structures of 18-MEA and EA thiols allows the amide group to be at a similar height as the sulfonate from PS. The partially damaged hair model thus reproduces the mismatch in height that is present on a partially damaged hair fiber.

**Figure 2:**
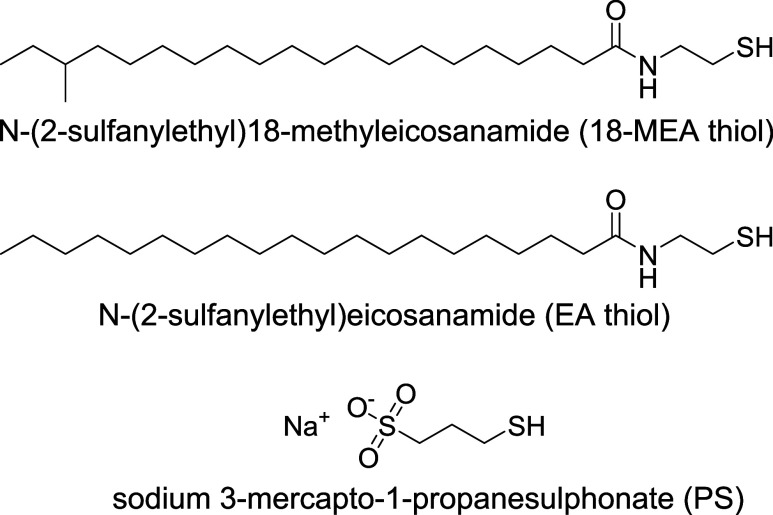
Structure of the molecules used to produce the hair-mimetic surfaces: thiol derivatives of 18-MEA and EA (“healthy hair” models), and sodium 3-mercapto-1-propanesulfonate (PS, “damaged hair” model).

The hair-mimetic surfaces were obtained by immersion of gold coated substrates in a 1 mM solution of thiol(s) in absolute ethanol (Sigma-Aldrich), following established protocols.^28^ The substrates functionalized with pure thiols (18-MEA thiol, EA thiol or PS) were incubated for 24 h. It is known, in fact, that even though long-chain thiols quickly adsorb on the gold surface, the self-assembled monolayer (SAM) can rearrange for days,^28^ while in the case of PS it has been reported that its SAM has a low coverage,^35^ so a 24 h incubation was chosen to allow enough time to obtain a SAM of good quality. In the case of the fourth system, i. e., the mixed PS:18-MEA thiol surface, the normal overnight incubation was used, as a compromise between having a good SAM and avoiding long immersion periods that, for mixed structures, can result in one thiol fully replacing the other one on the surface, depending on their relative affinities.^28^ Two ratios (in solution) were used: 50:50 and 80:20 PS:18-MEA thiol (the choice of the second ratio is explained in Section S3 the Electronic Supporting Information, ESI).

Hydrogenous and deuterated (-*d*_25_) sodium dodecyl sulfate (h- and d25-SDS, > 99% and >98% purity, respectively), poly­(diallyl dimethylammonium chloride) (pDADMAC, 20% w/w solution), sodium chloride and deuterated water were from Sigma-Aldrich, while deuterated (-*d*_42_) hexadecyl trimethylammonium chloride (d42-CTAC, 98% purity) was from CDN Isotopes. Chitosan of two average molecular weights was used: a 3 kDa molecule (deacetylation >97%, purity 80%), referred to as oligomer, and a 27 kDa molecule (deacetylation >95%, pure), referred to as polymer, both of fungal origin. Structures of the selected compounds are in Figure 3.

**Figure 3:**
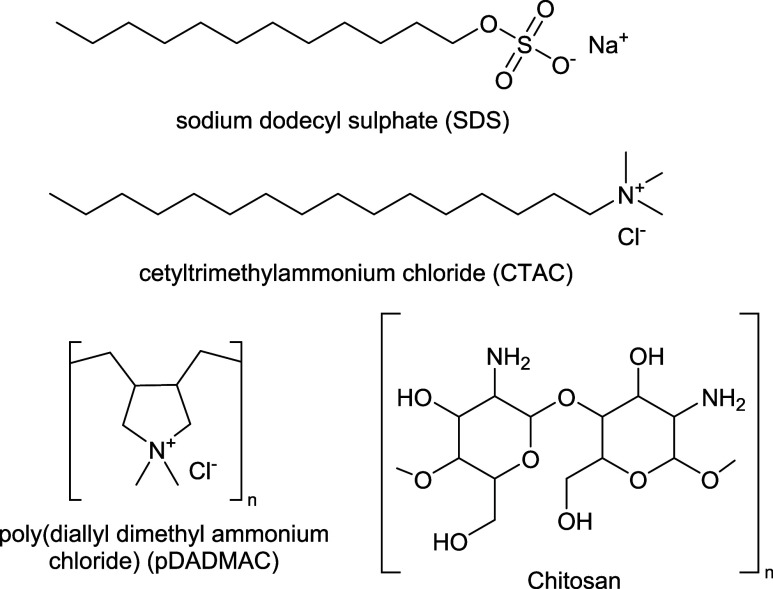
Structure of the surfactants (SDS, CTAC) and polyelectrolytes (pDADMAC, chitosan) used in the adsorption experiments.

Solutions were prepared by dissolving the appropriate amount of surfactant and/or polyelectrolyte in either water (Milli-Q quality, Millipore) or a mixture of Milli-Q and deuterated water. Polyelectrolytes had a concentration of 100 ppm by mass, while surfactant concentration was defined in terms of their critical micellar concentration (cmc). The ionic strength was kept constant by addition of 100 mM NaCl. In these conditions, the cmc of SDS is 1.5 mM,^36^ while that of CTAC is 0.065 mM.^37^ The pH of the solutions was ≈6 in every case except for chitosan polymer, which required acetic acid (Sigma-Aldrich) to dissolve, and the final solution had a pH of 4. While this pH is low, it does not impact the relevance since many formulations employ lower pH.^38^

### 2.2 Neutron Reflectometry (NR)

As mentioned in the Introduction, NR can give information about any layered structure at the interface, in terms of thickness, roughness and composition of the layers. The latter is related to the Scattering Length Density (SLD) parameters, which depend on the isotopic composition of a molecule; the SLD of an adsorbed layer will then be a mix of any adsorbed components plus water due to hydration or patchiness (a detailed description of the technique can be found in the literature^39–43^ ). The experiments were conducted at the ISIS Neutron and Muon Source (Oxfordshire, U.K.), on the instrument INTER,^44^ and at ILL (Grenoble, France), on either FIGARO^45^ or SuperADAM.^46^ INTER and FIGARO are Time-of-Flight (ToF) reflectometers: the specularly reflected intensity was recorded as a function of neutron wavelength λ, at two incident angles θ to cover a Q-range up to 0.33 Å^–1^, Q being the momentum transfer vector: *Q* = (4π*/λ*)*sinθ.* On INTER, θ_1_ = 0.7° and θ_2_ = 2.3°, while on FIGARO θ_1_ = 0.8° and θ_2_ = 3.2°. SuperADAM, instead, is a monochromatic instrument, with λ = 5.21 Å, and the reflected intensity was recorded as a function of θ to explore in this case a Q-range up to 0.2 Å^–1^. The experiments were conducted at a constant temperature of 22 °C. The substrates were silicon blocks with a surface of 50*50 mm^2^, coated with 50 Å titanium and 200 Å gold. They were characterized by X-ray reflectometry before functionalization. Thiolated blocks were then mounted in a solid/liquid cell^26^ and the thiol layers were first measured in solvent, i.e., gold contrast-matched water (GCMW) containing 100 mM NaCl. GCMW (a mixture of 74% D_2_O and 26% H_2_O, SLD = 4.6 × 10^–6^Å^–2^) was chosen to have the best contrast to both hydrogenous and deuterated species in solution.^26^ On the ToF instruments, the beam footprint was chosen to measure only from within the solid–liquid interface region, while SuperADAM has a variable footprint and sample overillumination was corrected for during data reduction.

Three adsorption sequences were performed. Sequence NR1 and NR2 were both run on 18-MEA thiol, EA thiol and 50:50 PS:18-MEA thiol. Sequence NR2 was used also for a pure PS system, while a similar sequence to NR1 has already been published regarding pure PS.^26^ The cells, one for each model surface, were mounted on the sample stage and connected to a multiposition valve, in turn connected to syringe pumps to inject the desired solutions sequentially. As results on the mixed thiol surface suggested that the ratio of thiols on the surface was closer to pure 18-MEA than to the solution ratio, a new sample was prepared incubating a gold-coated block in a solution containing 80:20 PS:18-MEA thiol. Sequence NR3 was then run on the 80:20 mixed thiol sample, including the main points of the two previous experiments, with some variation due to time constraints. The exact steps for each sequence are listed in Table 1 and 2.

**Table 1:** Adsorption Sequences NR1, Run on 18-MEA Thiol, EA Thiol and 50:50 PS:18-MEA Thiol Surfaces, and NR2, Run on PS and on New Samples of 18-MEA Thiol, EA Thiol and 50:50 PS:18-MEA Thiol^a^

NR1		NR2	
Adsorbing species	Concentration	Adsorbing species	Concentration
d25-SDS	0.1 cmc (0.15 mM)	d42-CTAC	0.1 cmc (0.007 mM)
	0.5 cmc (0.75 mM)		0.5 cmc (0.03 mM)
	2 cmc (3 mM)		2 cmc (0.13 mM)
	20 cmc (30 mM)		20 cmc (1.3 mM)
d25-SDS/chitosan oligomer	20 cmc/100 ppm	d42-CTAC/chitosan oligomer	20 cmc/100 ppm
			62.5 cmc/12.5 ppm
Rinse		Rinse	
d25-SDS	20 cmc (30 mM)	Chitosan oligomer	100 ppm
Rinse		Rinse	
		Chitosan polymer	100 ppm
		d25-SDS	20 cmc (30 mM)
		Rinse	
		pDADMAC	100 ppm
		Rinse	
		h-SDS	0.5 cmc (0.75 mM)
			20 cmc (30 mM)
		Rinse	

a Surfactant concentrations are in terms of their critical micellar concentration (cmc) and corresponding molarity. Polyelectrolyte concentrations are given as ppm by mass (see Section 2.1 for more details).

**Table 2:** Adsorption Sequences NR3, Run on the 80:20 PS:18-MEA Thiol Surface^a^

NR3	
Adsorbing species	Concentration
d25-SDS	2 cmc (3 mM)
	20 cmc (30 mM)
Rinse	
Chitosan oligomer	100 ppm
Rinse	
d25-SDS	2 cmc (3 mM)
d25-SDS/chitosan oligomer	20 cmc/100 ppm
Rinse	
d42-CTAC	0.1 cmc (0.007 mM)
	0.5 cmc (0.03 mM)
	2 cmc (0.13 mM)
	20 cmc (1.3 mM)
d42-CTAC/chitosan oligomer	20 cmc/100 ppm
	62.5 cmc/12.5 ppm
Rinse	
Chitosan polymer	100 ppm
h-SDS	20 cmc (30 mM)
Rinse	
pDADMAC	100 ppm
Rinse	

a Surfactant concentrations are in terms of their critical micellar concentration (cmc) and corresponding molarity. Polyelectrolyte concentrations are given as ppm by mass (see Section 2.1 for more details).

The surfactant/polyelectrolyte ratio of 20 cmc/100 ppm was chosen for the SDS/chitosan complex to reproduce the weight percentages of a shampoo, although diluted 10 times^3,14,26^ (please note that, while the concentration of adsorbing species is 10-fold lower than in shampoos, the concentration of NaCl is in the range of the exact one,^38^ so that ionic strength and surfactant cmc are similar to those in actual formulations). In the case of the CTAC/chitosan oligomer mixtures, two ratios were chosen to compare the cationic surfactant with the anionic SDS: the first one is the same in terms of cmc of surfactant, but as the cmc of CTAC is significantly lower than that of SDS, the second mixture (62.5 cmc/12.5 ppm) was added to have the same ratio in terms of molar concentration (having a chitosan concentration of 100 ppm, it would imply a CTAC concentration of 500 cmc—this had to be diluted three times to obtain a transparent solution).

The other steps were designed to investigate the effect of the order of injection of the separate components (SDS after exposure of the surface to chitosan or vice versa) and the influence of the molecular weight of chitosan (oligomer vs polymer). Finally, pDADMAC was used to compare adsorption of chitosan with that of a synthetic polymer, and h-SDS was added to collect data with different scattering contrast.

NR data were reduced using standard methods for each instrument: the Mantid Workbench^47^ (INTER data), COSMOS^48^ (FIGARO data—in this case reduction included a step for background subtraction), or pySAred^49^ (SuperADAM data). Data analysis was performed using *refnx* (v. 0.1.32),^50^ applying a slab model that was first fitted using a differential evolution algorithm and then refined after running a Markov-Chain Monte Carlo analysis,^51^ as described previously.^26^ For the fitting, SLD values of the components were calculated based on the atomic composition and density of the dry substance, using the NIST calculator.^52^ Values are reported in Table 3.

**Table 3:** Values of Density (ρ) and SLD for the Dry Substances Used in the NR Experiments

	ρ (g mL^–1^)	SLD (10^–6^ Å^–2^)
18-MEA thiol	0.85	–0.015
EA thiol	0.85	–0.004
PS	1.4	1.1
d25-SDS	1.1	6.2
d42-CTAC	1.0	7.7
h-SDS	1.0	0.28
Chitosan	1.34 ^53^	3.1 ^54^
pDADMAC	1.04	3.1

### 2.3 Atomic Force Microscopy (AFM)

AFM measurements were performed on selected steps to add information on the in-plane structure of the adsorbed layer. Template-stripped gold surfaces (Platypus technologies) were used as substrates in this case. Samples were imaged on a Dimension Icon (Bruker), in PeakForce QNM (Quantitative Nanomechanics) mode in liquid (tips: ScanAsyst Air, ν = 70 kHz, *k* = 0.4 N/m, Bruker). 18-MEA thiol, PS and 50:50 PS:18-MEA thiol samples were produced in duplicate, and 2–3 topographical images were recorded at three different spots on each surface (scan size 5 μm, 256 pixels). Images were acquired in water, then in 100 ppm chitosan oligomer, 2 cmc h-SDS, and in a mixture of 20 cmc h-SDS and 100 ppm chitosan oligomer. All the solutions contained 100 mM NaCl, as in NR experiments, and were filtered just before use with a 0.2 μm syringe filter. Data analysis was carried out using the software Gwyddion.^55^ For data visualization, the software MountainsSPIP (Digital Surf, Besançon) was used; in the case of the images presented in Figure 5, a correction was applied to level out holes due to the gold substrate, for better understanding.

## 3 Results and Discussion

### 3.1 Structure of the Hair-Mimetic Systems

The thiol derivatives of 18-MEA and EA employed to produce the healthy hair models showed a very different self-assembled structure, compared to conventional thiols.^26,28^ Structures thicker than a monolayer were observed, compatible with the model described by Wang *et al.*,^21^ i.e., “free” chains intercalated in a monolayer of molecules bound to the surface, probably due to the presence of the amide group (see Figure 2) that may prevent a close packing of the hydrophobic chains. Addition of surfactants removed these unbound chains, eventually leaving a conventional thiol monolayer. The aim of this paper is to address the interaction with surfactant/polyelectrolyte systems, so the thiol characterization is confined to Section S1 in the ESI. Concerning the damaged hair models, Figure 4 shows the depth profiles of the fully damaged hair model (pure PS) and the partly damaged hair models obtained by mixing PS and 18-MEA thiol in different proportions in the bulk (the results here shown are limited to the SLD profiles corresponding to the biomimetic surface and adsorbed layer(s)—the full SLD profiles and the corresponding NR curves can be found in Section S5 in the ESI).

**Figure 4:**
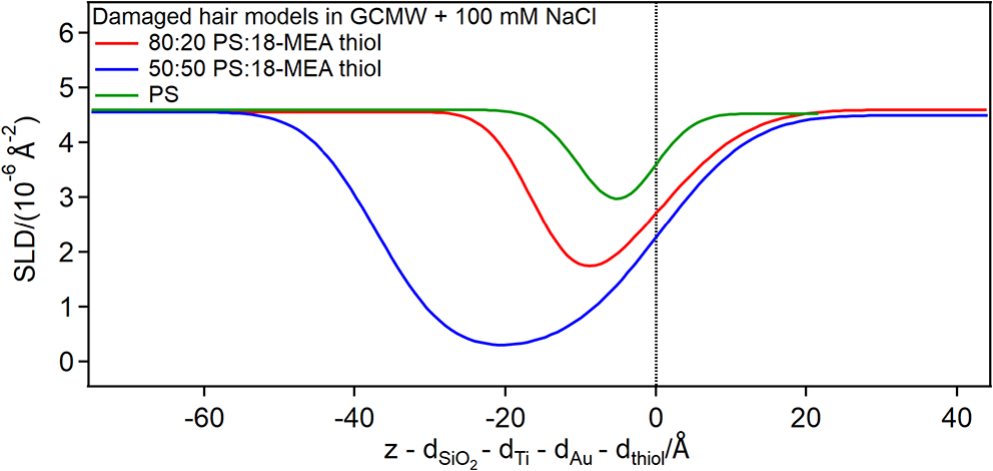
Depth profiles of the three damaged hair model surfaces (zoom in the thiol region—the full SLD profile can be found in Section S5 in the ESI). The zero on the *x*-axis is at the thiol/solution interface, so that the gold-coated substrate and the thiol layers are at negative *x*-values, while the solution is at positive ones.

The three structures vary in thickness from 6 Å (PS—in line with the molecular dimensions and previous observations^26^) to 15 Å (80:20 mixture—in agreement with literature values for a similar system^20^) to 38 Å (50:50 mixture). The latter thickness is closer to the bilayer values for pure 18-MEA thiol (whose extended molecular length is close to 30 Å; see Section S1 and Table S6 in the ESI), and the fitted SLD parameter suggests that the layer contains only 19% PS. AFM data (examples in Figure 5) show the presence of patchy regions (Figure 5a), whose height is compatible with the difference in chain length of the two thiols, with other regions on the same surface being more homogeneous (Figure 5b), thus the mixed thiol systems show characteristics of both patches and mixing behavior.

**Figure 5:**
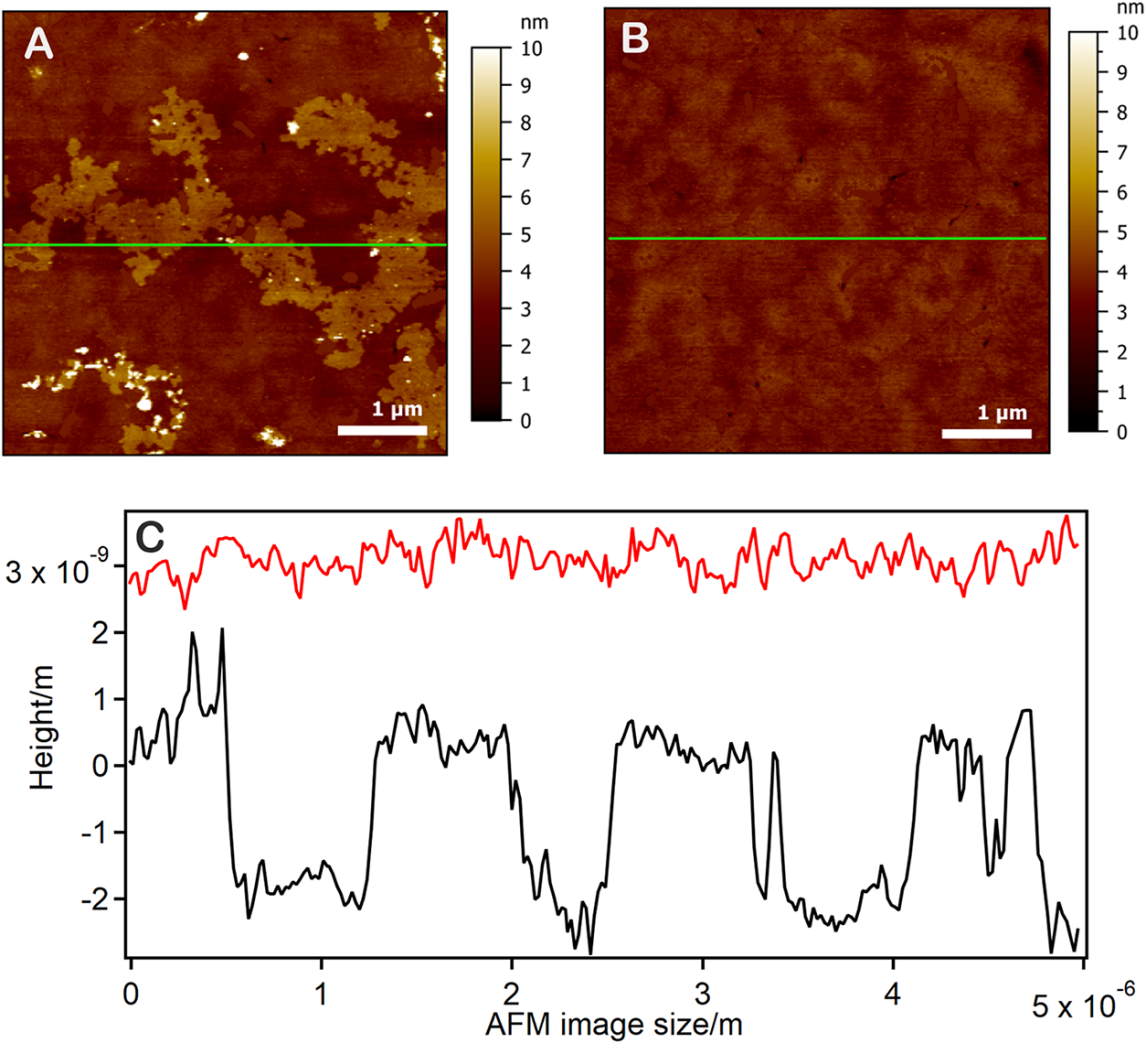
Example of AFM images (a, b) and corresponding profiles (c) (extracted along the green lines indicated in the AFM images) relative to a 50:50 PS:18-MEA thiol sample in water plus 100 mM NaCl. (a) Shows a region with patches of different heights (black profile in c), (b) a more homogeneous region of the same surface (red profile in c).

Independent X-ray Photoelectron Spectroscopy (XPS) measurements were run, in parallel to the NR experiments, on a mixture of PS and the long-chain alkylthiol ODT (octadecanethiol, without amide linker). They indicated that, to obtain 60% sulfonate moieties on the surface (considering the average value for the ratio of oxidized to total sulfur^56^), ca. 80% PS is required in the thiolating solution (XPS data are in Section S3 in the ESI). We ascribe this to a more favorable adsorption of the longer chain species, due to self-assembly interactions. Note that no such XPS quantification could be performed on the 18-MEA and EA thiols due to the anomalous (bilayer) adsorption behavior discussed above. Based on the values in Table 3, the fitted SLD for the 80:20 (solution) mixture (see Table S9 in the ESI) indicates in fact the presence of 64% PS on the surface, assuming no solvent in the layer.

### 3.2 Adsorption of Surfactants on Damaged Hair Models

The interaction of SDS with a fully damaged model surface has been described in a previous publication:^26^ it was observed that, unexpectedly, SDS adsorbs, from a 20 cmc surfactant solution, as a bilayer enriched in dodecanol (SDS is consciously used without purification of the commercial sample, to be as close as possible to a real formulation, so the presence of dodecanol is expected^57^). Adsorption occurs due to hydrophobic interactions, which are of entropic origin. The electrostatic interactions are screened by the presence of sodium ions, and reduced in magnitude by the presence of the uncharged alcohols. On the partially damaged hair model, i.e., the 80:20 PS:18-MEA thiol sample, the interaction occurs in two different ways, reflecting the presence of two thiol regions, as shown in Figures 6 and 7.

**Figure 6:**
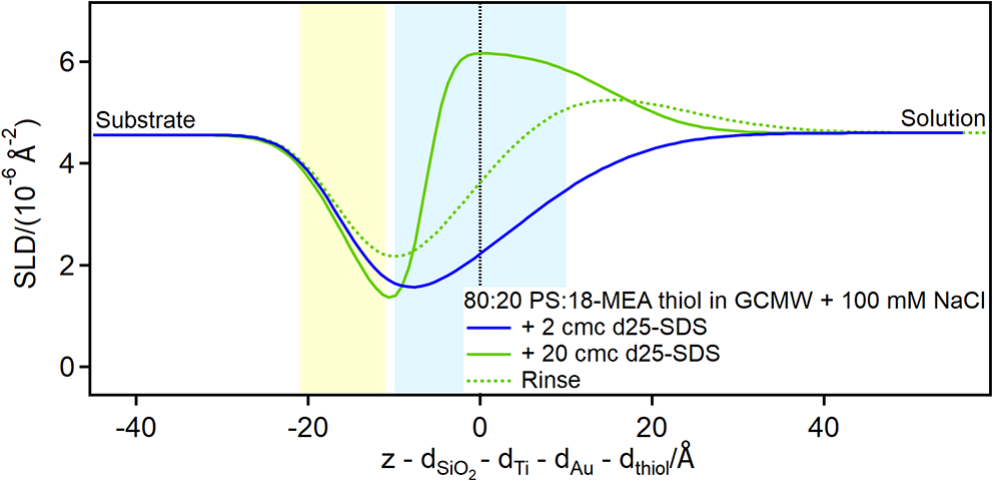
Depth profiles of the partially damaged hair model in the presence of d25-SDS at the concentrations of 2 and 20 cmc, and following rinse. The yellow and blue panels represent the Au/thiol and the thiol/solution interfaces, respectively, with associated roughness. The zero value on the *x*-axis corresponds to the thiol/solution interface in pure solvent, so that the gold-coated substrate appears at negative *x*-values and adsorbed layers at positive ones.

**Figure 7:**
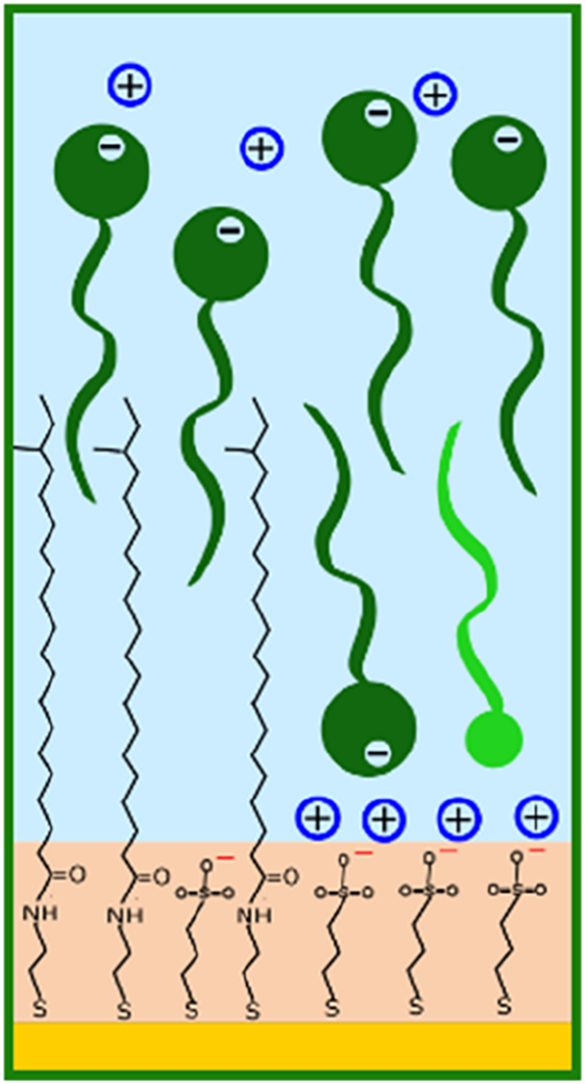
Schematic drawing showing the two possible mechanisms for adsorption of SDS (dark green charged molecules) on the two patches of the partially damaged hair model. The light green uncharged molecule represents a dodecanol residue. Blue circles are sodium ions.

In this case, the depth profile shows no significant variation in the presence of 2 cmc d25-SDS compared to the surface in pure solvent, but at 20 cmc an adsorption peak is visible, clearly shifted in the region previously assigned to thiols. This can be interpreted as an intercalation of the dodecyl chains (highest SLD component) in between the protruding 18-MEA molecules and adsorption of SDS/dodecanol species to PS patches, as illustrated in Figure 7.

The introduction of a cationic surfactant, instead, creates a more complex scenario. Figure 8 shows the SLD profiles of the partially damaged hair model in the presence of increasing concentrations of d42-CTAC (it is worth reminding here that this was part of the sequence NR3: d42-CTAC was introduced on a surface that had already been exposed to other adsorbing species).

**Figure 8:**
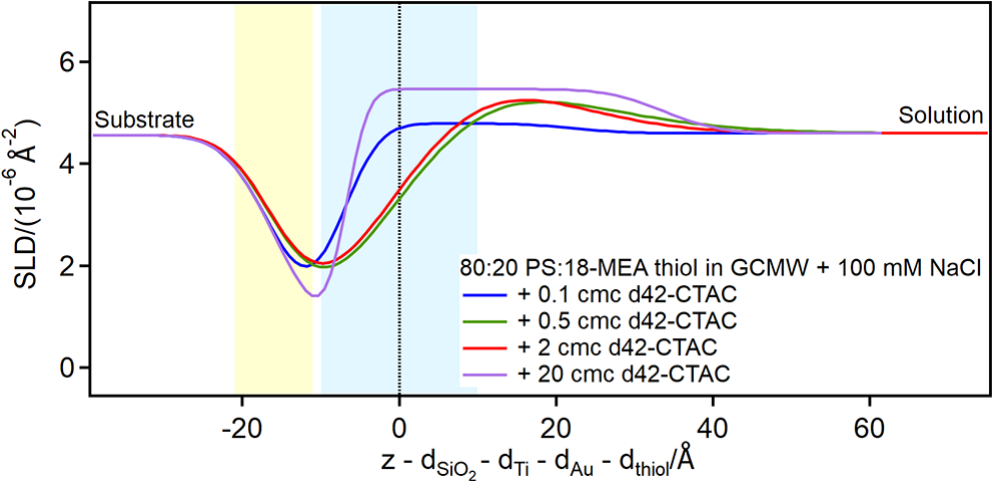
Depth profiles of the partially damaged hair model in the presence of d42-CTAC at the concentrations indicated in the graph. The yellow and blue panels represent the Au/thiol and the thiol/solution interfaces, respectively, with associated roughness. The zero value on the *x*-axis corresponds to the thiol/solution interface in pure solvent.

At 0.1 cmc d42-CTAC, the adsorbed peak is not significantly different from that of the residual SDS (from the previous steps), but at intermediate concentrations (0.5 and 2 cmc) the adsorbed layer shifts to higher *z* values, i.e., toward the bulk. At 20 cmc d42-CTAC, finally, the peak position again overlaps with the thiol region, but extends also in solution. The total thickness of the adsorbed layer here is 40 Å, compatible with a bilayer of CTA^+^ ions^58^ (the reported length for this cation is 2.2 nm, meaning that molecules in the bilayer might be slightly tilted, however the difference is within the error of the layer thickness—see Table S9 in the ESI). An explanation for these profiles is that the cationic surfactant interacts with the adsorbed SDS residue and removes it, so when the more concentrated solution is injected, it adsorbs on a (mostly) clean surface (strictly the unlikely possibility of a persistent contribution from previously adsorbed species cannot be completely ruled out, in view of only a single contrast). In this scenario, SLD and thickness values suggest that the surfactant may be adsorbing in two conformations, similar to SDS adsorption in Figure 7: on the one hand it intercalates in between 18-MEA chains, exposing the headgroups to the bulk; on the other hand, it adsorbs as a bilayer on the hydrophilic patches, with headgroups interacting with the sulfonate moieties and with the bulk. (Note that d42-CTAC is also shown to adsorb as a bilayer on the fully hydrophilic PS surface, as discussed in the ensuing Section 3.3 comparing adsorption in mixed systems).

### 3.3 Adsorption of Surfactants, Chitosan Oligomer and Their Mixtures

Adsorption of chitosan oligomer on the different model surfaces was studied in the presence of both SDS and CTAC, comparing the effect of a premixed surfactant/polyelectrolyte solution with the sequential adsorption of surfactant and polyelectrolyte from separate solutions. This is particularly interesting in the case of the premixed SDS/chitosan solution, since the two components are known to aggregate and this might change their interaction with the surface. In the literature, there are numerous examples of the physicochemical characterization of such mixtures, exploring different SDS/chitosan ratios and polymer molecular weights.^59–64^ It was reported that, at increasing SDS concentration, surface charge decreases faster for a 4 kDa than for a 48 kDa chitosan solution, from which the authors inferred that SDS preferably binds to lower molecular weight chitosan.^60^ The aggregates can be as large as 2000 nm, as SDS-saturated chitosan molecules can associate via hydrogen bonds to form microparticles.^60^ However, the cited paper deals with SDS/chitosan mixtures at ratios that result mostly in turbid samples (0.1 wt % chitosan and SDS concentrations below 1 mM—according to the diagram shown in,^59^ this ratio is close to the phase separation regime). The solutions prepared in the present work, instead, are clear, being chitosan more diluted and SDS in large excess. In fact, for a chitosan concentration of 0.01 wt % (corresponding to 100 ppm, as used here), the required SDS amount to obtain a charge inversion was reported to be 3 mM,^62^ i.e., it is ten times lower than the concentration used in our experiments. This means that a large number of free surfactant micelles is present in solution.

In our adsorption studies, the two hydrophobic surfaces show a different interaction with the d25-SDS/oligomer complex. On the unbranched EA thiol, the peak in the presence of the complex is indistinguishable from that of pure d25-SDS, and its thickness (see Table S7 in the ESI) is compatible with standing-up dodecyl sulfate molecules.^65^ Upon rinsing, the intensity of the peak decreases slightly, but it is restored with the subsequent injection of pure surfactant, decreasing again when rinsing (Figure S4 in ESI). This means that SDS saturates the surface preventing interaction with chitosan (as the peak relative to the adsorbed layer is always positive, we can only confidently detect the presence of deuterated species, i.e., SDS), and that SDS adsorption is partially irreversible, at least with respect to the rinse routine employed here. Instead, on the 18-MEA thiol, injection of the complex causes an increase in the intensity of the adsorption peak (see Figure 9—please note that the 18-MEA thiol data are presented as depth profiles from which the “clean” thiol layer has been subtracted, as explained more in detail in Section S1 in the ESI). In both cases (d25-SDS alone or in mixture with chitosan) the thickness of the adsorbed layer, 7 Å, is clearly lower than the molecular length. The simplest explanation is that SDS adsorbs in a tilted orientation. This scenario agrees with the results by Coscia *et al.*,^17^ who investigated adsorption on 18-MEA by molecular dynamics simulations. However, a partial intercalation in between 18-MEA chains is also possible. The different behavior compared to the EA thiol surface presented above might be due to the different packing of the thiol chains, that drives different interactions with the dodecyl tails.

**Figure 9:**
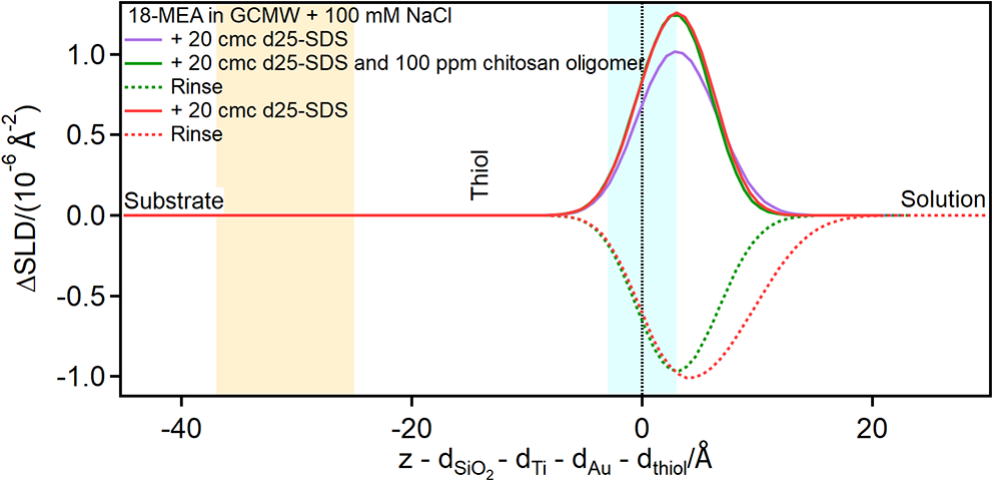
Subtracted depth profiles of a healthy hair model in the presence of d25-SDS and chitosan oligomer at the concentrations indicated in the graph. The yellow and blue panels represent the Au/thiol and the thiol/solution interfaces, respectively, with associated roughness. The zero value on the *x*-axis corresponds to the thiol/solution interface.

The peak is the same for the SDS/oligomer complex and for a pure SDS solution injected after exposure to chitosan, while rinses in between result in the appearance of a negative peak, i.e., of SLD lower than the bulk solution.

The explanation in this case is then that the oligomer contribution is hidden by SDS, but the latter is preferentially removed upon rinsing, making the (irreversibly) adsorbed chitosan visible. Please note that the thickness of this residual layer is very small compared to previous observations,^26,54^ probably because of the poor scattering contrast with the bulk, leading to a large uncertainty in the exact density profile. The result in this case is to be taken as an indication of a hydrogenous residue on the surface, and not quantitatively.

The interaction of the hydrophobic surfaces with the pure cationic surfactant is described in the ESI (Section S1), but it is worth mentioning here that a 20 cmc d42-CTAC solution resulted in an adsorbed layer on the EA thiol, while no adsorption was visible on the 18-MEA thiol. After the step in pure surfactant, two CTAC/oligomer ratios were studied, to mimic the SDS/oligomer ratio (chosen to simulate a diluted cosmetic formulation) in terms of both cmc and molar concentration of surfactant. In the case of the 18-MEA thiol (Figure 10), the profile obtained in the presence of the CTAC/chitosan complexes has a thickness which is compatible with the adsorption of a tilted monolayer of surfactant (the thickness values are 9 and 12 Å in the two steps), while the SLD value of the first ratio, being lower than pure d-CTAC, indicates the presence of solvent or hydrogenous species in the layer. In fact, in this solution the molar concentration of CTAC is just above 1 mM, so it is not in such a large excess as SDS compared to chitosan. A subsequent injection of a pure oligomer solution results in a decrease of the adsorption peak, but it is not possible here to define whether this is due to removal of adsorbed surfactant or adsorption of an amount of chitosan such that the SLD contributions cancel each other.

**Figure 10:**
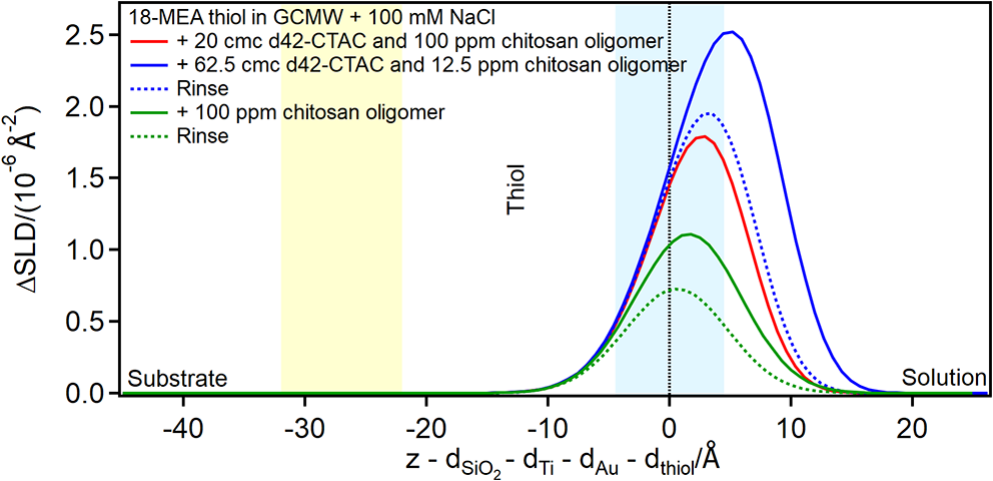
Subtracted depth profiles of a healthy hair model in the presence of d42-CTAC and chitosan oligomer at the concentrations indicated in the graph. The yellow and blue panels represent the Au/thiol and the thiol/solution interfaces, respectively, with associated roughness. The zero value on the *x*-axis corresponds to the thiol/solution interface.

On the EA thiol (see Figure S4 in ESI), addition of 20 cmc d42-CTAC with chitosan causes an increase in the adsorption of surfactant closer to the surface (increase in the SLD value, associated thus to an increase in deuterated species), that continues when the ratio of CTAC to chitosan is increased. This is the same as on the 18-MEA thiol surface. Again similarly to 18-MEA, a solution of pure chitosan oligomer causes a decrease in the SLD value of the adsorbed layer. Upon rinsing, instead, a thin layer of low SLD is left on the surface. This suggests the presence of a small amount of residual chitosan (strictly, due to the single contrast this could also be due to a larger residual chitosan amount (lower SLD) but with associated CTAC (higher, canceling, SLD)). Regarding the partially damaged hair model, the same steps were tested but in a different order (see sequence NR3 in Table 2). First, addition of pure chitosan oligomer makes the residual SDS peak asymmetric due to the SLD contrasts of the two materials (Figure 11). Upon rinsing, the layer becomes thicker but displays an SLD closer to the bulk. Injecting 2 cmc d25-SDS then restores the peak observed in SDS prior to rinsing.

**Figure 11:**
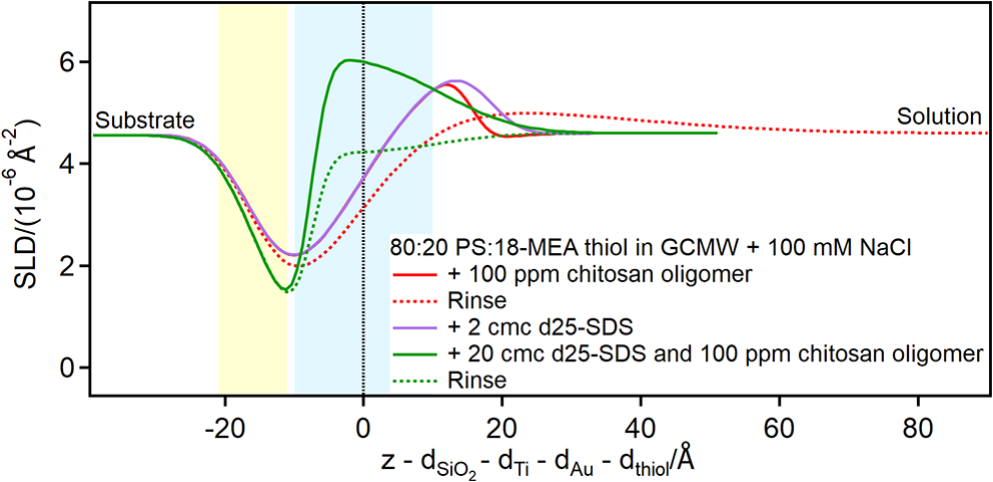
Depth profiles of the partially damaged hair model in the presence of d25-SDS and chitosan oligomer at the concentrations indicated in the graph. The yellow and blue panels represent the Au/thiol and the thiol/solution interfaces, respectively, with associated roughness. The zero value on the *x*-axis corresponds to the thiol/solution interface in pure solvent.

As previously for the adsorption of SDS and chitosan on the 18-MEA thiol surface, this result indicates that chitosan does interact with the surface, revealed as modifying subsequent adsorption of the surfactant (compare adsorption of 2 cmc SDS in Figures 11 and 6). The SLD profile of the SDS/chitosan complex, in contrast, is similar to that of pure SDS at a concentration of 20 cmc. The rinse, however, produces a different result as the residual layer has a lower SLD than the bulk and it is not shifted toward the solution, indicating intercalation in the thiol layer. Similarly, the profiles in the presence of both CTAC/chitosan ratios do not differ from the profile obtained for pure d42-CTAC (see Figure S7 in the ESI), suggesting no chitosan affinity to the surface from its mixtures with cationic surfactant. Upon rinsing, the adsorbed layers are apparently removed. Please note that no rinsing step was performed between adsorption of CTAC alone and in mixture with chitosan (see Table 2). The results then suggest that the bilayer formed in the presence of 20 cmc CTAC might saturate the surface and prevent adsorption of chitosan molecules from the solution injected afterward.

Finally, adsorption of CTAC/chitosan mixtures on the fully damaged hair model (pure PS) results in depth profiles of lower SLD and slightly larger thickness than pure CTAC (Figure 12—pure CTAC adsorbs as a hydrated bilayer). In the presence of 62.5 cmc d42-CTAC/12.5 ppm oligomer, the SLD profile suggests an apparently clean surface, but upon rinsing, a small negative peak related to the presence of hydrogenous material appears. This indicates that chitosan was indeed adsorbed, associated with d42-CTAC at a ratio such that their SLDs of opposite sign mask each other. Rinsing preferentially removed the surfactant due to its higher solubility, eventually revealing the presence of coadsorbed polyelectrolyte.

**Figure 12:**
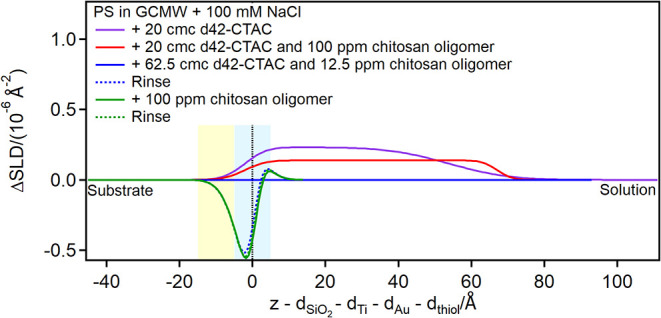
Subtracted depth profiles of the fully damaged hair model in the presence of d42-CTAC and chitosan oligomer at the concentrations indicated in the graph. The yellow and blue panels represent the Au/thiol and the thiol/solution interfaces, respectively, with associated roughness. The zero value on the *x*-axis corresponds to the thiol/solution interface.

Subsequent injection of pure oligomer does not modify the depth profile. Evidence for the adsorption of chitosan oligomer was obtained, however, from AFM measurements (Figure 13c—the figure displays also the results relative to 18-MEA and mixed thiol surfaces).

**Figure 13:**
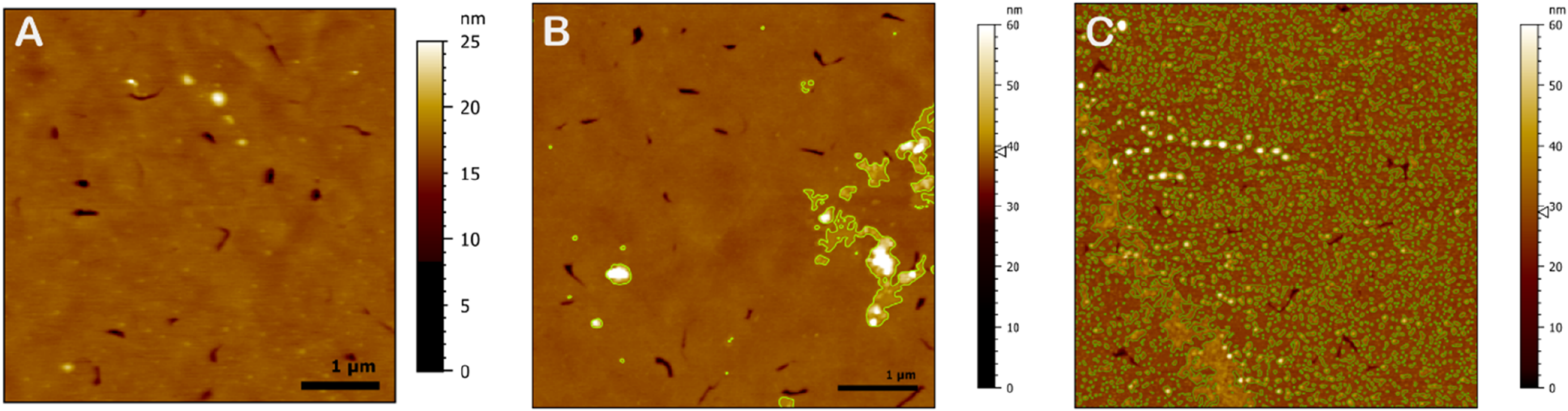
Examples of AFM images of (a) 18-MEA thiol, (b) 50:50 PS:18MEA thiol, and (c) PS surfaces in the presence of 100 ppm chitosan oligomer. Detected particles (i.e., adsorbed aggregates) are colored in green. The dark spots are holes in the gold layer.

Analysis of AFM images confirms directly the adsorption of the chitosan that could otherwise only be indirectly inferred from NR. The high level of hydration indicated by the slab analysis in the NR data is now revealed as a low density of patches of characteristic thickness (remembering that the slab analysis is an average composition in-plane). The particle coverage is ≈4% on the hydrophobic surface, and increases to ca. 7% on the partially damaged hair model, reaching an area of ≈16% on the fully damaged hair model, while the median height is ca. 2 nm in all cases (although some particles are several tens of nm high).

The two techniques are thus complementary in clarifying the adsorption scenarios: chitosan has a poor scattering contrast and it is mostly not directly visible in NR data, while its size and tendency to adsorb as discrete particles make it easily visualized by AFM. In contrast, the SDS scattering contribution is clear in NR, but its presence is less obvious in AFM images (see ESI). Putting together the information on the two techniques, it can be deduced that chitosan adsorbs on both 18-MEA and damaged hair models (with a preference for the negatively charged PS surface) and that SDS forms a homogeneous layer on the surfaces, adsorbing in between chitosan particles thus reducing the apparent surface coverage extracted from sizes in AFM images.

### 3.4 Adsorption of Synthetic vs Natural Polymers

Finally, adsorption of chitosan polymer and of pDADMAC was studied, and in both cases SDS was again adsorbed afterward to mimic the interaction of an adsorbed layer with a shampoo formulation. Due to the contrast limitations, adsorption of chitosan polymer was not directly observed on any surface (Figures S34, S61, S85, S90d in the ESI), but its presence on the surface is instead revealed by the increased adsorption of SDS injected afterward (Figures 14, 15, and 16; for EA, Figure S5 is in the ESI).

**Figure 14:**
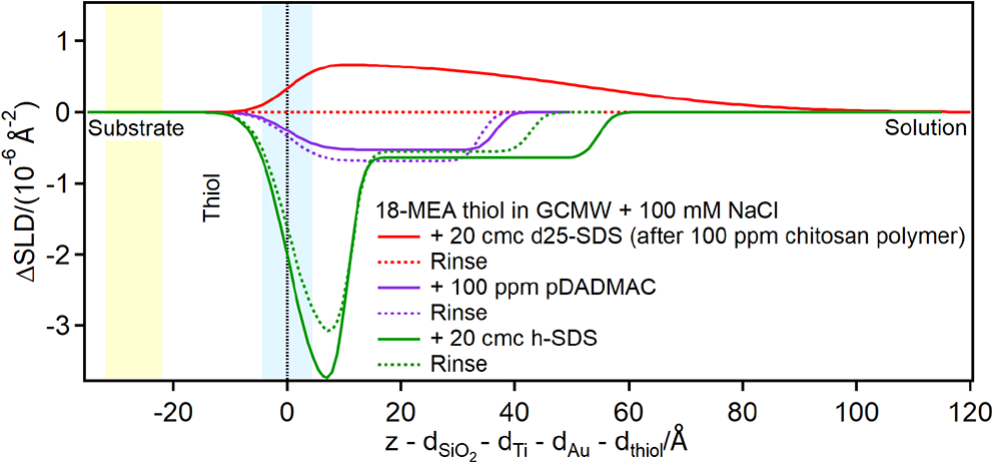
Subtracted depth profiles of a healthy hair model in the presence of chitosan polymer or pDADMAC and SDS at the concentrations indicated in the graph. The yellow and blue panels represent the Au/thiol and the thiol/solution interfaces, respectively, with associated roughness. The zero value on the *x*-axis corresponds to the “clean” thiol/solution interface.

**Figure 15:**
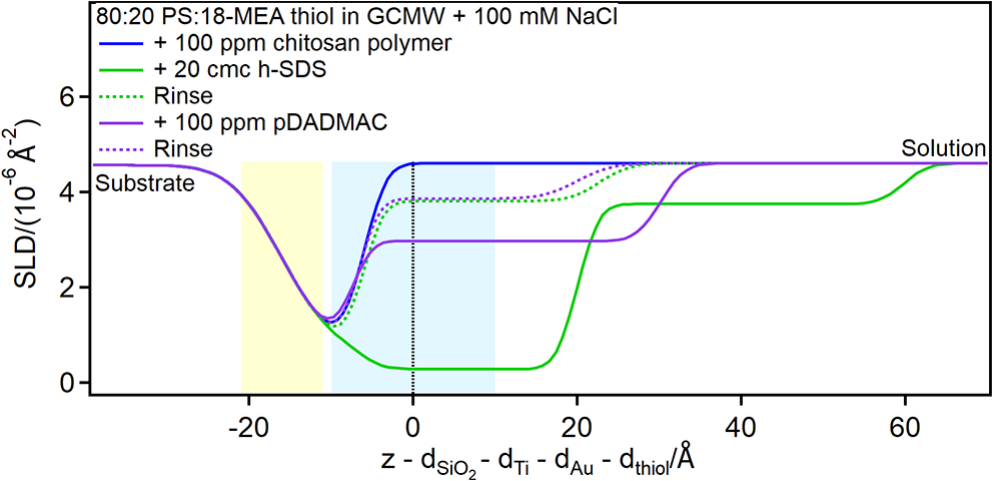
Depth profiles of the partially damaged hair model in the presence of chitosan polymer or pDADMAC and SDS at the concentrations indicated in the graph. The yellow and blue panels represent the Au/thiol and the thiol/solution interfaces, respectively, with associated roughness. The zero value on the *x*-axis corresponds to the thiol/solution interface in pure solvent.

**Figure 16:**
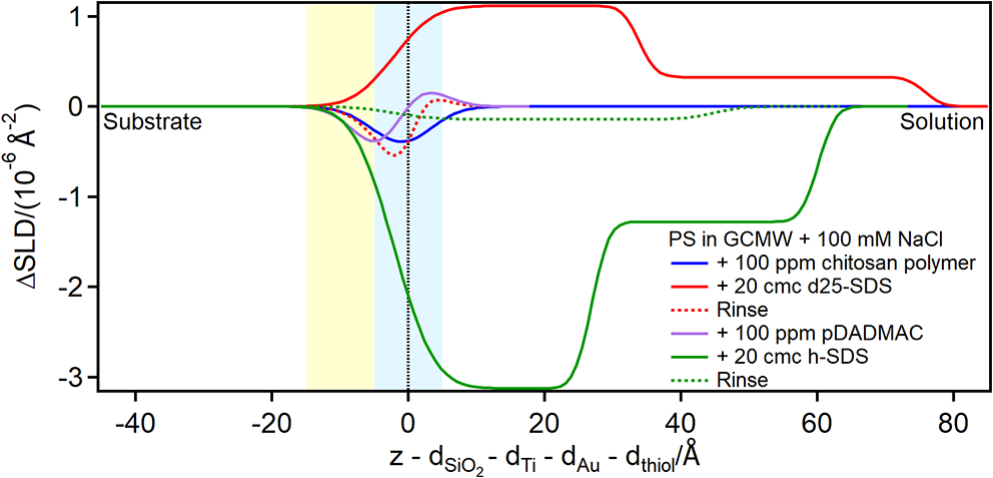
Subtracted depth profiles of the fully damaged hair model in the presence of chitosan polymer or pDADMAC and SDS at the concentrations indicated in the graph. The yellow and blue panels represent the Au/thiol and the thiol/solution interfaces, respectively, with associated roughness. The zero value on the *x*-axis corresponds to the thiol/solution interface.

The resulting adsorbed SDS layer is several tens of Å thick, much larger than a bilayer, indicating it is interacting with polymer present on the surface. On the damaged hair models, in particular, two layers are distinguished on top of the thiol surface: the first layer resembles the pure surfactant (please note that hydrogenous SDS was used in the case of the 80:20 mixed sample, so the profile is mirrored compared to pure d25-SDS), while the second layer has a SLD closer to the bulk, suggesting the presence of chitosan extending in solution. This structure is reminiscent of the one formed by an SDS/oligomer complex on a pure, hydrophilic PS surface.^26^ Upon rinsing, a residue is left on the damaged hair models, but not on the 18-MEA thiol surface. A possible explanation is that in the latter case SDS is not closely associated with the surface, so it can complex chitosan and remove it from the surface, despite the poor solubility of the polymeric form at pH close to neutrality. This is the desired outcome from a cosmetic perspective, as the chitosan residue would protect the damaged regions on the hair fiber, while it is not needed that the same occurs on undamaged hair.

In contrast, the less hydrated pDADMAC adsorption on the model surfaces can be directly observed, in layers which are about 30 Å thick, in agreement with literature data.^17^ The adsorption is mostly irreversible, but interestingly, on the partially damaged hair model, the rinse restores the previous profile, suggesting that pDADMAC is not well retained on the surface. This may be explained by the different characteristics of the two polyelectrolytes: it is known that chitosan is more rigid (the persistence length is in the range 5–30 nm,^59,60^ while for pDADMAC it is 2.5 nm^66^) and has a low charge density,^17,59^ and these factors, together with the aforementioned low solubility, may contribute to the observed stronger interaction, compared to pDADMAC, with the mixed thiol surface. SDS added subsequently interacts with the pDADMAC layer and adsorbs on the surface (the SLD value of adsorbed pDADMAC suggests that the layer is highly hydrated, likely organized in separate aggregates with patches of uncovered surface in between), being eventually retained on the hydrophobic surfaces but not on the damaged hair model. These results are in agreement with those obtained by Coscia *et al.* ^17^ using a computational approach. Specifically, they observed that a quaternary ammonium polymer (as pDADMAC) does not contact an 18-MEA surface, but rather interacts with the surfactant chains, which preferentially adsorb with their hydrocarbon tails on the surface. Instead, a polysaccharide is in contact with the surface, but also extends more in solution. Interestingly, they did not observe adsorption of the positively charged polyelectrolyte groups on a damaged hair surface, since negatively charged sulfonate moieties were neutralized by sodium ions. Likewise, our data suggest a lower adsorption of pDADMAC on the PS compared to the 18-MEA thiol surface.

## 4 Conclusions

This paper offers an overview of the interaction of different surfactants and polyelectrolytes on hair-mimetic surfaces. Four models were produced, two (18-MEA and EA thiols) mimicking healthy hair surfaces and two (80:20 PS:18-MEA thiol and pure PS) representing two stages of damage of the hair surface. The effect of surface features, surfactant charge, polyelectrolyte type, and order of injection of the adsorbing species were compared. The main conclusions can be summarized as follows:

- these biomimetic thiols adsorb differently compared to conventional ones. Counterintuitively the inclusion of the amide group on the fatty acid chain, which renders the molecules more biomimetic actually influences the thiol layer formation negatively due to the presence of physisorbed, intercalated species. Only neutron reflectometry is capable of unambiguously detecting this adsorption anomaly;
- the partially damaged model surface presents patches of hydrophobic and hydrophilic character, and thus provides an excellent mimic. Two mechanisms of surfactant adsorption thus take place: on the hydrophobic patches, the hydrocarbon tails adsorb hydrophobically and intercalate with 18MEA chains, while on the hydrophilic patches the interaction is mainly electrostatic and a bilayer is formed;
- pDADMAC adsorption is clear on all the models, but interestingly the interaction seems stronger on the healthy hair models compared to the damaged hair models;
- chitosan adsorbs in highly hydrated layers and its presence is thus difficult to isolate directly, but it is unambiguously revealed by the dramatically increased adsorption of SDS;
- the adsorption of surfactant/chitosan oligomer mixtures (at ratios of cosmetic relevance) is mostly dominated by the surfactant, but in the case of the damaged hair models, the complex adsorbs and the chitosan oligomer is preferentially retained.

The work shows the capabilities of NR in describing surface interactions hierarchically, and provides detail that would not be evident using other surface techniques. It reveals a wealth of information in terms of how to design molecules for biomimicry in a thiol film, and also how to control thiol composition to achieve realistic models for damage. Importantly it suggests that the polysaccharide chitosan may actually provide higher affinity to the hair surface than the conventional polymer it may eventually replace.

## Supplementary Material



## Data Availability

Sequence NR1 (data collected on INTER): 10.5286/ISIS.E.RB2210338. Sequence NR2 (data collected on FIGARO): http://doi.ill.fr/10.5291/ILL-DATA.9-13-1052. Sequence NR3 (data collected on SuperADAM): http://doi.ill.fr/10.5291/ILL-DATA.CRG-2962.

## Notes

Structure of healthy hair model surfaces, XPS data, additional AFM data, and fitted NR curves (PDF)

## ◆

Department of Physics and Astronomy, Materials Physics, Uppsala University, SE-751 20 Uppsala, Sweden

## ¶

Neutron Scattering Division, Oak Ridge National Laboratory, Oak Ridge, 37831 Tennessee, United States.
