# Supplementary material for: Adsorption of Surfactants and Polymers to Biomimetic Hair Model Surfaces
Source: Langmuir. 2026 Mar 6;42(11):7809–21. doi: 10.1021/acs.langmuir.5c06252 (PMC13019672; doi:10.1021/acs.langmuir.5c06252)
Supplement: Supplementary file 1 [file la5c06252_si_001.pdf]

Supplementary Information - Adsorption of surfactants and polymers to biomimetic hair model surfaces

Serena Cozzolino,[a b †] Philipp Gutfreund,[b] Inger Odnevall,[a] Raam Ibrahim,[a] Alexei Vorobiev,[b c] Rebecca J. L. Welbourn,[d §] Francesca Zuttion,[e] Andrew Greaves,[e] Gustavo S. Luengo,[e *] and Mark W. Rutland[a f g h *]

[a] Division of Surface and Corrosion Science, School of Engineering Sciences in Chemistry, Biotechnology and Health, KTH Royal Institute of Technology, SE-100 44 Stockholm, Sweden

[b] Institut Laue-Langevin, 71 avenue des Martyrs, CS 20156, 38042 Grenoble cedex 9, France

[†] current address: Department of Physics and Astronomy, Materials Physics, Uppsala University, SE-751 20 Uppsala, Sweden

[c] Department of Physics and Astronomy, Materials Physics, Uppsala University, SE-751 20 Uppsala, Sweden

[d] ISIS Pulsed Neutron and Muon Facility, Rutherford Appleton Laboratory, Didcot, Oxfordshire OX11 0QX, UK

[§] current address: Neutron Scattering Division, Oak Ridge National Laboratory, Oak Ridge, Tennessee 37831, USA

[e] L'Oréal Research and Innovation, 1 avenue Eugène Schueller, 93600 Aulnay-sous-Bois, France

[f] Bioeconomy and Health Department, Materials and Surface Design, RISE Research Institutes of Sweden, SE-114 28 Stockholm, Sweden

[g] School of Chemistry, University of New South Wales, Sydney, NSW 2052, Australia

[h] Laboratoire de Tribologie et Dynamique des Systèmes, École Centrale de Lyon, 69134 Ecully CEDEX, France

[*] corresponding authors: mark@kth.se (M. W. R.), gluengo@rd.loreal.com (G. S. L.)

S1 Structure of the healthy hair model surfaces

The two healthy hair model surfaces, i.e., 18-MEA and EA thiols, showed an unexpected structure after the preparation, very different from conventional thiol assemblies. Fitting of the neutron reflectometry (NR) data of the first measurement in pure solvent revealed a layer thickness larger than the molecular length (≈30 Å). Then, when either SDS or CTAC were adsorbed, a decrease in the thiol thickness was observed: data fitting was not possible in the usual way, i.e., fixing the parameters of the thiol layer from the NR curve in pure solvent and adding one slab to fit adsorbed species. Results for the 18-MEA thiol are in Figure S1.

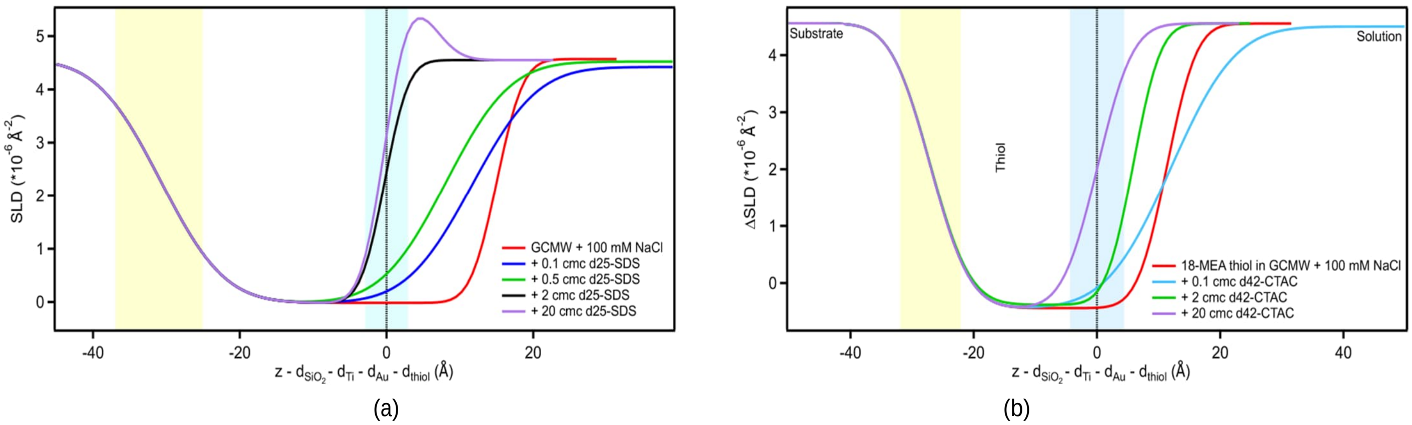

Fig. S1 Depth profiles relative to the 18-MEA thiol in the presence of a) d25-SDS or b) d42-CTAC, at the concentrations indicated in the graphs. The yellow and blue panels represent the Au/thiol and thiol/solution interfaces, respectively, with associated roughness. The zero level on the x-axis has been set at the "clean" thiol/solution interface.

The final value of the layer thickness (30 ±1 Å) is obtained in the presence of 2 cmc d25-SDS, or 20 cmc d42-CTAC. After these steps, the NR data in each sequence can be fitted using this structure as starting point and adding one (or, in some cases, two)

slab(s) to describe the adsorbed layer. In the case of SDS, this can be applied already in the presence of 20 cmc surfactant, and a small peak is visible in the relative depth profile in Figure 1(a). Assuming that all the adsorbed SDS is represented by the peak, a surface excess[1] of 0.2 nmol cm^{-2} can be calculated. This corresponds to about half of an adsorbed monolayer[2,3,4]. Considering scattering contrast arguments, though, it is possible that a full monolayer is adsorbed, but it is partly hidden by thiol molecules still interacting with the surface, so that the total effect is for the layer to have the same SLD as the bulk. Likewise, no adsorption peak is visible in the presence of the explored concentrations of CTAC, but the two species (hydrogenous thiol and deuterated surfactant) may be intercalating at such ratio that the total SLD matches the bulk. Nonetheless, the thiol layer obtained in the presence of 2 cmc d25-SDS in one case, and 20 cmc d42-CTAC in the other case, being compatible with a bound monolayer on gold, was considered as the reference SAM for the system and used to obtain the difference SLD profiles shown in the paper.

The EA thiol also showed an unexpected structure, but its behaviour was different from the 18-MEA thiol system. Firstly, only one of the two systems (the one used in sequence NR2) had a thickness larger than a monolayer in pure solvent. EA thiol profiles in the presence of SDS and CTAC are in Figure S2.

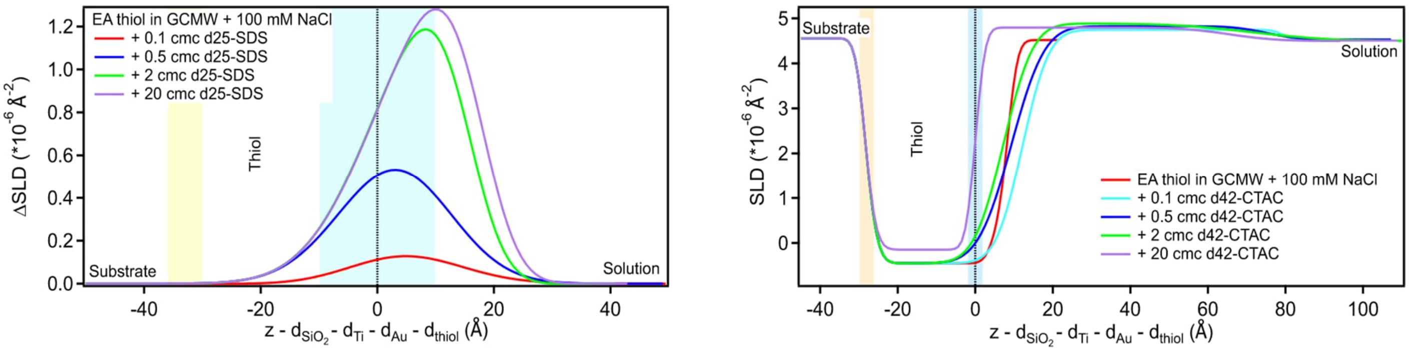

(a) (b)

Fig. S2 Depth profiles relative to the EA thiol in the presence of a) d25-SDS (subtracted profiles) or b) d42-CTAC, at the concentrations indicated in the graphs. The yellow and blue panels represent the Au/thiol and thiol/solution interfaces, respectively, with associated roughness. The zero level on the x-axis has been set at the "clean" thiol/solution interface.

The thiol layer in the system exposed to SDS has a constant thickness throughout the adsorption sequence, but it also shows a significantly higher roughness compared to the other model surfaces. As the thiol layer is unmodified for increasing concentrations of SDS, difference SLD profiles can be obtained. The surface excess[1] is 0.1, 0.3, 0.5 and 0.6 nmol cm^{-2}, for 0.1, 0.5, 2 and 20 cmc respectively (please note that the values in the presence of 20 cmc d25-SDS suggest that the adsorbed structure is larger than a monolayer, but the higher roughness, comparable to thickness values, may have introduced artefacts in the profiles). Instead, the surface produced for the other NR experiment has a starting thickness of 40 Å, which, similar to the 18-MEA thiol system, decreases for increasing concentrations of d42-CTAC. In this case, though, at intermediate concentrations (0.5 and 2 cmc), only a slight decrease is observed, and a diffused adsorbed layer is present above the thiol. At 20 cmc d42-CTAC, the thickness of the thiol layer is reduced to that of a monolayer. The SLD value of the adsorbed layer suggests, in all the cases, that it contains a high percentage of water, and/or hydrogenous material. The fitted thickness is larger than a bilayer of CTAC molecules, indicating it may contain the excess thiol that is being removed from the surface.

A scenario that has been explored to explain the different behaviour of the two freshly prepared thiols in the presence of CTAC is shown in Figure S3, together with a simulation of the model implemented on the NR dataset of the EA thiol in pure solvent.

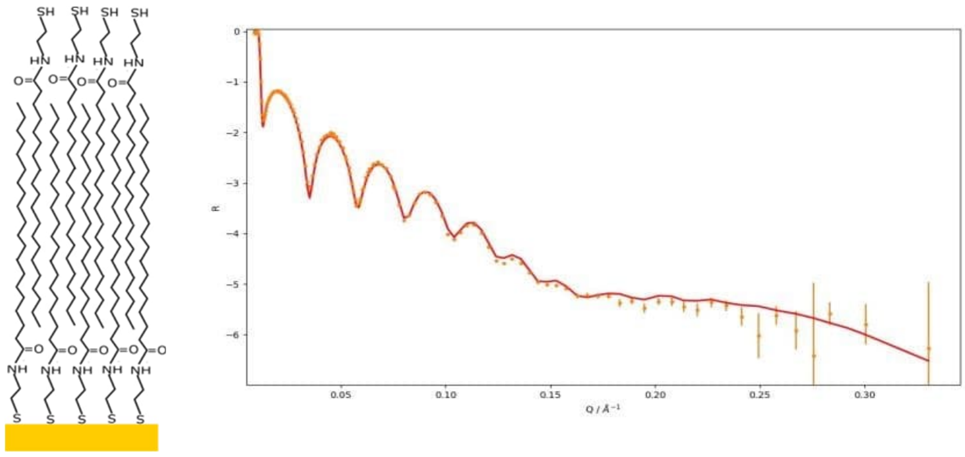

(a) (b)

Fig. S3 a) Schematic drawing of the hypothesized EA thiol structure. b) Simulated NR curve with the thiol layer described by three slabs (spacer/amide on the surface, hydrocarbon chains, spacer/amide in the bulk). Orange dots are the experimental points for the surface in pure solvent.)

Unfortunately, the fit run on this model does not allow to unambiguously adopt this structure to describe the EA thiol layer, but the presence of hydrophilic groups could explain the apparently stronger interaction of d42-CTAC with the surface. This may be favoured on EA compared to 18-MEA thiol because of the lack of the methyl branch and the consequently different chain packing.

S1.1 Adsorption on the EA thiol surface

As shown in Figure S4(a) and commented in the main text, the injection of a SDS/chitosan oliogmer complex after exposure of the EA thiol surface to pure SDS does not result in additional adsorption from the solution. Instead, when mixed with CTAC, the oligomer has the effect of condensing the deuterated species towards the surface (as Figure S4(b) shows, the thickness of the adsorbed layer decreases and its SLD i.e., the intensity of the peak, increases), but the following rinse restores the profile obtained for 20 cmc d42CTAC. Addition of a pure chitosan solution results in an adsorbed layer of similar thickness but lower SLD. This could be due either to removal of adsorbed molecules or to interaction of chitosan with the adsorbed layer and compensation of the SLD values of hydrogenous and deuterated species, the latter being more likely as a rinsing step at this stage leaves on the surface an hydrogenous residue, albeit poorly defined.

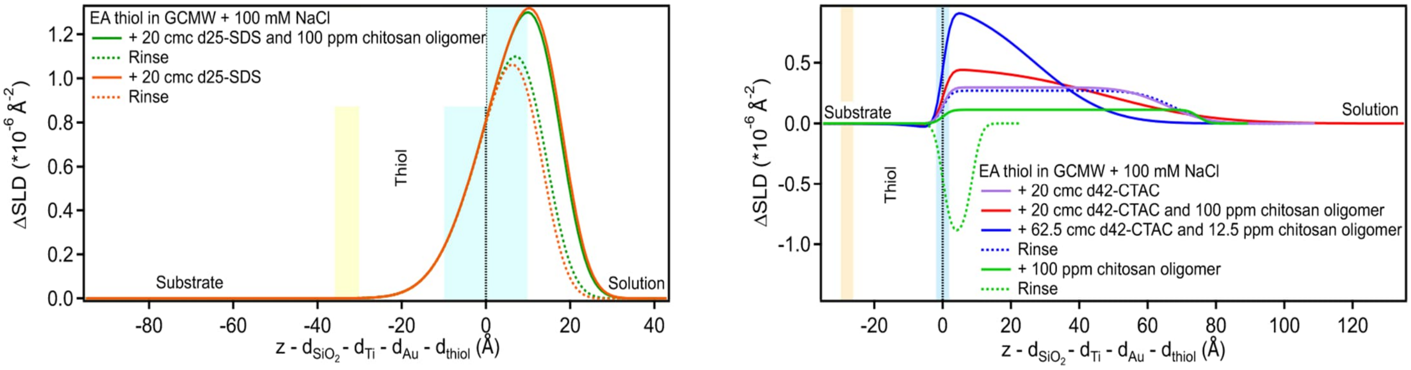

(a) (b)

Fig. S4 Depth profiles relative to the EA thiol in the presence of chitosan oligomer and a) d25-SDS or b) d42-CTAC, at the concentrations indicated in the graphs. The yellow and blue panels represent the Au/thiol and thiol/solution interfaces, respectively, with associated roughness. The zero level on the x-axis has been set at the "clean" thiol/solution interface.

In the case of the polymeric chitosan (Figure S5, the adsorbed layer appears thin compared to previous findings[5], but this can mainly be due to poor contrast (due to the high percentage of solvent in chitosan adsorbed layers and/or the presence of deuterated residues that counterbalance its contribution to scattering) as a subsequent injection of d25-SDS forms a thick layer above the thiol. Similarly, the presence of pDADMAC on the surface helps subsequent adsorption of SDS. The adsorption is irreversible, but it is not possible to define the amount of polyelectrolyte or surfactant in the final layer.

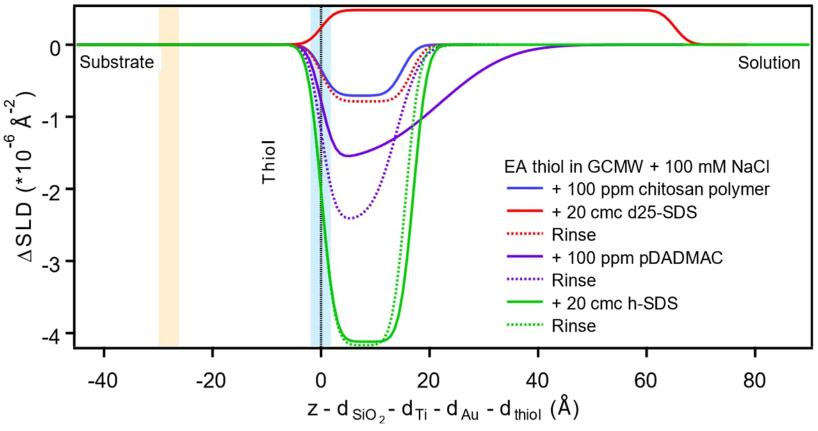

Fig. S5 Depth profiles relative to the EA thiol in the presence of chitosan polymer or pDADMAC and SDS, at the concentrations indicated in the graphs. The yellow and blue panels represent the Au/thiol and thiol/solution interfaces, respectively, with associated roughness. The zero level on the x-axis has been set at the "clean" thiol/solution interface.

S2 Adsorption on the partially damaged hair model surface

In the case of the partially damaged hair model, i.e., the 80:20 PS:18-MEA thiol surface, data in the main text are presented as SLD profiles without subtraction of the thiol layer in pure solvent. This is because subtraction in this case gave rise to artefacts in the profiles and implied the use of a model which may not be valid for all the steps. Throughout the adsorption sequence, the thickness of the thiol layer oscillates between two values: 9 (±1) Å, that corresponds to a compact portion formed by the spacer/amide part of the 18-MEA thiol and the PS molecules, or 15 (±1) Å, which represents the full thiol layer, comprised of the hydrocarbon chains protruding from the portion containing PS. When the thiol layer displays the former thickness, strictly two explanations are possible: the adsorbed species is intercalating between 18-MEA chains, modifying the SLD of this portion, or it is adsorbing on top of the thiol layer, condensing the 18-MEA chains closer to the surface. Moreover, considering the patchiness of the layer, adsorption does not occur with a homogeneous mechanism on the whole surface but different scenarios are possible, as illustrated in the main text. An example of a subtracted profile is in Figure S6(a). The same Figure also shows a fit of the thiol surface in pure solvent, described with two slabs (one for the "invariable" part of about 9 Å, and the second one for the protruding 18-MEA chains, that can change when molecules intercalate). The fit has a slightly lower chi2 compared to the one-slab model, but as the corner plot in Figure S6 shows, the obtained solution is not unambiguously determined. It is worth mentioning here that for this sample the fitting model is more complex, as the experiment was performed on a monochromatic neutron reflectometer. As described in the ESI of a previous publication[6], overillumination issues, variable along the NR curve, play an important role here and must be considered during the fitting.

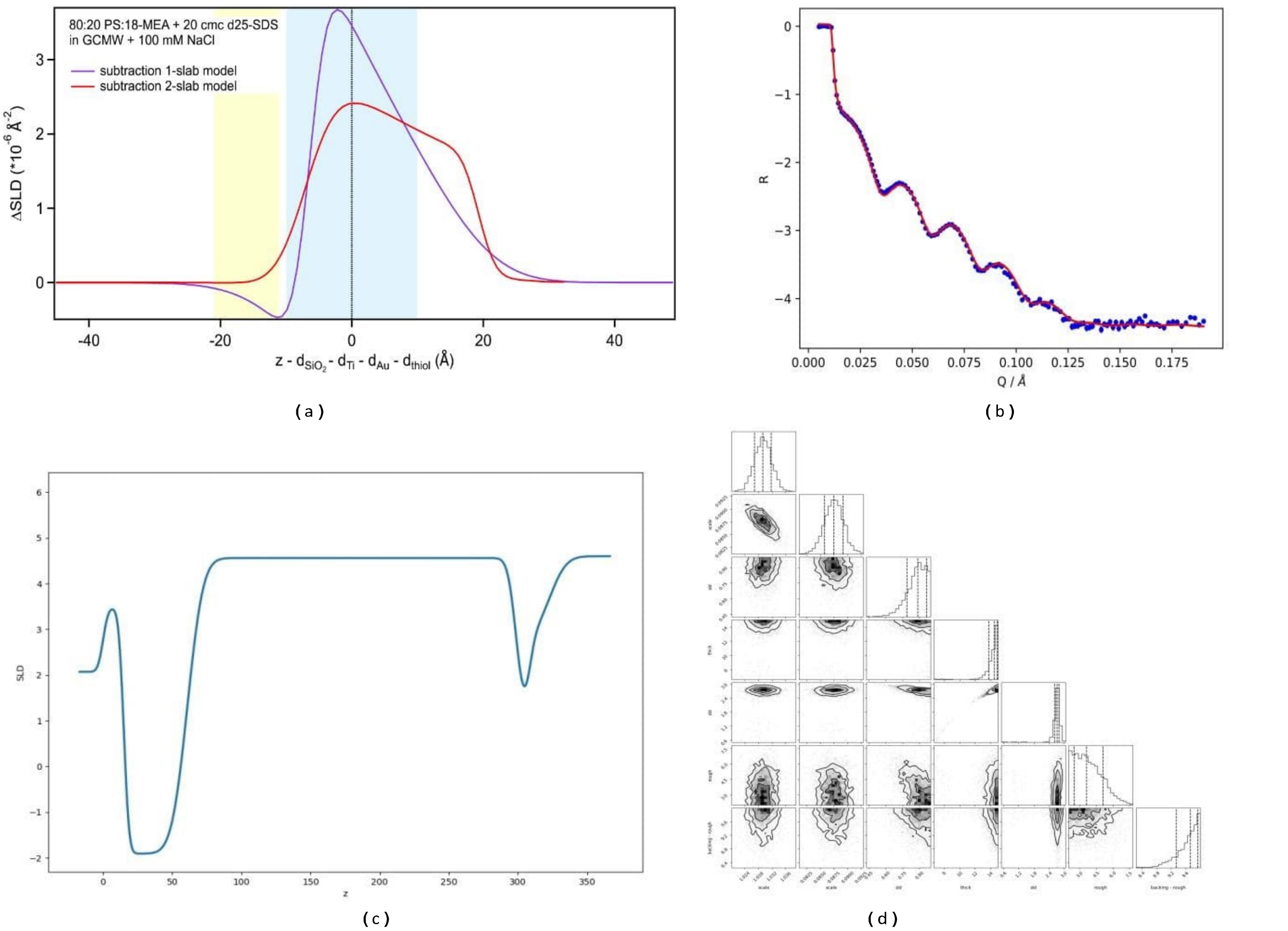

Fig. S6 a) Depth profiles relative to 80:20 PS:18-MEA thiol in the presence of 20 cmc d25-SDS, after subtraction of the SLD profile of the thiol layer in pure solvent. The yellow and blue panels represent the Au/thiol and thiol/solution interfaces, respectively, with associated roughness. The zero level on the x-axis has been set at the thiol/solution interface in pure solvent. b) Fitted NR data of the mixed thiol surface in GCMW and 100 mM NaCl with a 2-slab model, and corresponding SLD profile (c) and corner plot from MCMC analysis (d).

S2.1 Adsorption of the CTAC/chitosan oligomer complexes

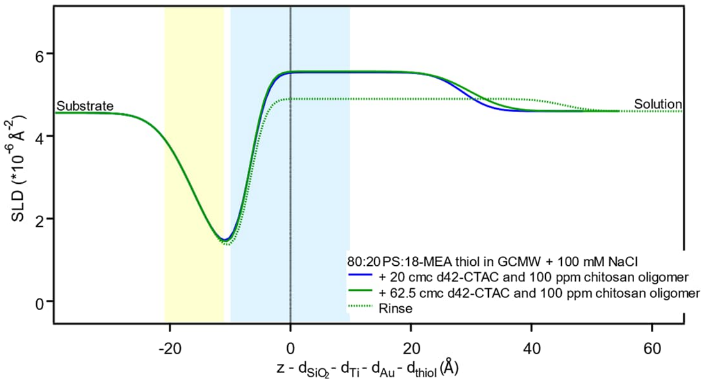

Fig. S7 Depth profiles relative to the partially damaged hair model in the presence of d42-CTAC and chitosan oligomer, at the concentrations indicated in the graphs. The yellow and blue panels represent the Au/thiol and thiol/solution interfaces, respectively, with associated roughness. The zero level on the x-axis has been set at the thiol/solution interface in pure solvent.

S3 X-ray Photoelectron Spectroscopy (XPS) measurements

X-ray photoelectron spectroscopy (XPS) was used to define the relationship between thiol ratios in solution and on the surface. The surface ratio can then be compared to XPS measurements done on bleached human hair[7]. XPS is a powerful technique that can distinguish different types of bond by their binding energy. Specifically in this case, the distinction between the S-O bonds and other sulphur species was of interest. By using the data collected on human hair samples[7], an approximation can be made for the biomimetic surface, in this case produced by mixing PS with the commercially available long-chain thiol ODT (octadecanethiol). Assuming that the sulphonate groups on the model surfaces represent the SO_x groups on human hair, the same "amount" of hair damage can be recreated on the biomimetic surfaces. Reference values for sulphur species on bleached human hair (taken in the middle of the fibre) are:

- SO_x: 1.9 atom %
- R-S-S-R: 0.7 atom %
- R-SO3H: 0.5 atom %

From this, the ratio between SO_x groups and total sulphur is 0.613. When performing the measurements on the biomimetic gold surfaces, two results were obtained. Firstly, data confirmed that the thiol ratio in solution has a non-linear relationship to the thiol ratio on the surface (see blue curve in Figure S8). Secondly, by fitting the data with an exponential curve, the results can give an approximate value (the fit is not perfect) of the ratio to use in solution depending on the desired ratio on the surface.

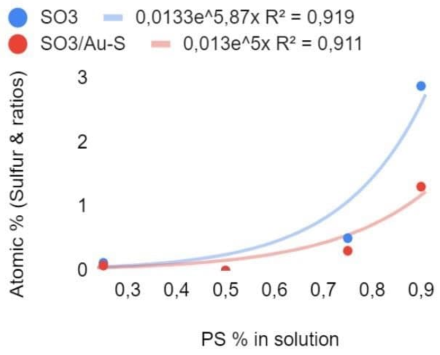

Fig. S8 XPS results on ODT/PS biomimetic surfaces. Blue dots, atomic percentage of sulphur in sulphonate groups. Red dots, ratio between atomic percentage of sulphur in sulphonates and total sulphur. Solid lines indicate the exponential fitting of the datapoints.

From the equation obtained by fitting the points relative to the SO_3/Au-S ratio, the needed percentage of PS in solution to represent a damaged hair surface is therefore $ln(0.613/0.013)/5=0.771$.

S4 Atomic Force Microscopy (AFM) data in the presence of SDS and SDS/chitosan oligomer complex

In the presence of SDS and SDS/oligomer complex, the median height of the visible aggregates does not vary much compared to the pure chitosan shown in the main text, while the particle coverage seems to decrease. This could be due to less adsorption, but if the surfactant were to adsorb and carpet the surface in between chitosan moieties, this could mask the smaller particles, resulting in an apparently lower coverage. The trend of increasing coverage with increasing surface hydrophilicity (seen in pure chitosan) is maintained on washing with SDS. Instead, in the presence of the SDS/chitosan (pre-mixed) complex, the highest coverage (4%) is observed in the case of the mixed PS:18-MEA thiol surface, and, unexpectedly, the AFM data actually suggests lowest coverage (2%) on PS.

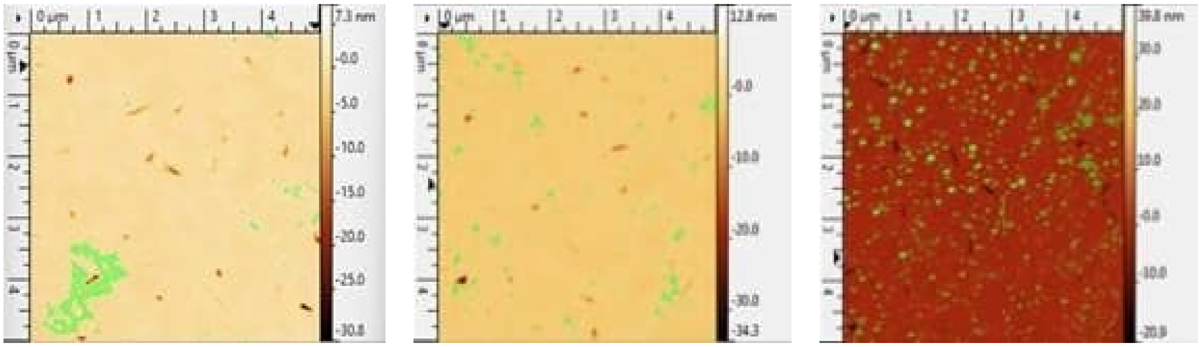

(a) (b) (c)

Fig. S9 Example of AFM images of a) 18-MEA thiol, b) 50:50 PS:18-MEA thiol and c) PS surfaces in the presence of 2 cmc h-SDS. Detected particles (i.e., adsorbed aggregates) are coloured green. The dark spots are holes in the gold layer.

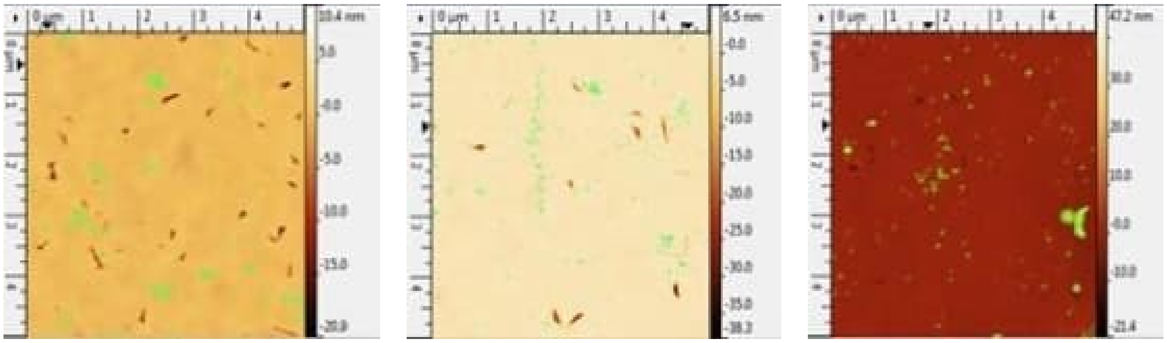

(a) (b) (c)

Fig. S10 Example of AFM images of a) 18-MEA thiol, b) 50:50 PS:18-MEA thiol and c) PS surfaces in the presence of 20 cmc h-SDS and 100 ppm chitosan oligomer. Detected particles (i.e., adsorbed aggregates) are coloured green. The dark spots are holes in the gold layer.

Table S1 Analysis of AFM images of the thiol surfaces in the presence of SDS or SDS/chitosan complex. The reported values are the average of the medians of two samples

	SDS		SDS/chitosan	
	Coverage (%)	Height (nm)	Coverage (%)	Height (nm)
18-MEA thiol	4	1.7	4	3
50:50 PS:18-MEA thiol	5	2.2	4	2.5
PS	5	3.1	2	3

Table S2 Particle analysis for the 18-MEA thiol surfaces in the presence of chitosan, SDS or SDS/chitosan complex. Images are numbered per sample and adsorbing species. More images were normally collected for each region of interest (ROI). Total projected area and median height were then averaged to obtain the values in Table S1

Sample	ROI	Image number	Number of grains	Mean grain size (nm)	Total projected area (%)	Minimum height (nm)	Maximum height (nm)	Median height (nm)
100 ppm chitosan oligomer								
1	1	1	44	39	0.44	1.3	7	2.1
	1	2	130	39	1.1	1	6.5	1.3
	2	3	34	77	1.4	2.1	18.2	4.4
	2	4	124	42	1.4	1	10	1.4
	3	5	313	38	2.3	1.5	16.6	2
	4	6	72	28	0.29	1.3	17.7	1.5
	4	7	60	34	0.43	1.2	13	1.9
2	1	1	428	27	1.7	1	13.6	1.4
	1	2	810	25	2.3	1	5.6	1.2
	2	3	403	29	1.8	1.2	21	1.6
	2	4	357	28	1.5	1.2	18	1.7
	3	5	1375	36	11	2	41	3
	3	6	1566	40	17	2	24	3
2 cmc SDS								
1	1	1	146	35	3.4	1.2	7.2	2.2
	1	2	352	34	4.5	1	30	1.7
	1	3	397	28	1.6	1	20	1.2
	2	4	96	34	0.7	1.2	5.1	1.4
	2	5	143	32	0.8	1	6.7	1.2
	3	6	280	52	11	1.2	7.7	2
	3	7	202	43	8.7	1.2	9.6	2.3
	4	8	165	34	1.4	1	31	1.5
2	1	1	419	28	1.5	1.2	11.5	1.6
	1	2	699	26	2.4	1	28	1.2
	1	3	357	27	1.4	1.2	26	1.5
	2	4	695	28	3.2	1	13	1.4
	2	5	787	30	4.2	1	17	1.5
	2	6	546	27	2.1	1	20	1.4
	3	7	899	35	9.5	1.2	36	3.2
	3	8	805	32	5.4	1.2	48	2.1
20 cmc SDS + 100 ppm chitosan oligomer								
1	1	1	253	38	2.3	1	10.4	1.9
	1	2	218	58	5.6	1.5	43.2	4.3
	1	3	283	31	1.4	1	7.9	1.2
	2	4	291	32	1.6	1	22	1.3
	2	5	229	32	1.3	1	9	1.2
	3	6	161	36	1.5	1	18	2
2	1	1	364	43	5.8	2	94	5.3
	1	2	242	47	3.9	1.5	50	6.9
	2	3	487	32	3	1	18.4	2.2
	2	4	601	44	12	1	115	3.5
	3	5	468	28	2.2	1	37	1.3
	3	6	382	61	2.1	1.2	104	4

Table S3 Particle analysis for the PS thiol surfaces in the presence of chitosan, SDS or SDS/chitosan complex. Images are numbered per sample and adsorbing species. More images were normally collected for each region of interest (ROI). Total projected area and median height were then averaged to obtain the values in Table S1

Sample	ROI	Image number	Number of grains	Mean grain size (nm)	Total projected area (%)	Minimum height (nm)	Maximum height (nm)	Median height (nm)
				100 ppm chitosan oligomer				
1	1	1	2528	33	17	2.5	60	4.6
	1	2	2540	34	16	2.5	47	4.8
	2	3	1523	28	6	3	69	4
	2	4	1817	27	6	3	87	4
	3	5	1567	32	8	3	30	4.5
	3	6	1295	31	8	3	154	5.2
2	1	1	3630	36	26	1	32	1.8
	1	2	3359	38	27	1	9	1.7
	1	3	3197	38	27	1	21	1.7
	2	4	2331	49	26	1	15	3.4
	2	5	2017	50	24	1	130	3.8
	2	6	3233	34	18	1	11	2
	3	7	3192	33	20	1	31	1.4
	3	8	2988	32	18	1	19	1.4
				2 cmc SDS				
1	1	1	808	44	8.5	2	44	3.8
	1	2	648	40	5.8	2	40	4
	1	3	824	42	8.6	2	85	3.7
	2	4	1153	42	11	2	41	3.6
	2	5	1021	43	11	2	36	3.8
	2	6	1174	39	10	2	67	4.1
2	1	1	274	29	1.7	1	34	1.3
	1	2	283	28	1.5	1	14	1.2
	1	3	298	27	1.2	1	14	1.2
	2	4	279	26	1.6	1	26	1.6
	2	5	320	32	3.3	1	95	9.2
	2	6	344	32	2.9	1	108	3
	3	7	240	26	1.1	1	21	1.3
	3	8	305	26	1.1	1	13	1.2
	3	9	342	25	1.3	1	10	1.2
				20 cmc SDS + 100 ppm chitosan oligomer				
1	1	1	133	43	1.6	2	90	5.5
	1	2	190	37	1.7	2	63	3.9
	2	3	174	46	2.4	2	77	5.3
	2	4	152	46	2	2	73	4.5
	2	5	281	47	5.6	2	89	5.9
	3	6	297	43	3.1	2	49	3.4
	3	7	306	42	3.3	2	52	3.9
2	1	1	393	24	1.1	1	32	1.1
	1	2	347	25	1.4	1	25	1.2
	2	3	354	23	0.9	1	17	1.1
	2	4	279	26	0.9	1	16	1.2
	3	5	299	27	1.1	1	18	1.3
	3	6	360	29	1.5	1	27	2.3

Table S4 Particle analysis for the 50:50 PS:18-MEA thiol surfaces in the presence of chitosan, SDS or SDS/chitosan complex. Images are numbered per sample and adsorbing species. More images were normally collected for each region of interest (ROI). Total projected area and median height were then averaged to obtain the values in Table S1

Sample	ROI	Image number	Number of grains	Mean grain size (nm)	Total projected area (%)	Minimum height (nm)	Maximum height (nm)	Median height (nm)
				100 ppm chitosan oligomer				
1	1	1	163	32	1	1	16	1.2
	1	2	219	50	6.7	1	44	2.2
	1	3	253	30	1.3	1	24	1.2
	2	4	77	67	3.4	1.5	44	3.6
	3	5	14	66	0.5	1.5	29	4.6
	3	6	47	63	1	1.5	7.4	2
	4	7	448	72	17	1	14	1.8
2	1	1	2824	31	13	1	16	1.6
	1	2	1726	27	6.3	1.5	18	2.1
	2	3	2746	31	15	1	13	1.7
	3	4	952	36	7.9	1	21	1.6
	3	5	1220	40	15	1	24	2.4
				2 cmc SDS				
1	1	1	325	35	2.6	1	13	1.4
	1	2	329	37	3.2	1	10	1.6
	2	3	301	30	1.6	1	20	1.2
	2	4	267	31	1.4	1	24	1.3
	3	5	157	43	2.6	2	97	7.8
	3	6	176	29	0.9	1	21	1.2
	4	7	205	28	0.9	1	16	1.2
2	1	1	711	40	8.8	1	49	3
	1	2	1169	35	10.5	1	31	2.2
	2	3	1518	28	6.3	1	24	1.5
	3	4	1539	31	8.4	1	26	1.8
				20 cmc SDS + 100 ppm chitosan oligomer				
1	1	1	249	27	1	1	7.7	1.2
	1	2	131	41	2	1.5	56	4.8
	2	3	500	29	2.2	1	6.5	1.3
	2	4	383	33	2.1	1	14	1.5
	3	5	368	36	2.9	1	12	2
	3	6	249	46	3.9	1.5	109	5.4
	3	7	284	29	1.3	1	23	1.4
2	1	1	1386	27	4.8	1	13.3	1.4
	1	2	1152	29	6..2	1.2	21	2
	1	3	1575	27	5.5	1	9.4	1.4
	2	4	926	29	7.2	1.5	60	4.2
	3	5	1391	33	9.1	1.5	67	2.6
	3	6	997	30	5.2	1.2	18	2.2

Higher resolution AFM images (size of 2 µm) were also acquired in some cases. Examples are in Fig. S11 and S12, while Fig. S13, S14 and S15 show examples of extracted profiles for the three thiol surfaces.

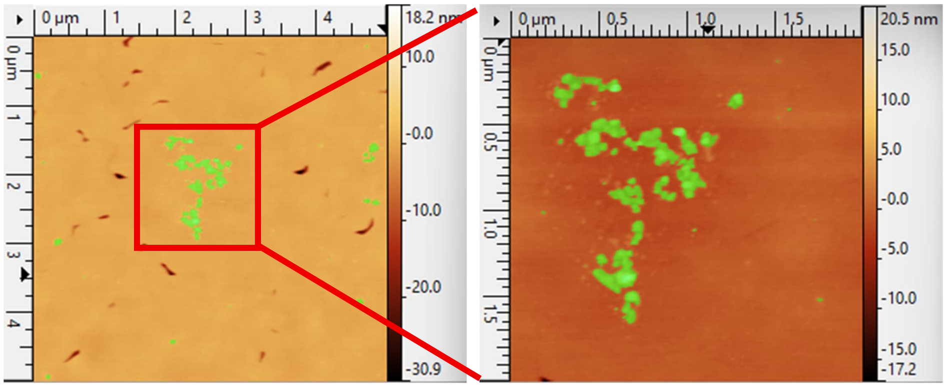

Fig. S11 AFM image (sample 1, image number 1) of chitosan adsorbed on the 18-MEA thiol surface. While coverage was mostly low, in this case a cluster of particles was visible, so a higher-resolution image was acquired.

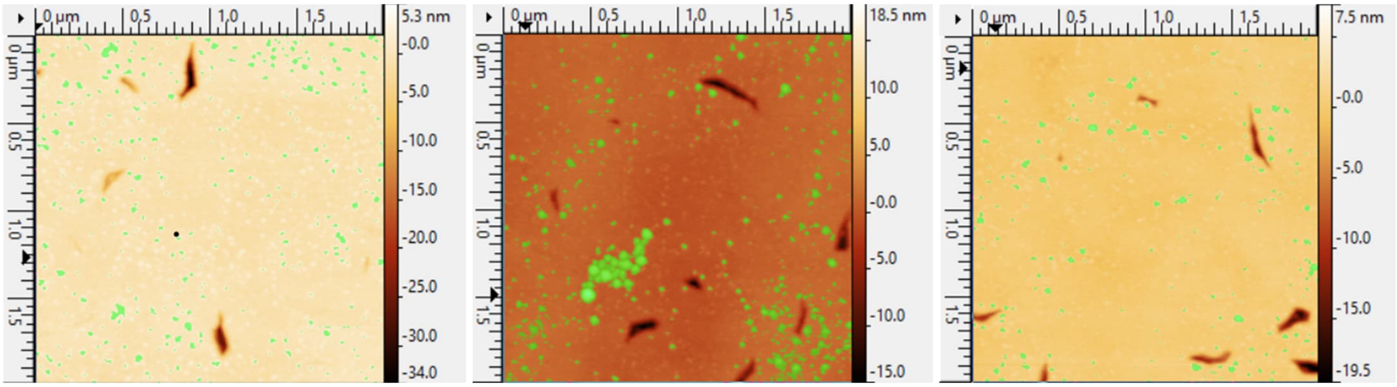

(a) (b) (c)

Fig. S12 Higher resolution AFM images of: a) chitosan, b) SDS and c) SDS/chitosan complex adsorbed on the 50:50 PS:18-MEA thiol surface.

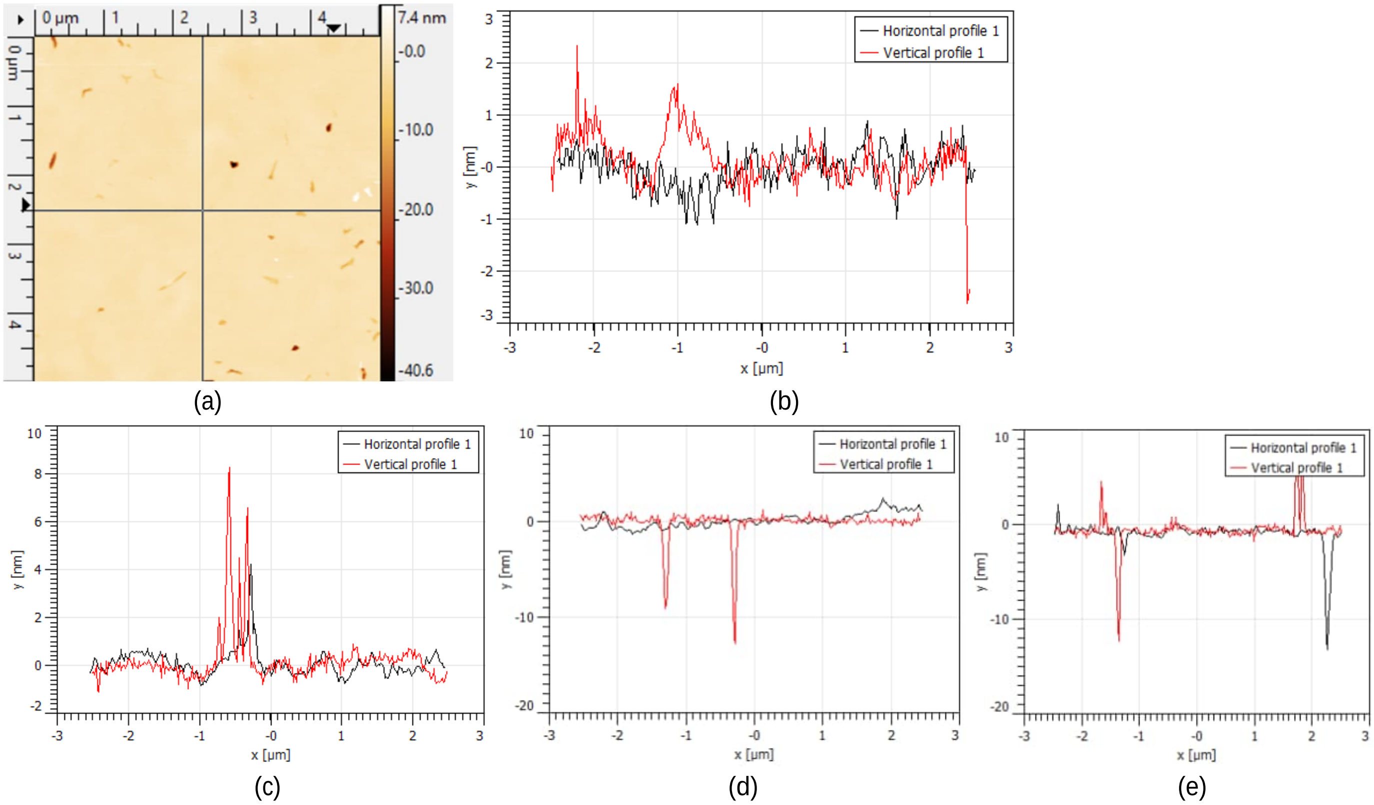

Fig. S13 a) AFM image of the 18-MEA thiol surface in 100 mM NaCl, with b) corresponding extracted profiles along the lines crossing at the centre in a), plus examples of extracted profiles from the AFM images of: c) chitosan, d) SDS and e) chitosan/SDS complex on the 18-MEA thiol surface. The corresponding images are those shown in Figures S11, S9(a) and S10(a). Profiles are extracted from lines crossing in the middle of the image.

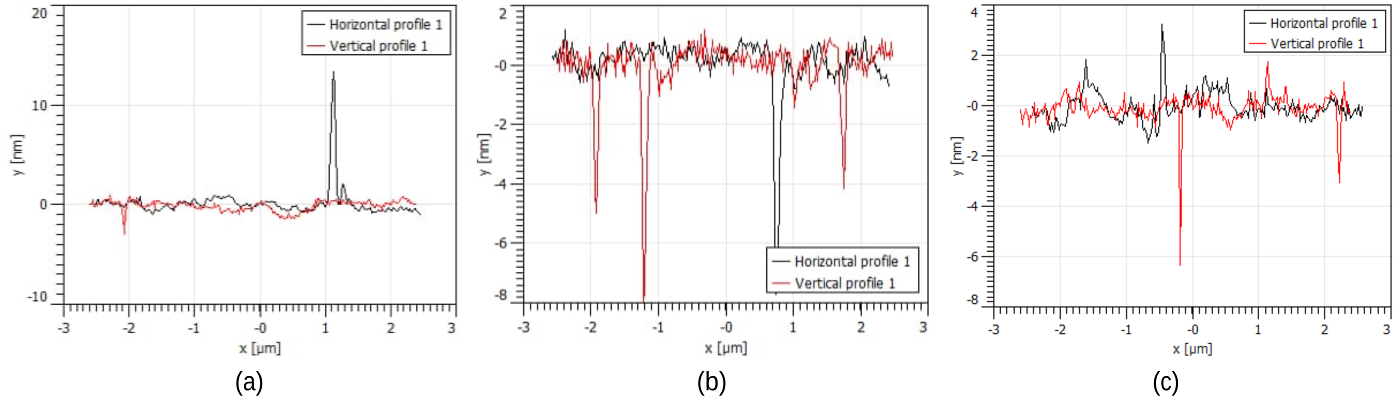

Fig. S14 Examples of extracted profiles from the AFM images of: a) chitosan, b) SDS and c) chitosan/SDS complex on the 50:50 PS:18-MEA thiol surface. The corresponding images are those shown in Figures 12(b), S9(b) and S10(b). Profiles are extracted from lines crossing in the middle of the image.

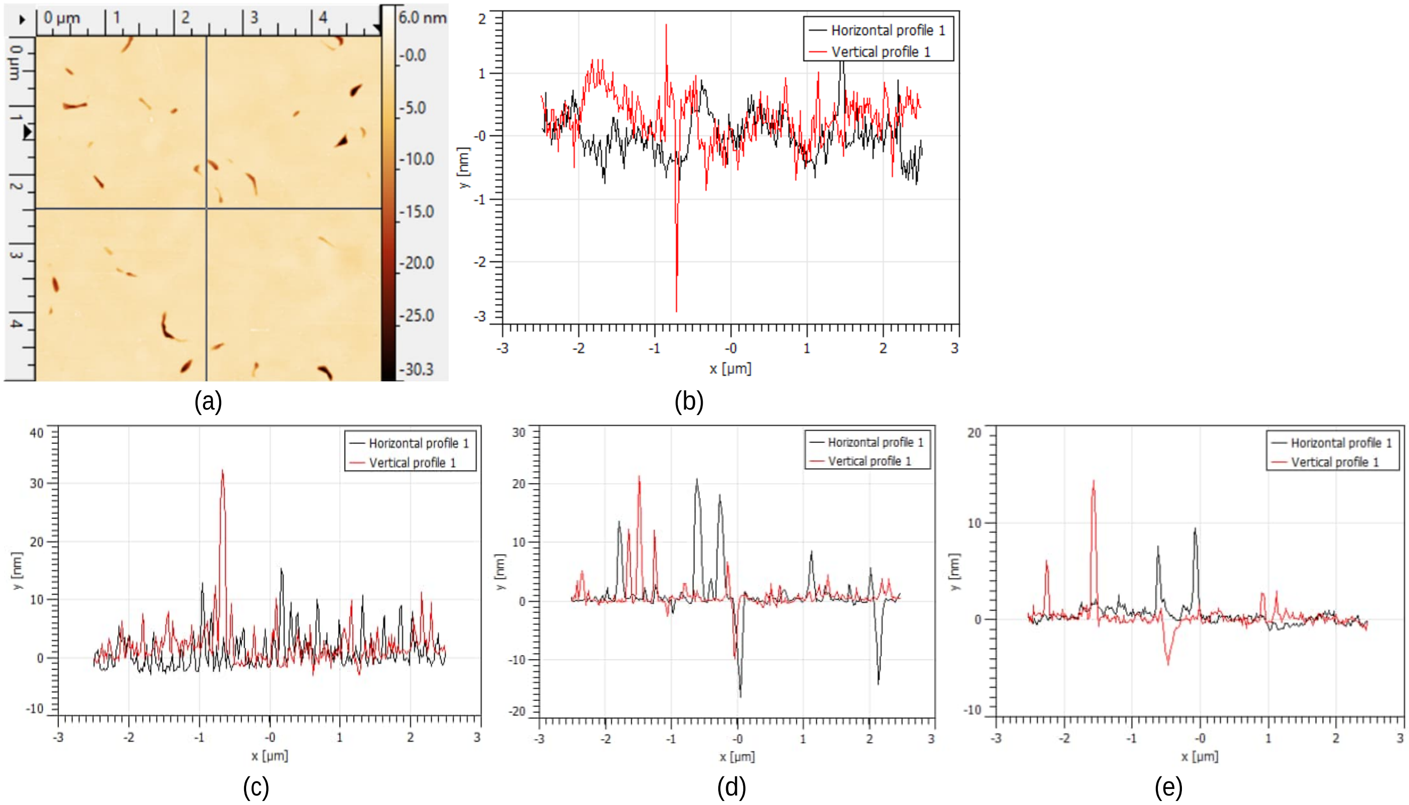

Fig. S15 a) AFM image of the PS surface in 100 mM NaCl, with b) corresponding extracted profiles along the lines crossing at the centre in a), plus examples of extracted profiles from the AFM images of: c) chitosan, d) SDS and e) chitosan/SDS complex on the 18-MEA thiol surface. The corresponding images are those shown in Figures 12(c), S9(c) and S10(c). Profiles are extracted from lines crossing in the middle of the image.

S5 Fitted Neutron Reflectometry (NR) data

Below the fitted reflectometry data are shown. Unless otherwise stated, the graph on the left shows the experimental curve (dots) for the NR measurement of the specified system (in the presence of 100 mM NaCl in GCMW) with the fitted model (red line), while the graph on the right shows the corresponding SLD profile, with shadowed areas representing the range of possible solutions.

S5.1 18-MEA thiol surface

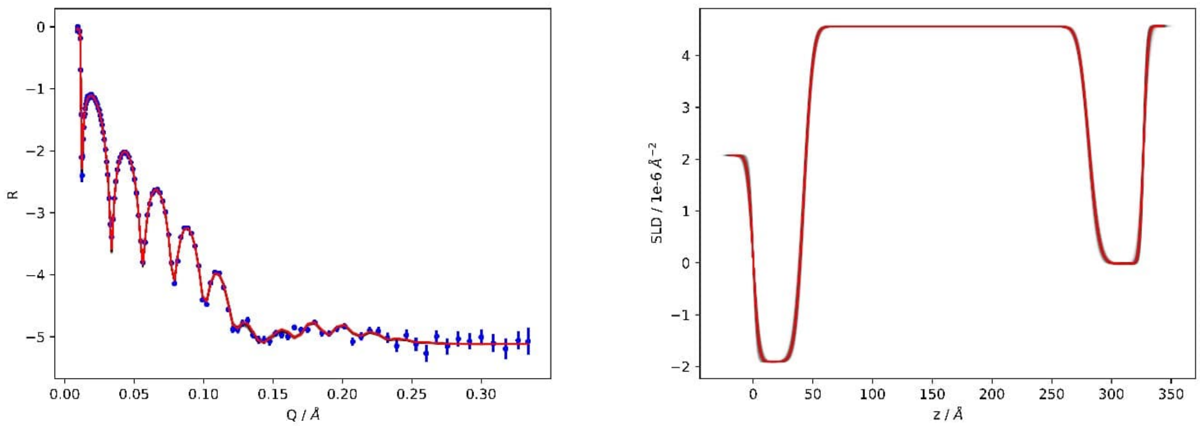

Fig. S16 18-MEA thiol

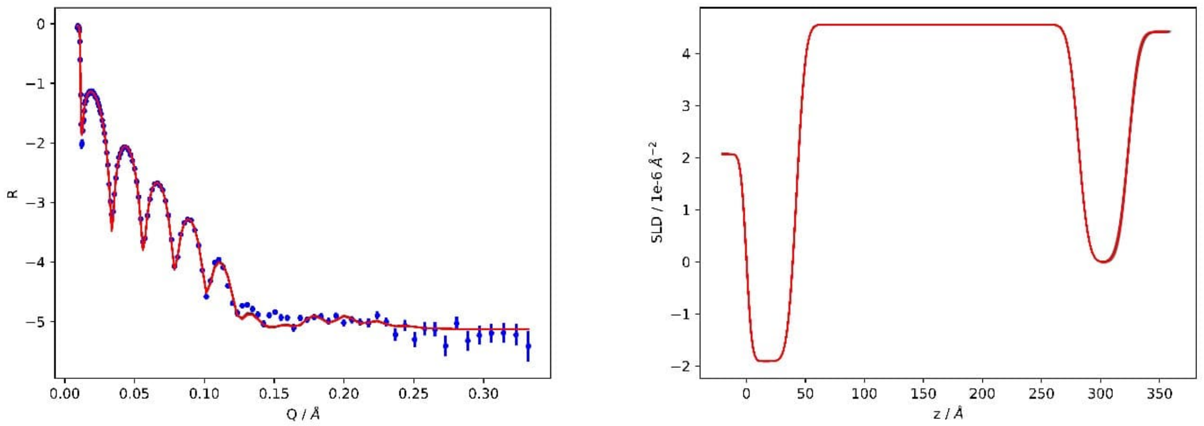

Fig. S17 18-MEA thiol and 0.1 cmc d25-SDS

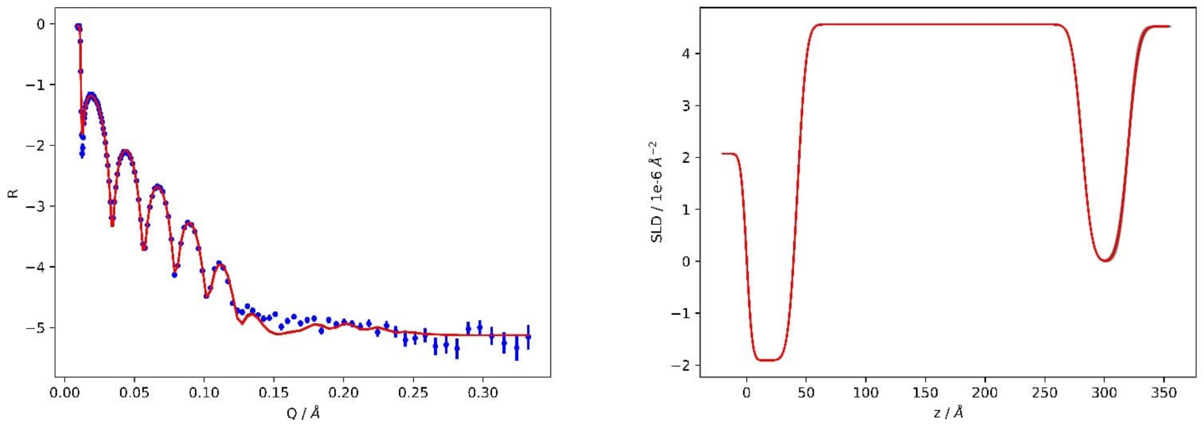

Fig. S18 18-MEA thiol and 0.5 cmc d25-SDS

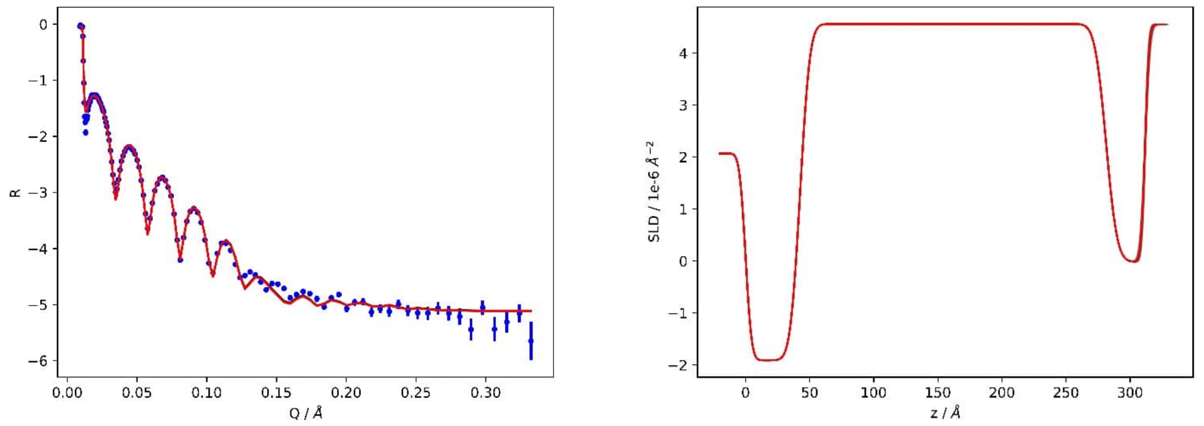

Fig. S19 18-MEA thiol and 2 cmc d25-SDS

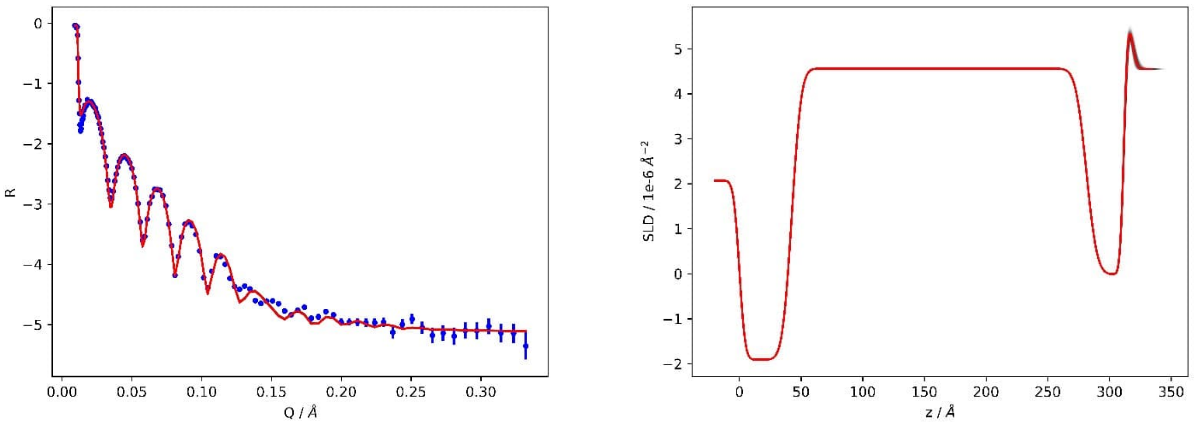

Fig. S20 18-MEA thiol and 20 cmc d25-SDS

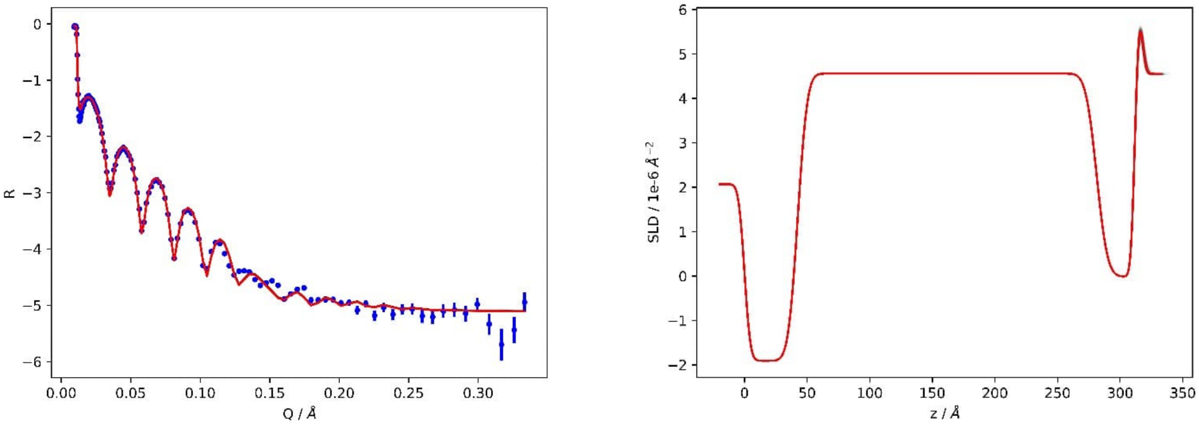

Fig. S21 18 MEA thiol and 20 cmc d25-SDS + 100 ppm chitosan oligomer

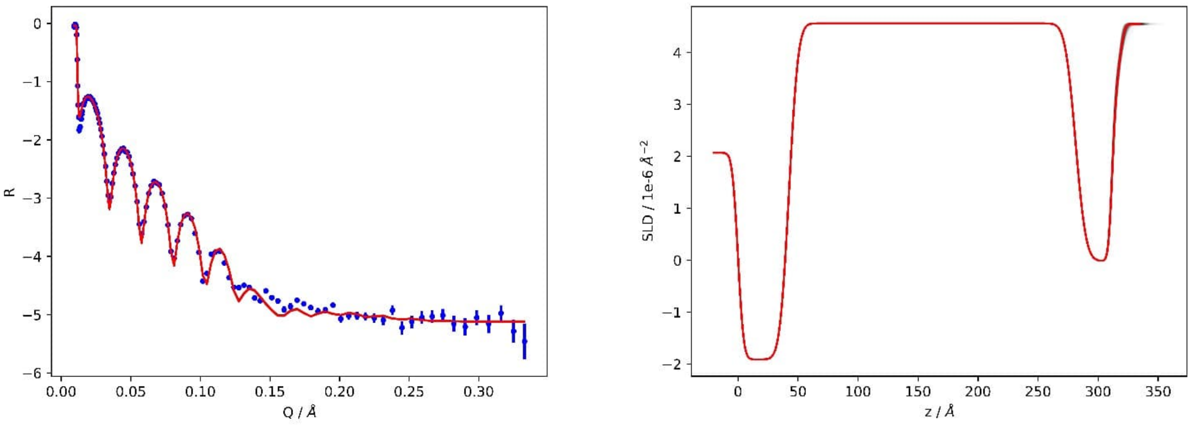

Fig. S22 18-MEA thiol after rinse of d25-SDS + chitosan

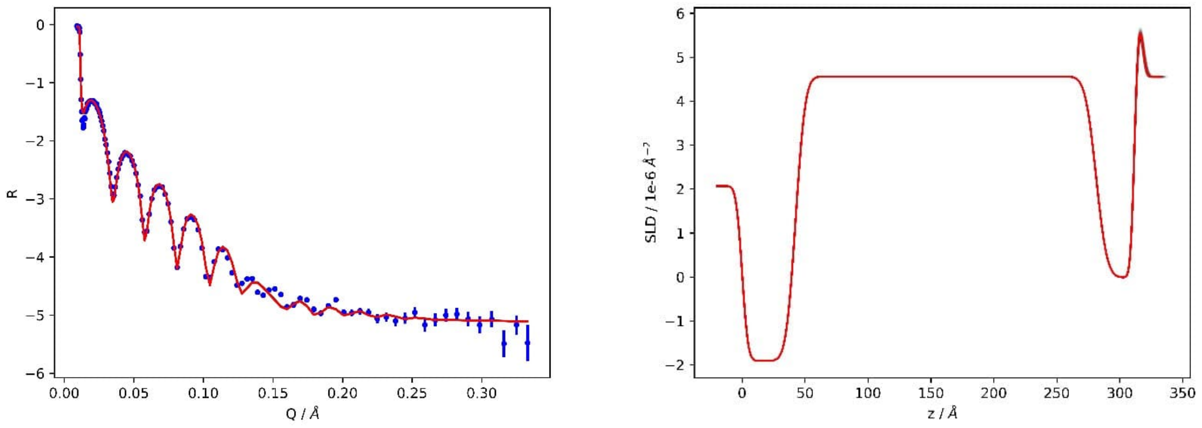

Fig. S23 18-MEA thiol and 20 cmc d25-SDS (after rinse of the previous complex)

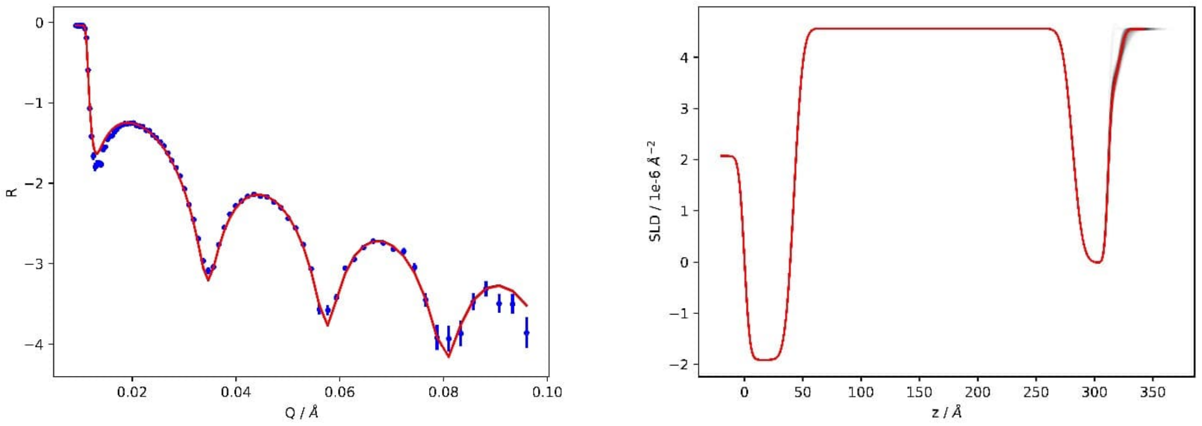

Fig. S24 18 MEA thiol after a final rinse

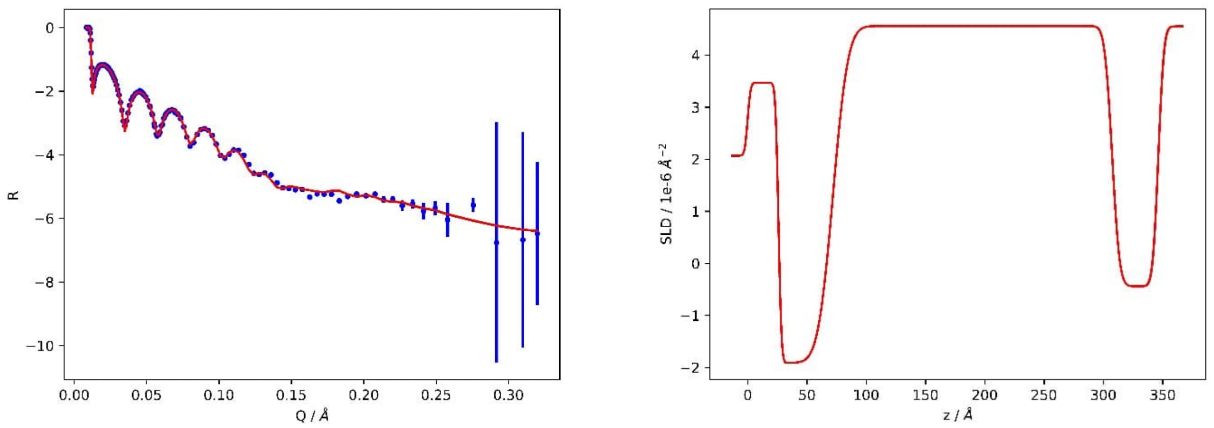

Fig. S25 18-MEA thiol

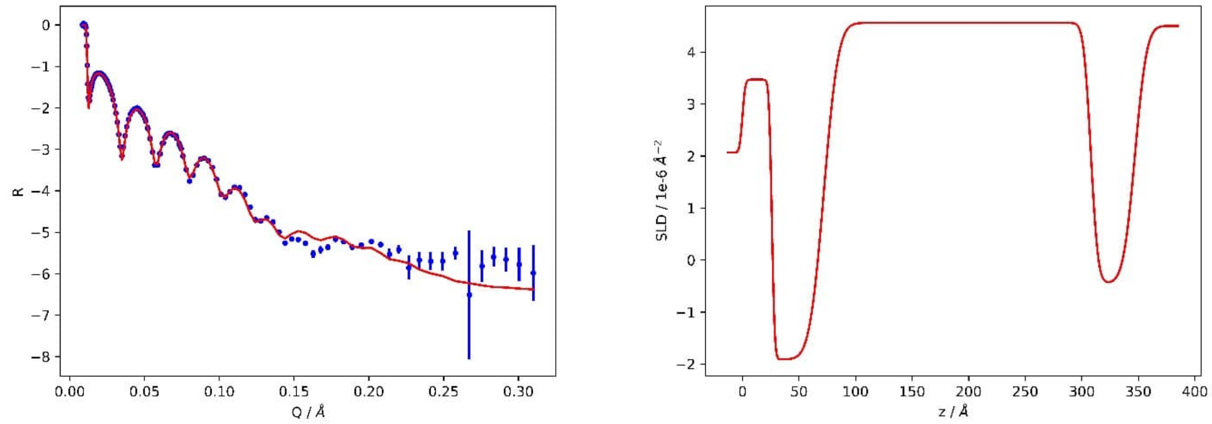

Fig. S26 18-MEA thiol and 0.1 cmc d42-CTAC

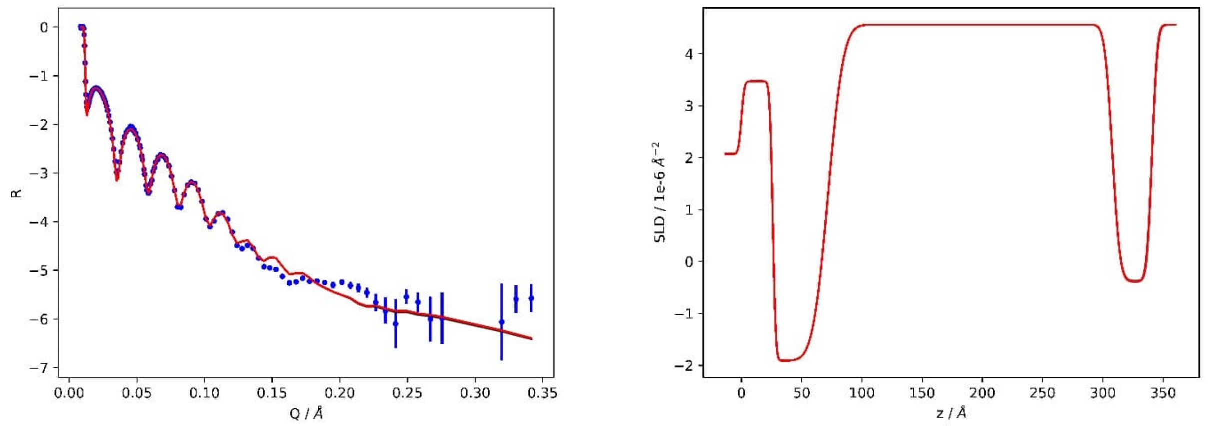

Fig. S27 18 MEA thiol and 2 cmc d42-CTAC

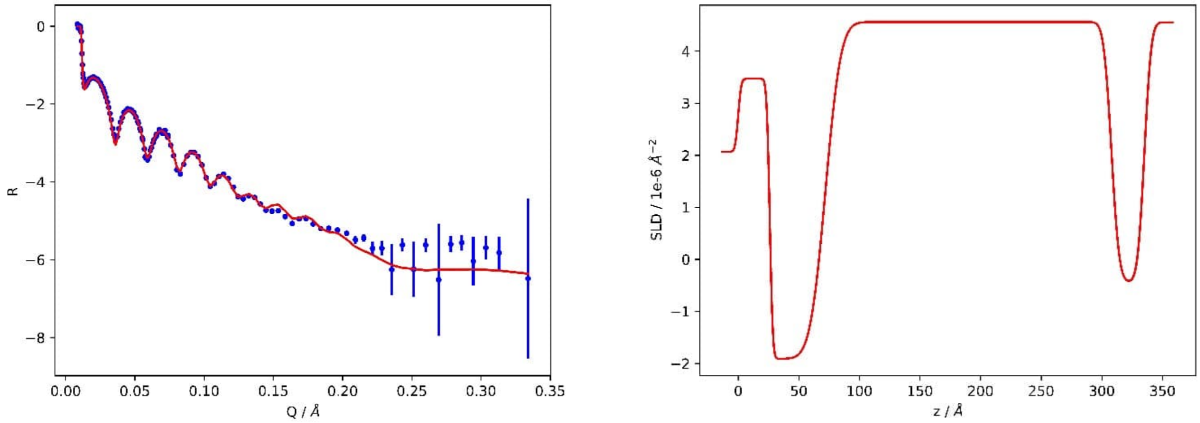

Fig. S28 18-MEA thiol and 20 cmc d42-CTAC

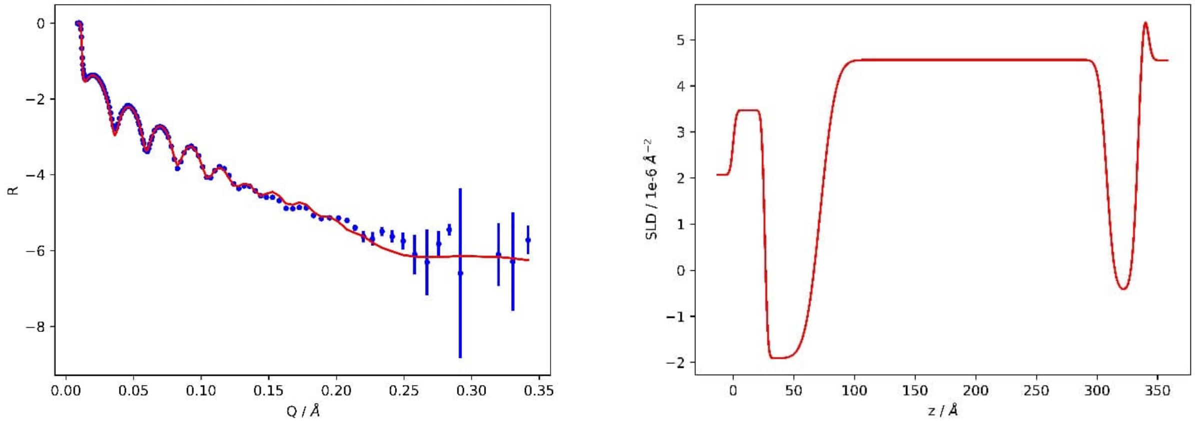

Fig. S29 18-MEA thiol and 20 cmc d42-CTAC + 100 ppm chitosan oligomer

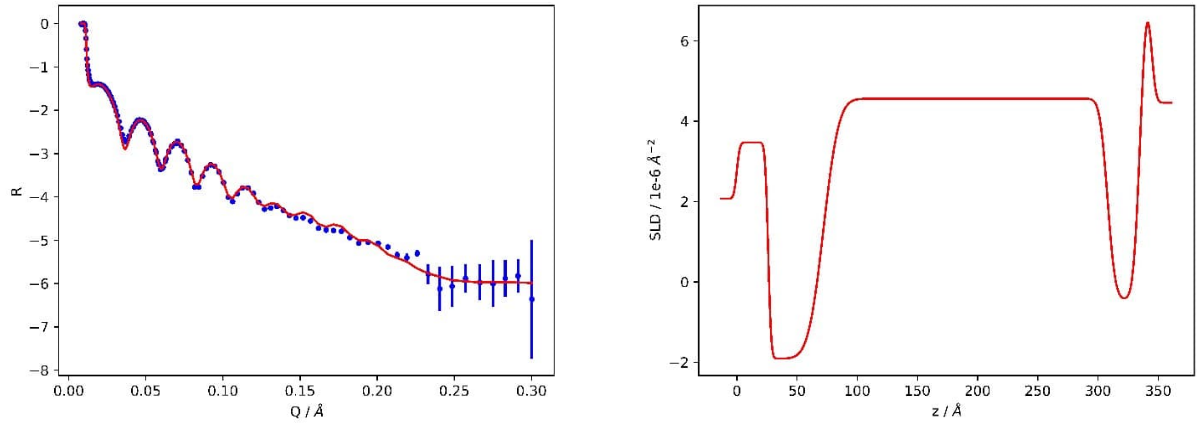

Fig. S30 18 MEA thiol and 62.5 cmc d42-CTAC + 12.5 ppm chitosan oligomer

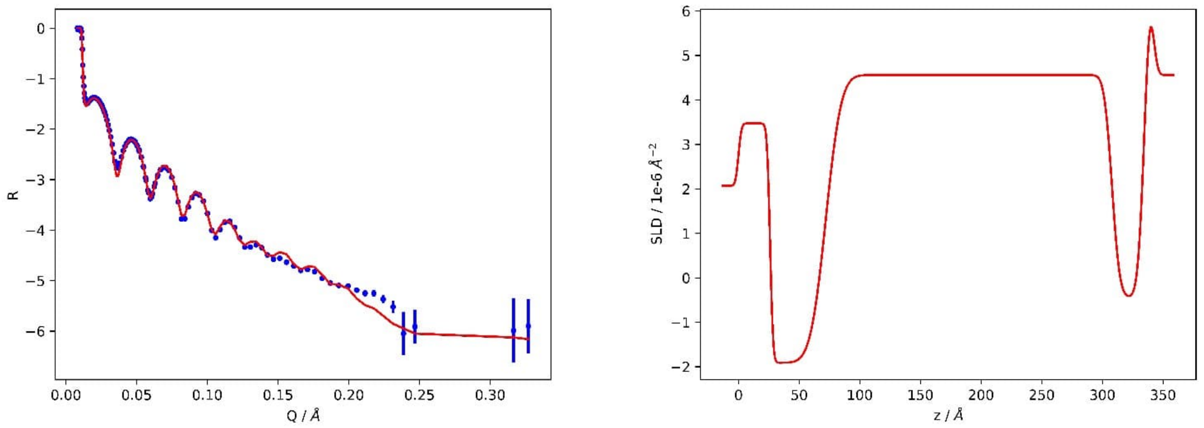

Fig. S31 18-MEA thiol after rinse of d42-CTAC + chitosan oligomer

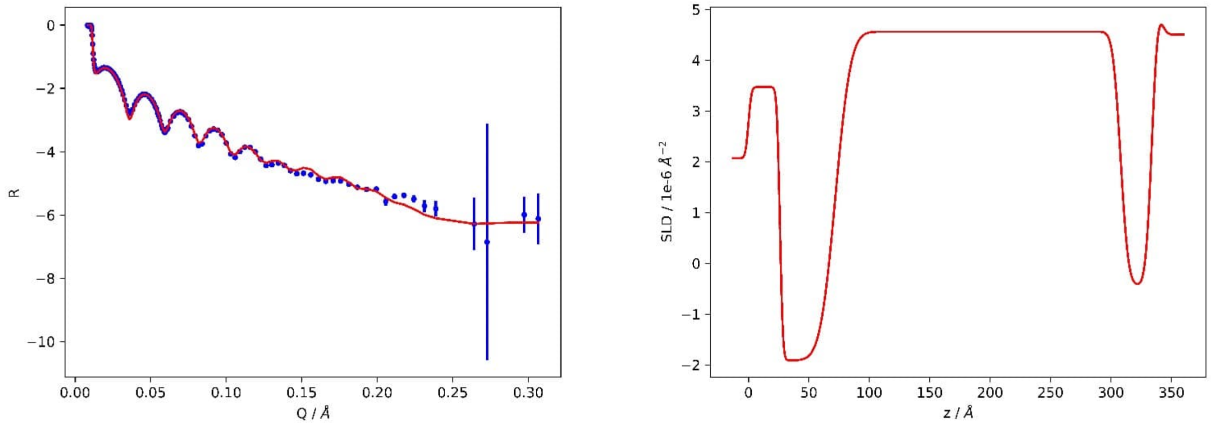

Fig. S32 18-MEA thiol and 100 ppm chitosan oligomer

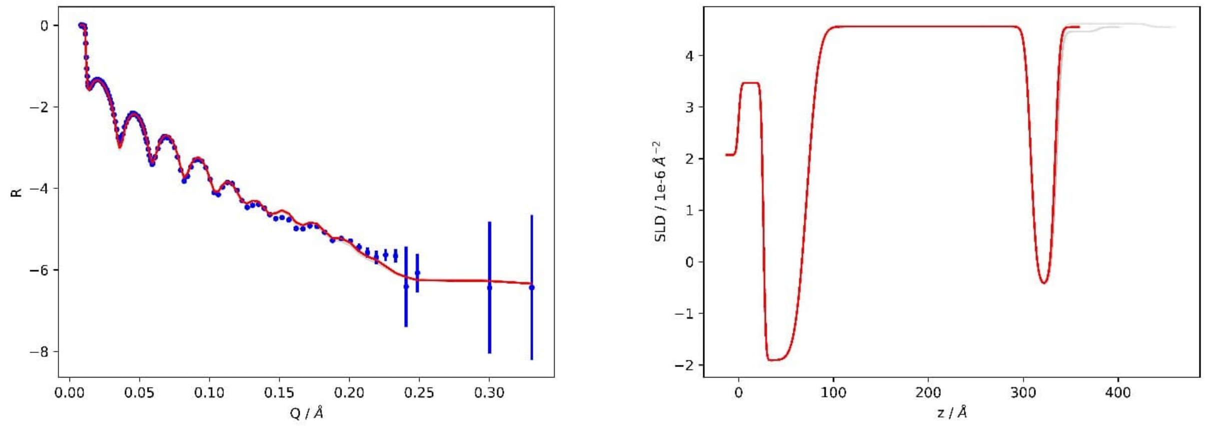

Fig. S33 18 MEA thiol after rinse of chitosan oligomer

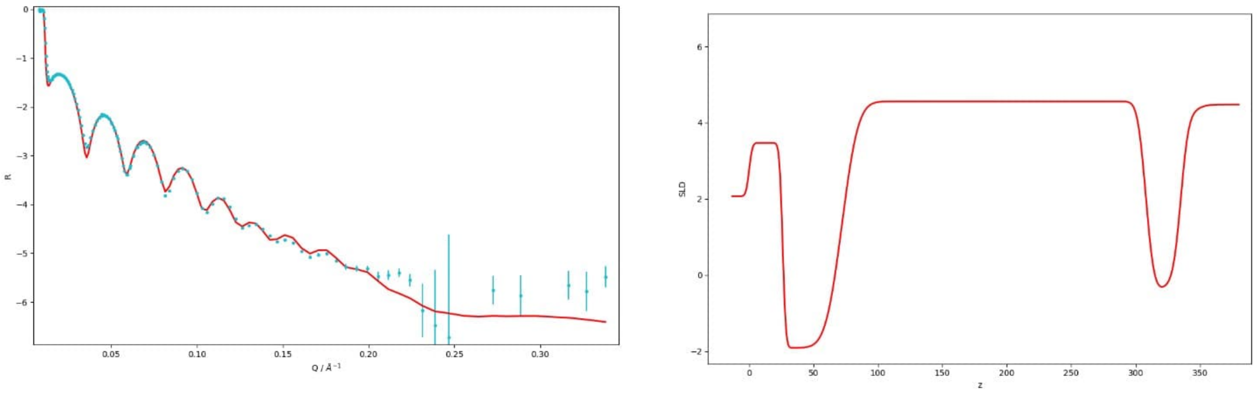

Fig. S34 18-MEA thiol and 100 ppm chitosan polymer

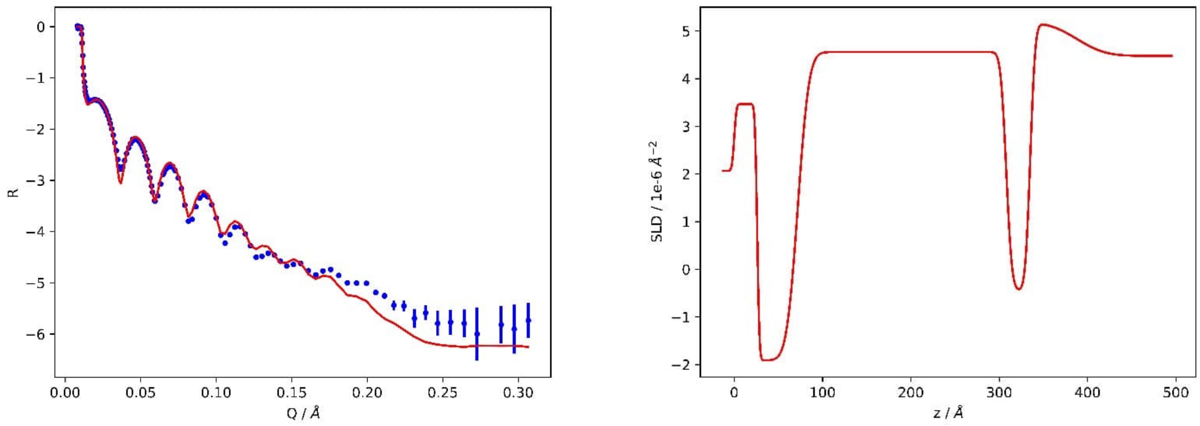

Fig. S35 18-MEA thiol and 20 cmc d25-SDS (after chitosan polymer)

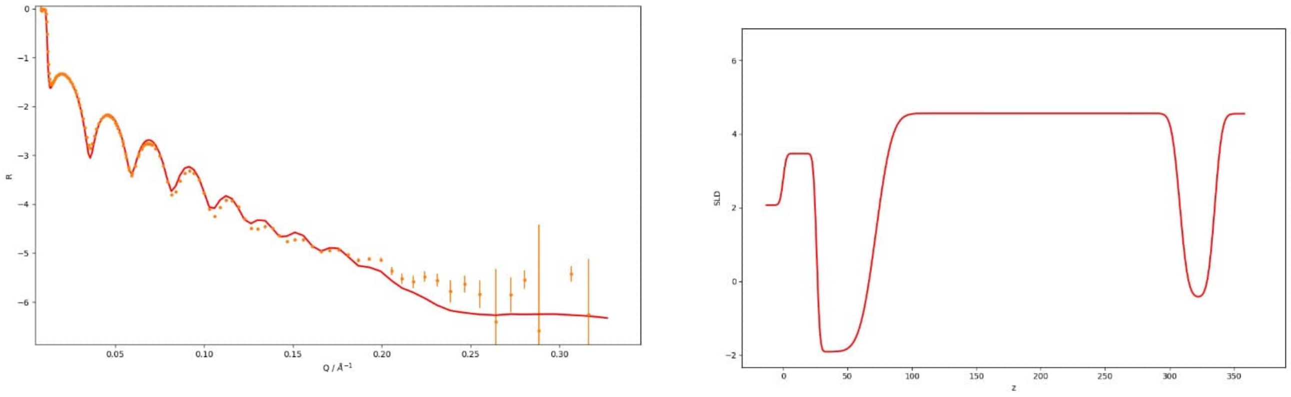

Fig. S36 18-MEA thiol after rinse of d25-SDS

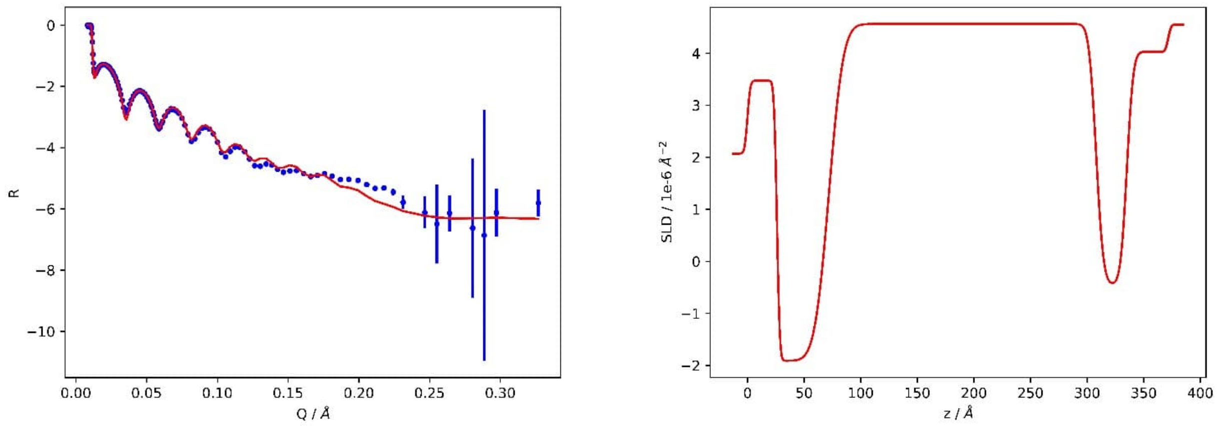

Fig. S37 18-MEA thiol and 100 ppm pDADMAC

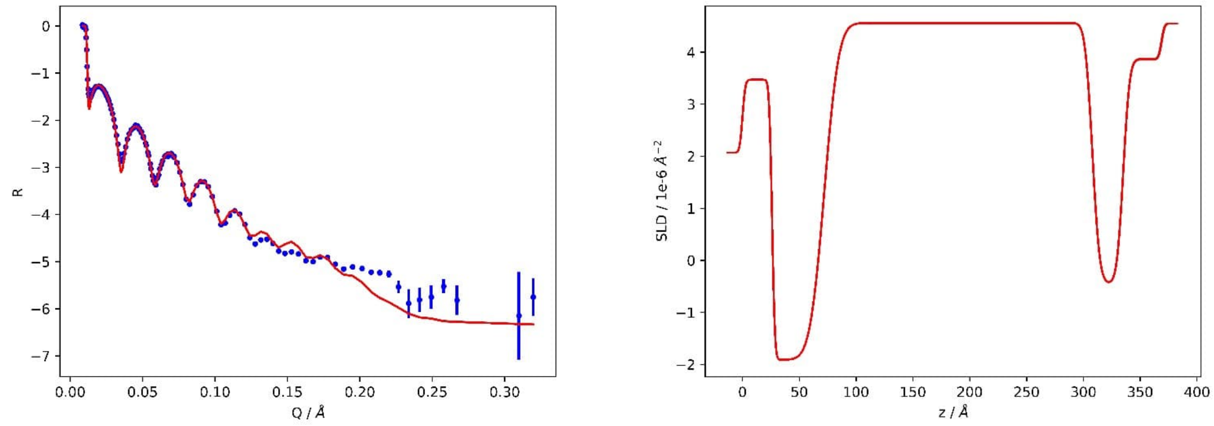

Fig. S38 18-MEA thiol after rinse of pDADMAC

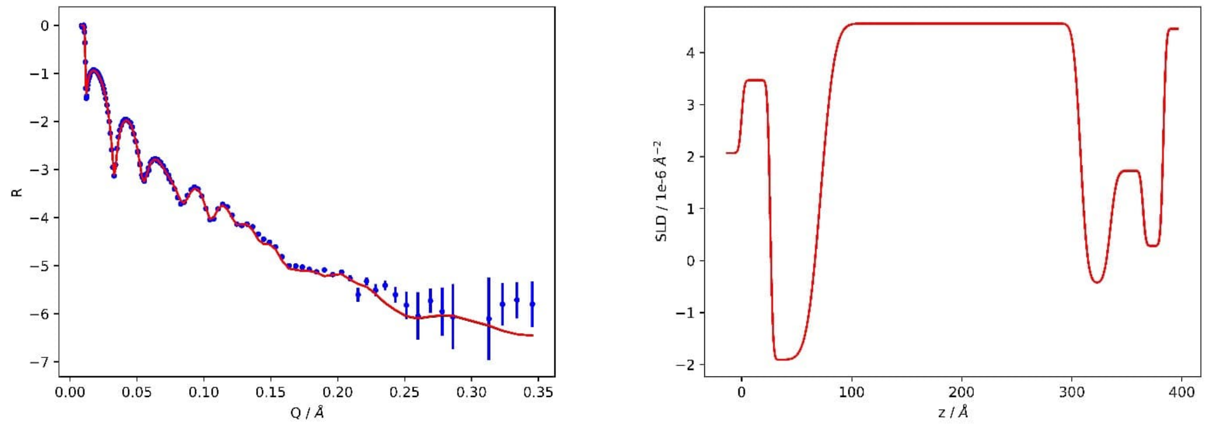

Fig. S39 18-MEA thiol and 0.5 cmc h-SDS

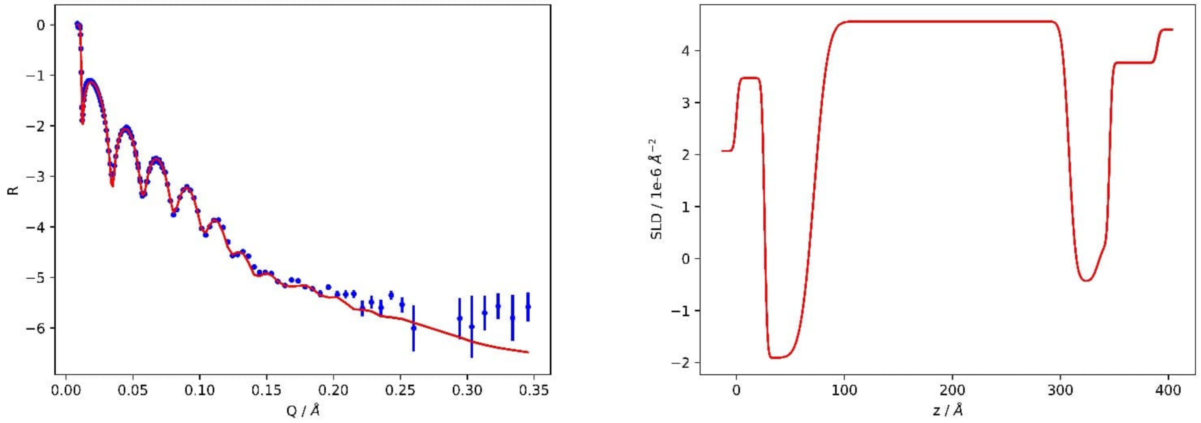

Fig. S40 18-MEA thiol and 20 cmc h-SDS

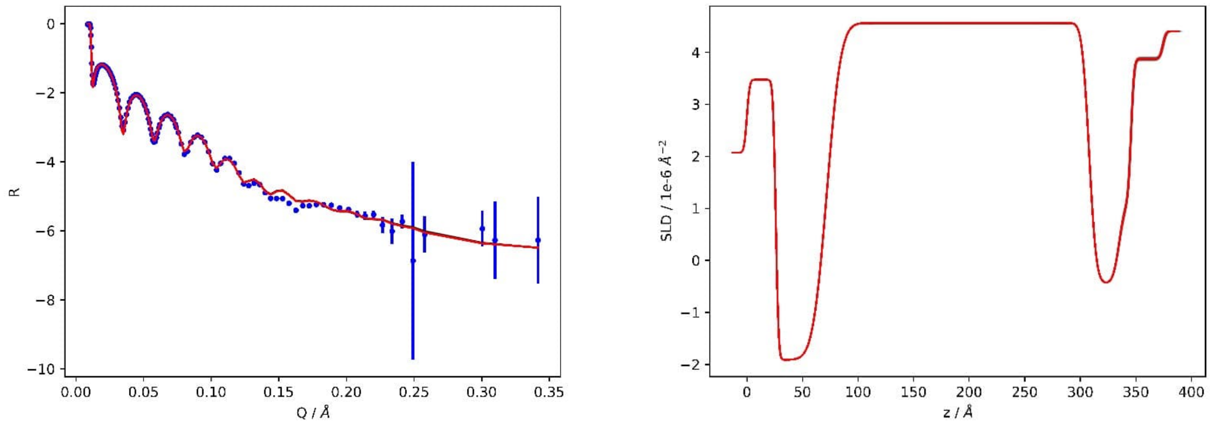

Fig. S41 18-MEA thiol after final rinse

Table S5 Bulk SLD values and fitted layer thickness, roughness and SLD values for adsorbed layers on 18-MEA thiol surface (sequence NR1). Numbers in parenthesis indicate the error on the parameter value, equal to 2.5σ. The values in italics were manually adjusted. Up to 2 cmc d25-SDS, the thiol layer was fitted, after that the fit refers to an additional slab

	bulk SLD ($*10^{-6}$Å^{-2})	SLD ($*10^{-6}$Å^{-2})	Thickness (Å)	Roughness (Å)
Thiol layer	4.570 (0.008)	*-0.015*	46 (1)	3 (2)
0.1 cmc d25-SDS	4.421 (0.008)	*-0.015*	42.4 (0.4)	7 (1)
0.5 cmc d25-SDS	4.423 (0.009)	*-0.015*	38.9 (0.4)	7 (1)
2 cmc d25-SDS	*4.55*	*-0.015*	30.5 (0.3)	3 (1)
20 cmc d25-SDS	*4.55*	5.9 (0.6)	7 (3)	3 (2)
d25-SDS/oligomer	*4.55*	6.1 (0.2)	6 (1)	2.3 (0.9)
Rinse	*4.55*	3.3 (0.3)	7 (2)	3 (2)
20 cmc d25-SDS	*4.55*	6.1 (0.2)	6 (1)	2 (1)
Rinse	*4.55*	3.4 (0.5)	10 (5)	4 (4)

Table S6 Bulk SLD values and fitted layer thickness, roughness and SLD values for adsorbed layers on 18-MEA thiol surface (sequence NR2). Numbers in parenthesis indicate the error on the parameter value, equal to 2.5σ. The values in italics were manually adjusted. Up to 20 cmc d42-CTAC, the second slab of the thiol layer (18-MEA chains) was fitted, after that the fit refers to an additional slab

	bulk SLD ($*10^{-6}$Å^{-2})	SLD ($*10^{-6}$Å^{-2})	Thickness (Å)	Roughness (Å)
Thiol layer	4.554 (0.002)	0.8 (0.2)	2.0 (0.1)	*5*
		-0.440 (0.001)	36.9 (0.1)	3.8 (0.2)
0.1 cmc d42-CTAC	4.502 (0.004)	-0.440 (0.001)	37.53 (0.08)	8.2 (0.1)
2 cmc d42-CTAC	4.556 (0.002)	-0.38 (0.02)	31.4 (0.1)	3.5 (0.4)
20 cmc d42-CTAC	*4.56*	-0.438 (0.007)	25.64 (0.08)	4.4 (0.1)
d42-CTAC/oligomer 1	*4.56*	7.69 (0.03)	5.8 (0.1)	3.02 (0.07)
d42-CTAC/oligomer 2	*4.46*	7.700 (0.001)	9.03 (0.06)	3.002 (0.007)
Rinse	*4.56*	7.699 (0.002)	6.45 (0.07)	3.002 (0.006)
Chitosan oligomer	*4.5*	7.699 (0.004)	3.78 (0.07)	4.001 (0.003)
Rinse	*4.55*	7.70 (0.01)	2.4 (0.1)	4.001 (0.004)
20 cmc d25-SDS	*4.48*	5.19 (0.01)	53 (1)	25.3 (0.5)
pDADMAC	*4.55*	4.025 (0.008)	36.5 (0.4)	2.02 (0.05)
Rinse	*4.55*	3.86 (0.01)	33.8 (0.4)	2.1 (0.2)
0.5 cmc h-SDS	*4.46*	1.73 (0.01)	29.6 (0.2)	2.02 (0.06)
		0.281 (0.003)	18.5 (0.2)	2.01 (0.03)
20 cmc h-SDS	*4.4*	0.280 (0.001)	10.5 (0.1)	2.00 (0.02)
		3.76 (0.01)	44.0 (0.5)	2.1 (0.2)
Rinse	*4.48*	1.2 (0.4)	11 (1)	*2*
		3.9 (0.3)	31 (7)	*2*

S5.2 EA thiol surface

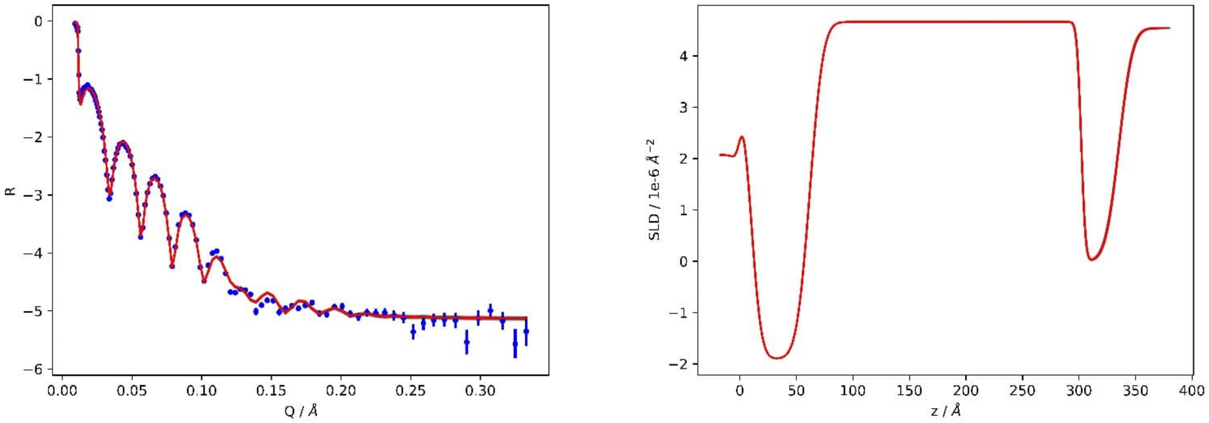

Fig. S42 EA thiol

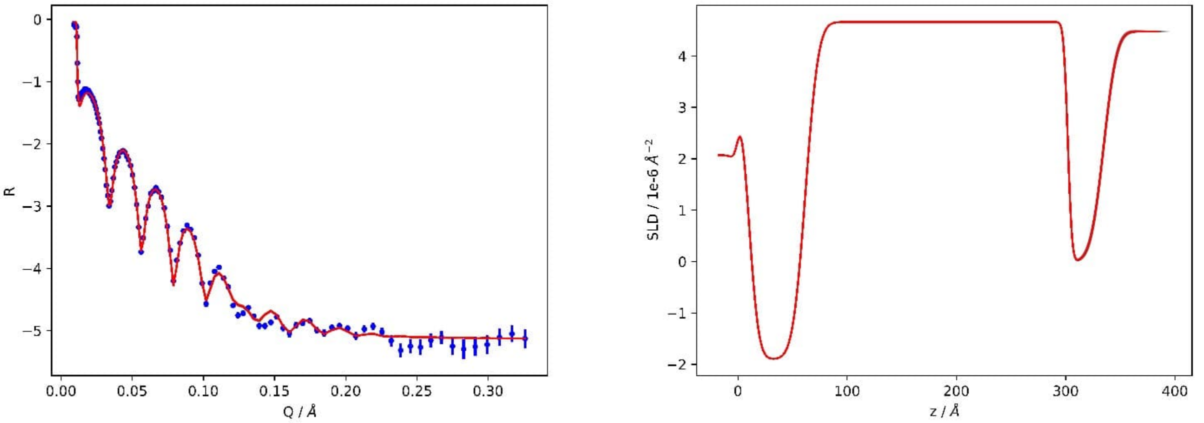

Fig. S43 EA thiol and 0.1 cmc d25-SDS

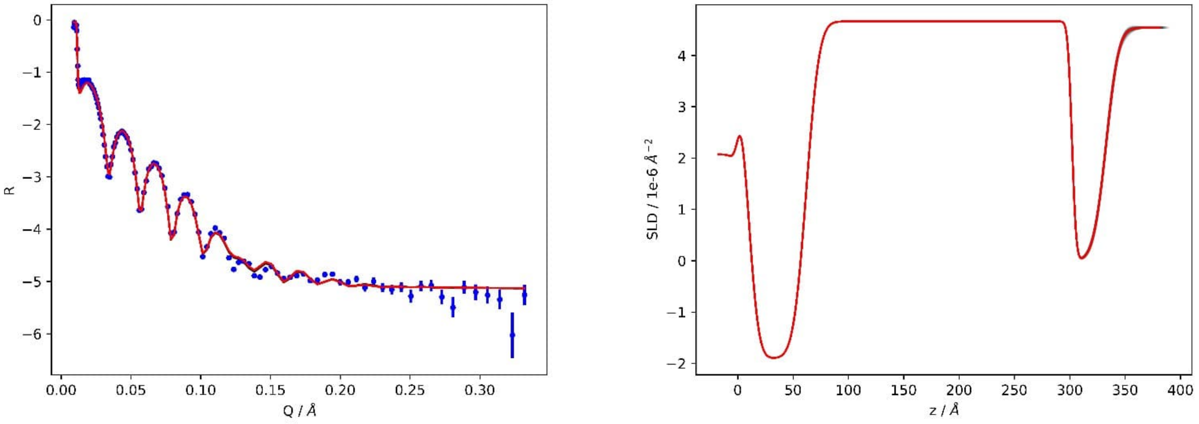

Fig. S44 EA thiol and 0.5 cmc d25-SDS

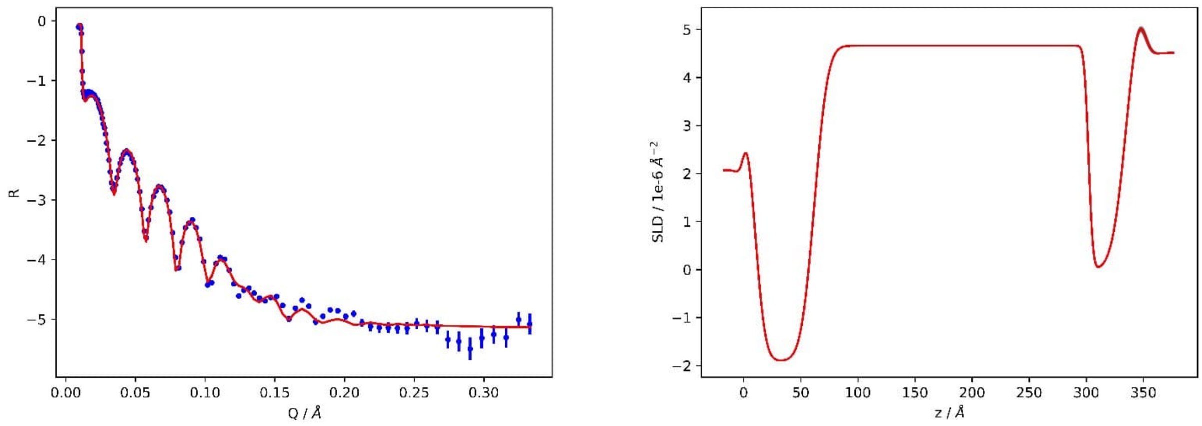

Fig. S45 EA thiol and 2 cmc d25-SDS

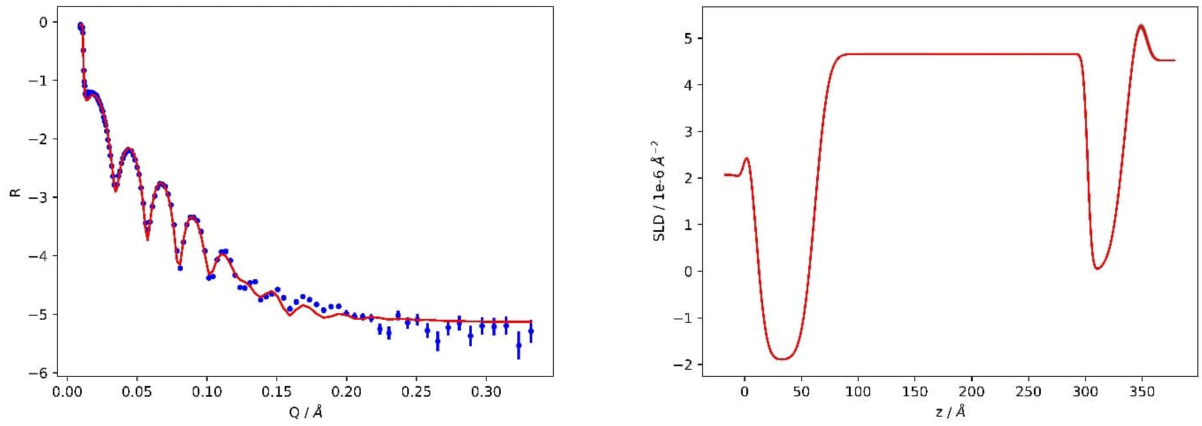

Fig. S46 EA thiol and 20 cmc d25-SDS

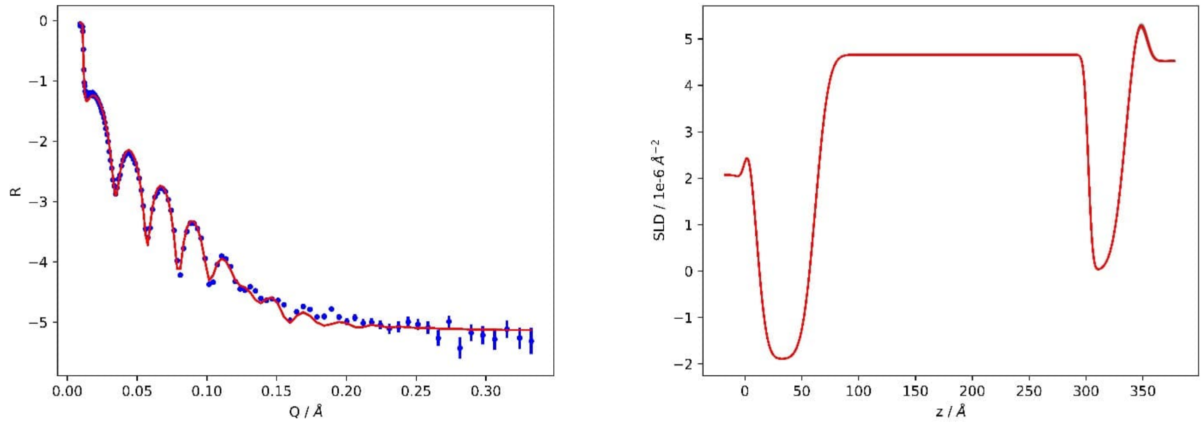

Fig. S47 EA thiol and 20 cmc d25-SDS + 100 ppm chitosan oligomer

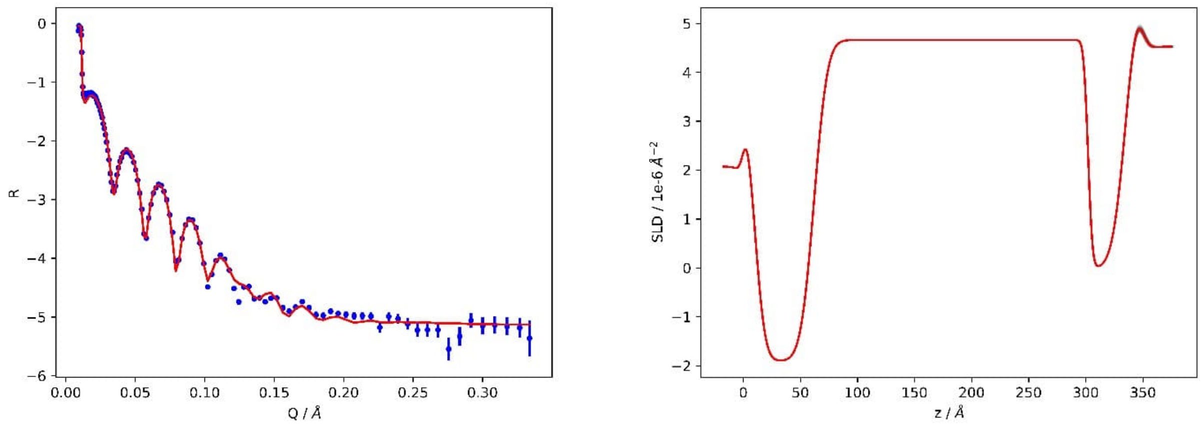

Fig. S48 EA thiol after rinse of d25-SDS + chitosan oligomer

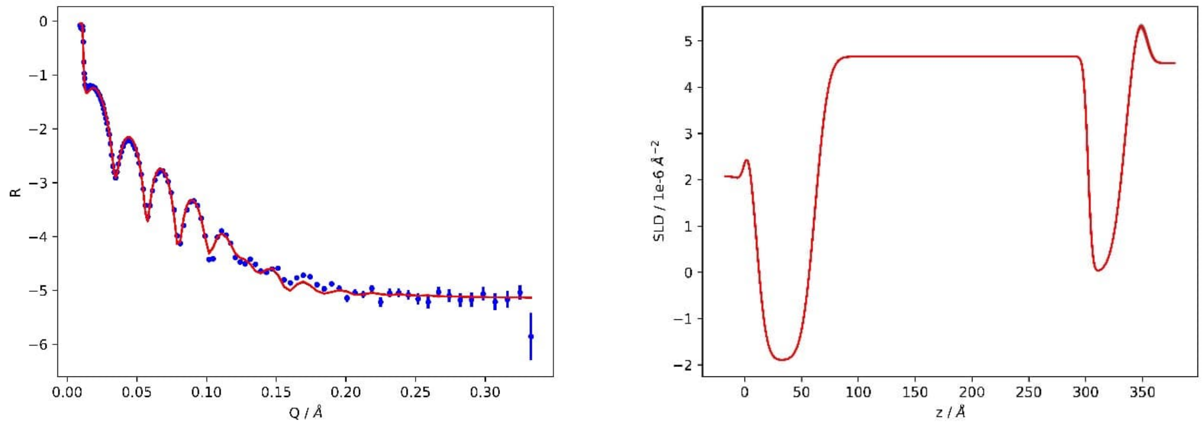

Fig. S49 EA thiol and 20 cmc d25-SDS after rinse

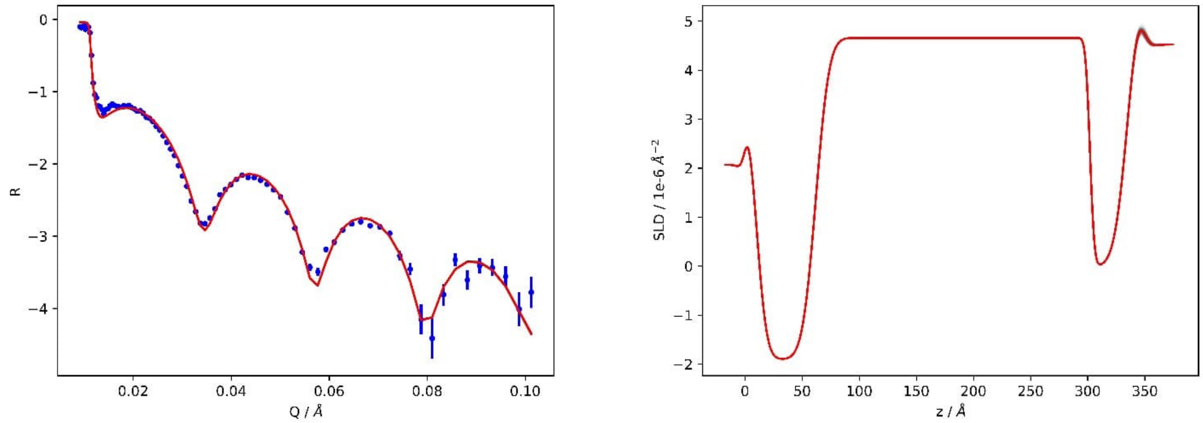

Fig. S50 EA thiol after final rinse

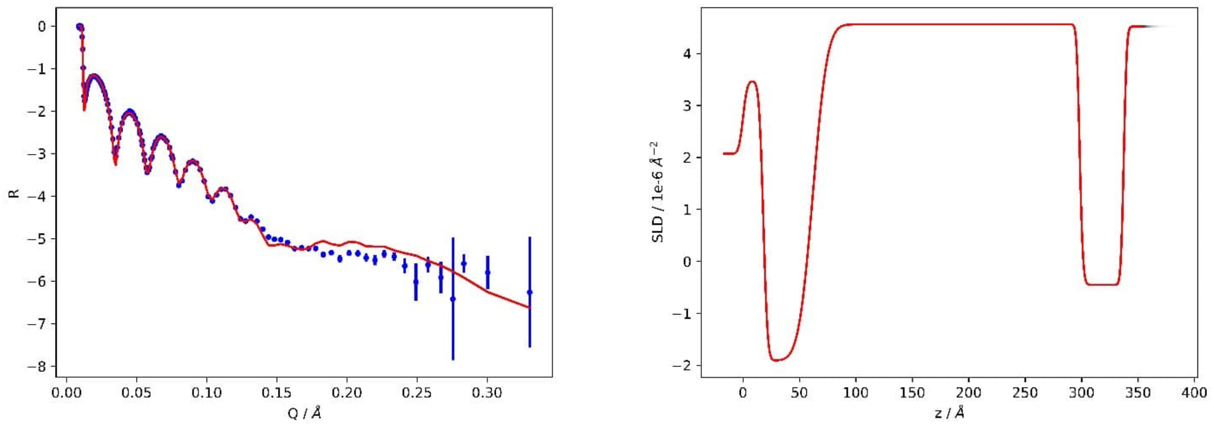

Fig. S51 EA thiol

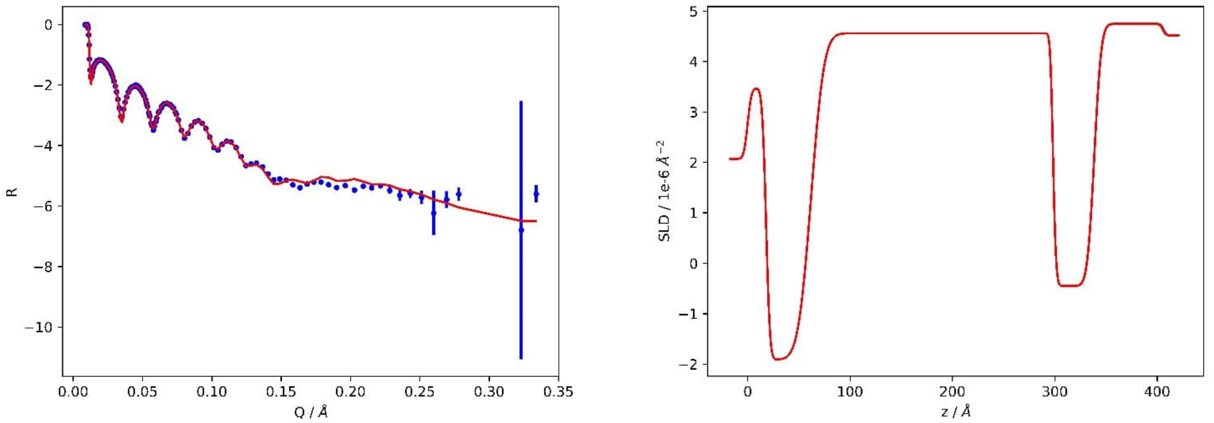

Fig. S52 EA thiol and 0.1 cmc d42-CTAC

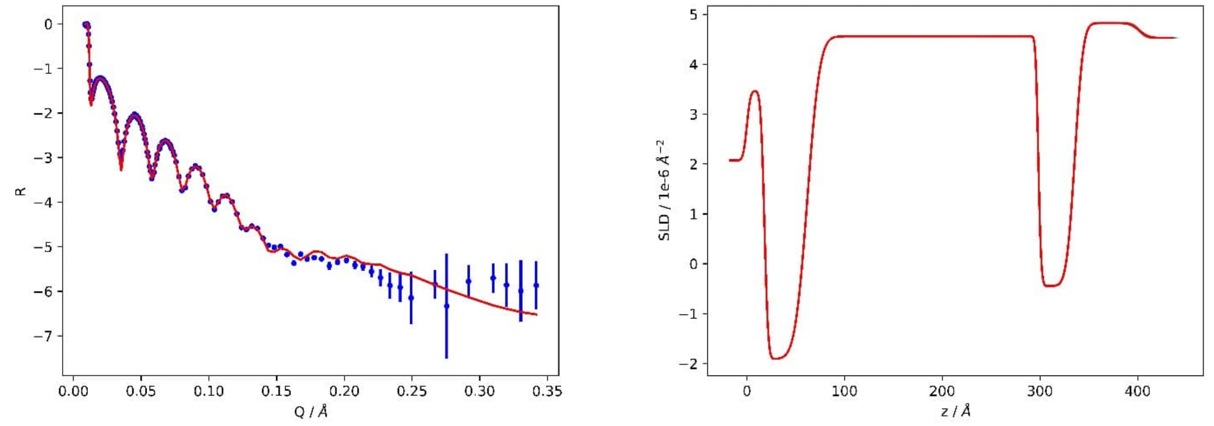

Fig. S53 EA thiol and 0.5 cmc d42-CTAC

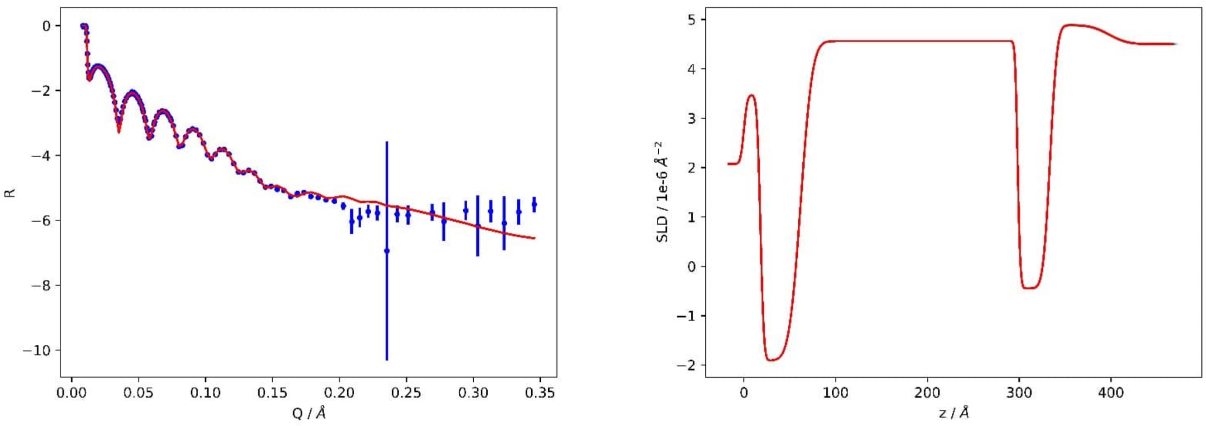

Fig. S54 EA thiol and 2 cmc d42-CTAC

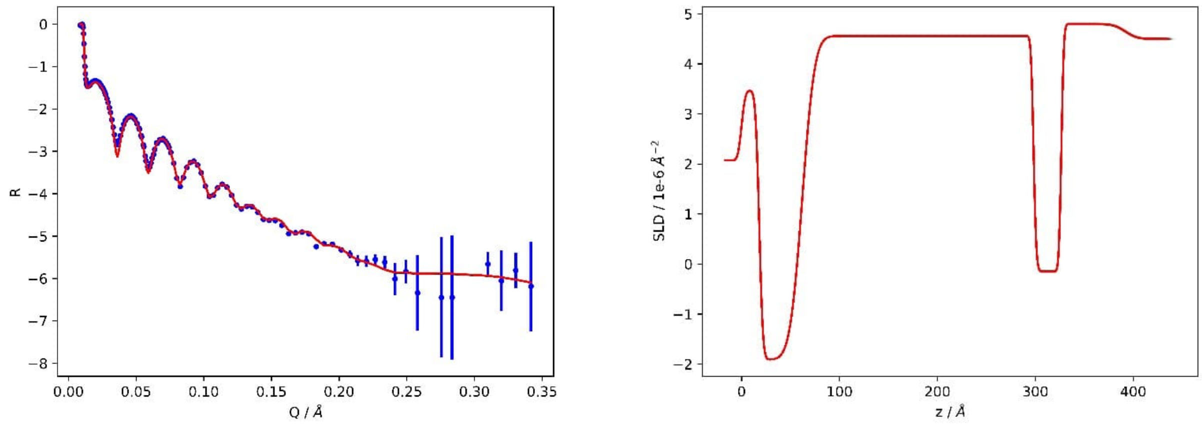

Fig. S55 EA thiol and 20 cmc d42-CTAC

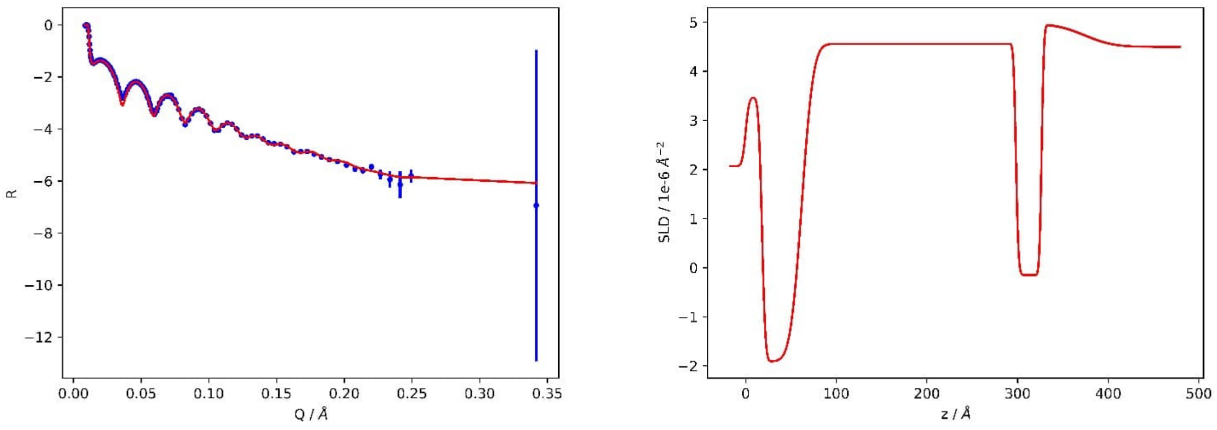

Fig. S56 EA thiol and 20 cmc d42-CTAC + 100 ppm chitosan oligomer

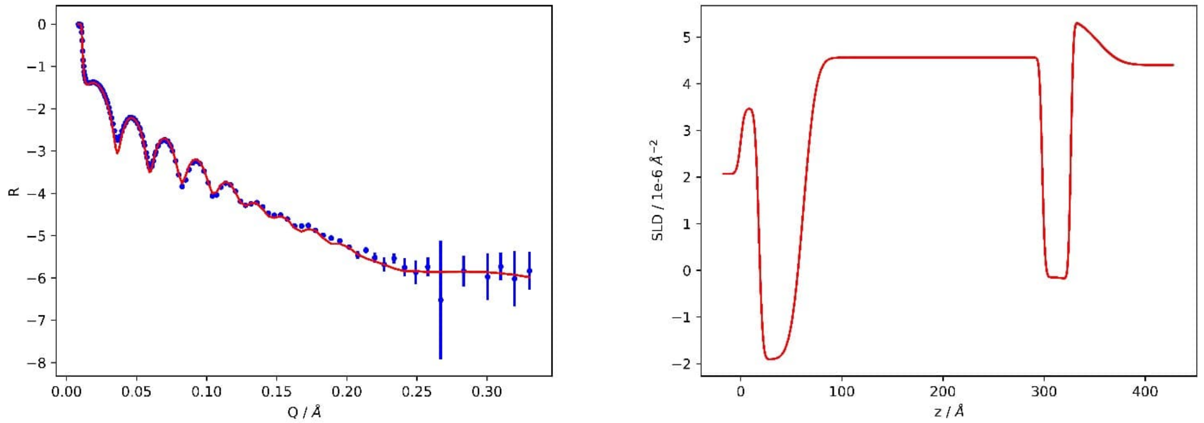

Fig. S57 EA thiol and 62.5 cmc d42-CTAC + 12.5 ppm chitosan oligomer

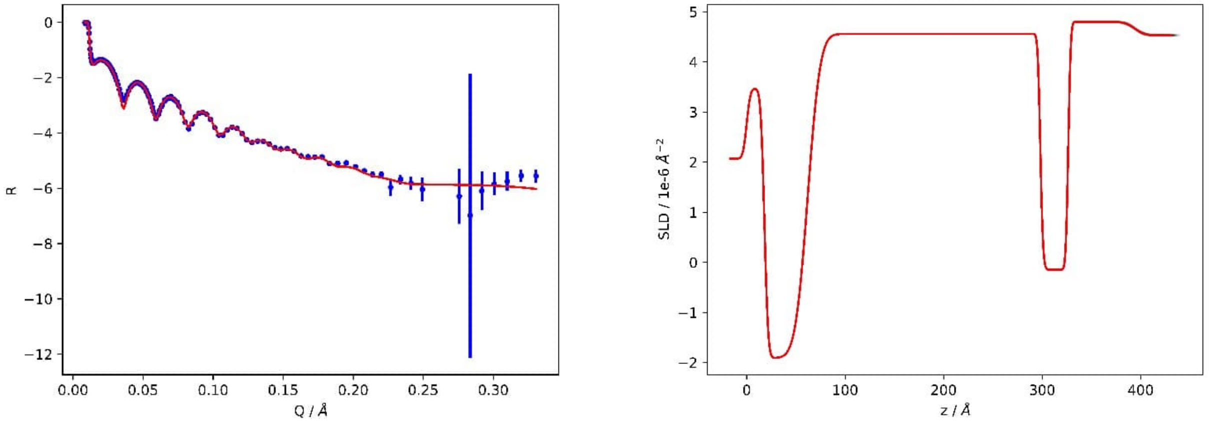

Fig. S58 EA thiol after rinse of d42-CTAC + chitosan oligomer

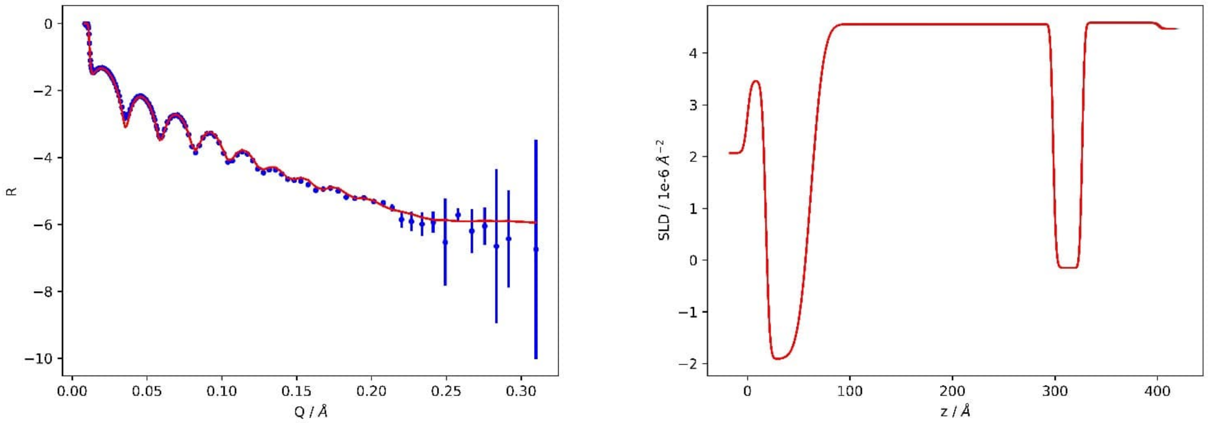

Fig. S59 EA thiol and 100 ppm chitosan oligomer

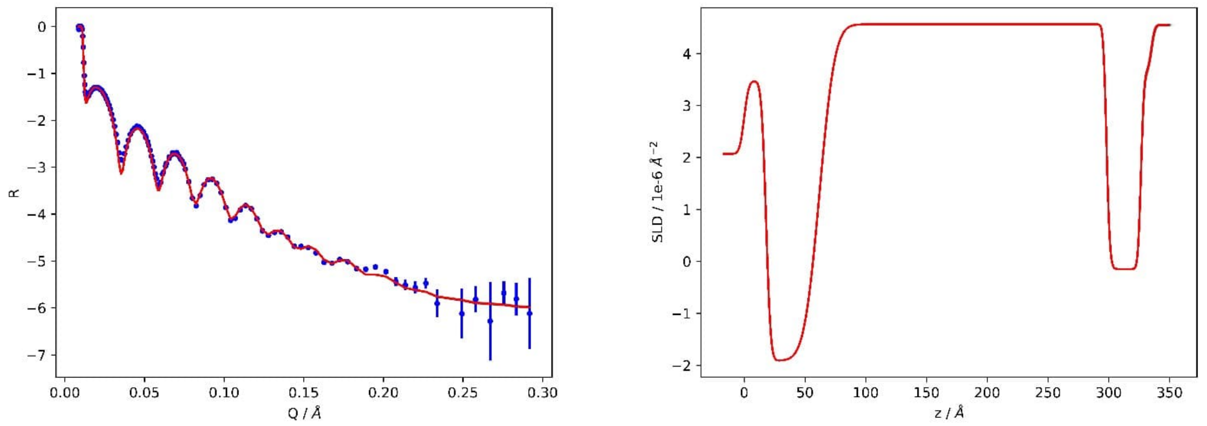

Fig. S60 EA thiol after rinse of chitosan oligomer

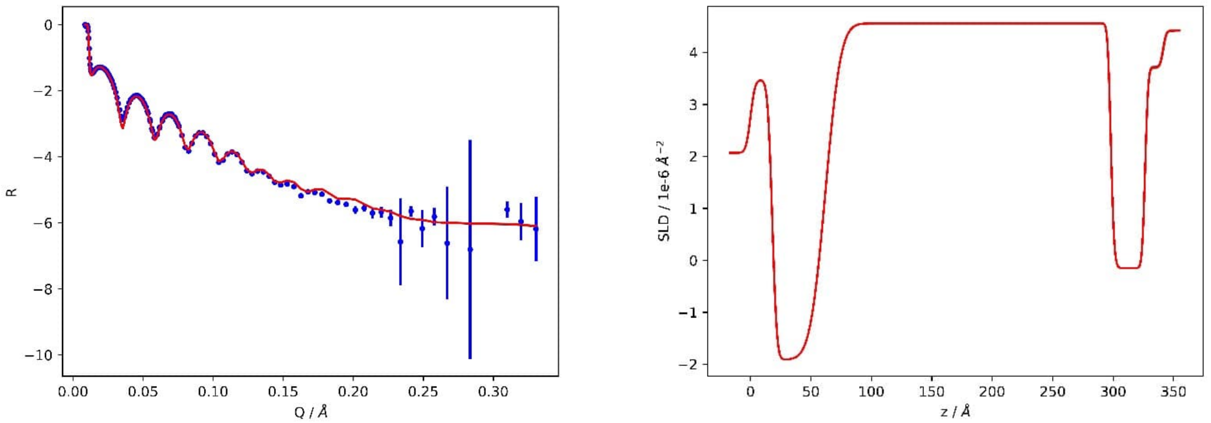

Fig. S61 EA thiol and 100 ppm chitosan polymer

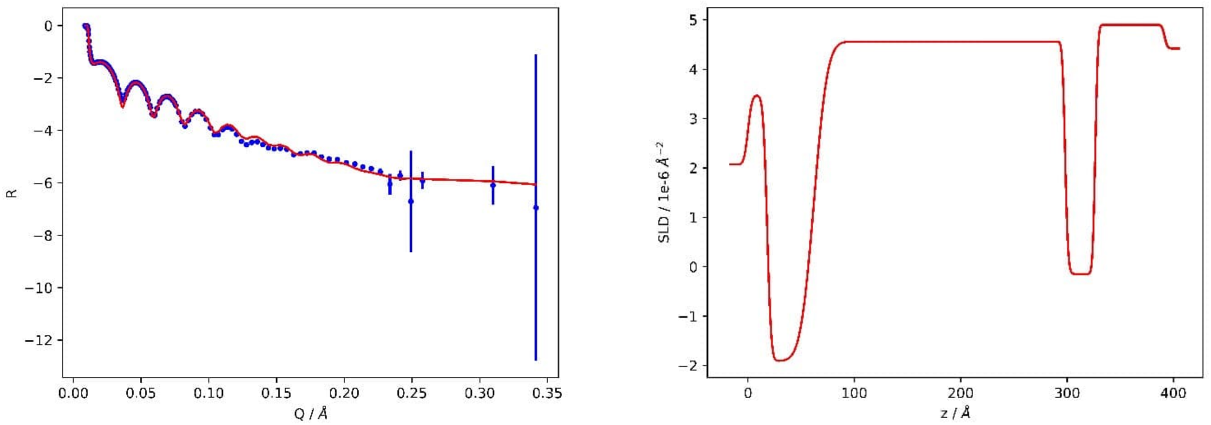

Fig. S62 EA thiol and 20 cmc d25-SDS (after chitosan polymer)

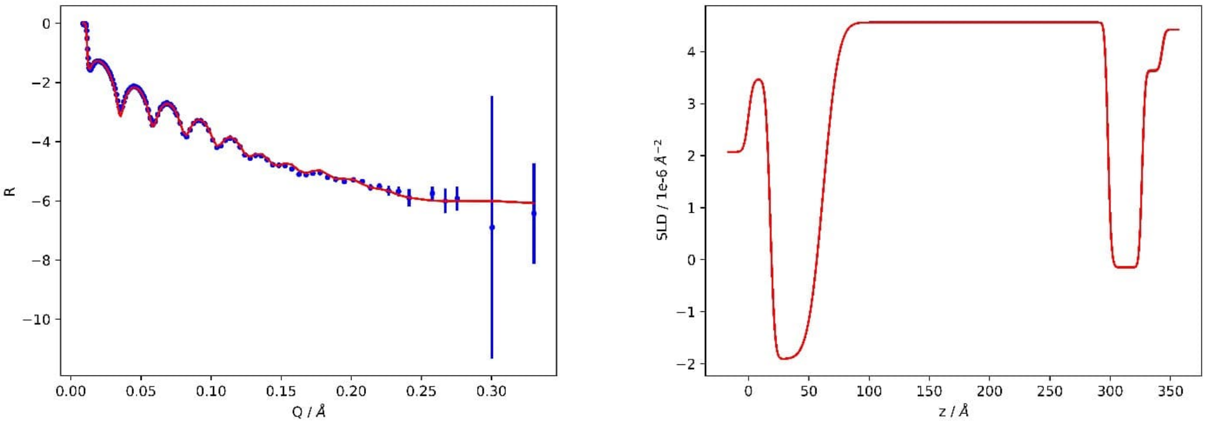

Fig. S63 EA thiol after rinse of d25-SDS

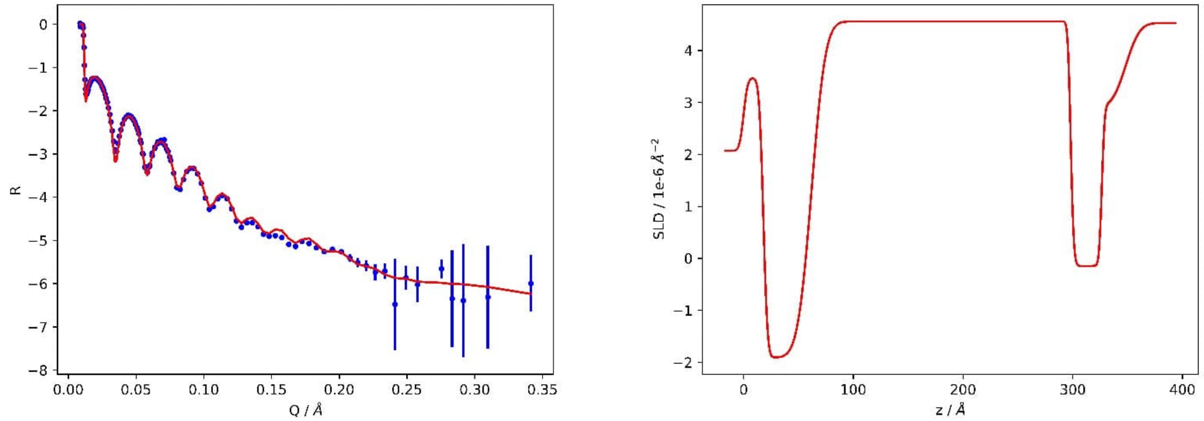

Fig. S64 EA thiol and 100 ppm pDADMAC

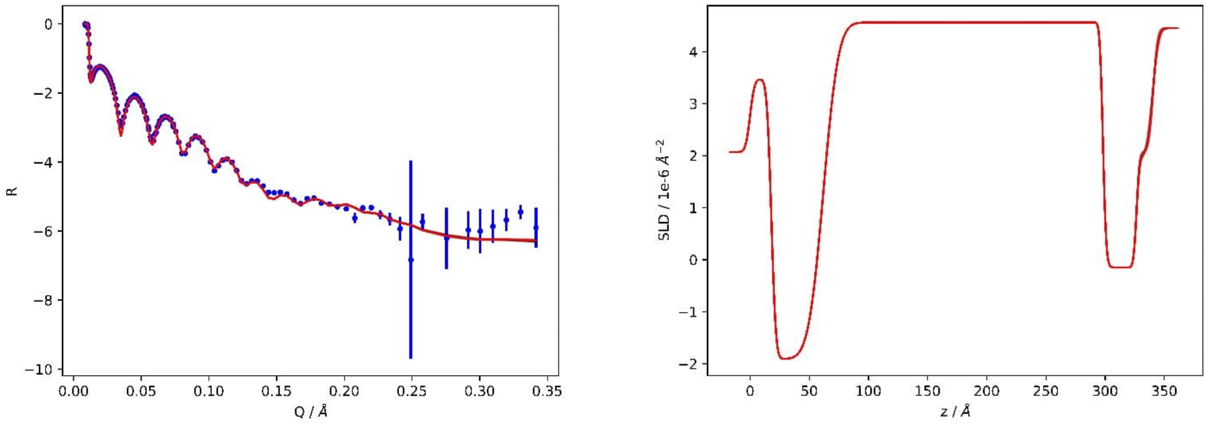

Fig. S65 EA thiol after rinse of pDADMAC

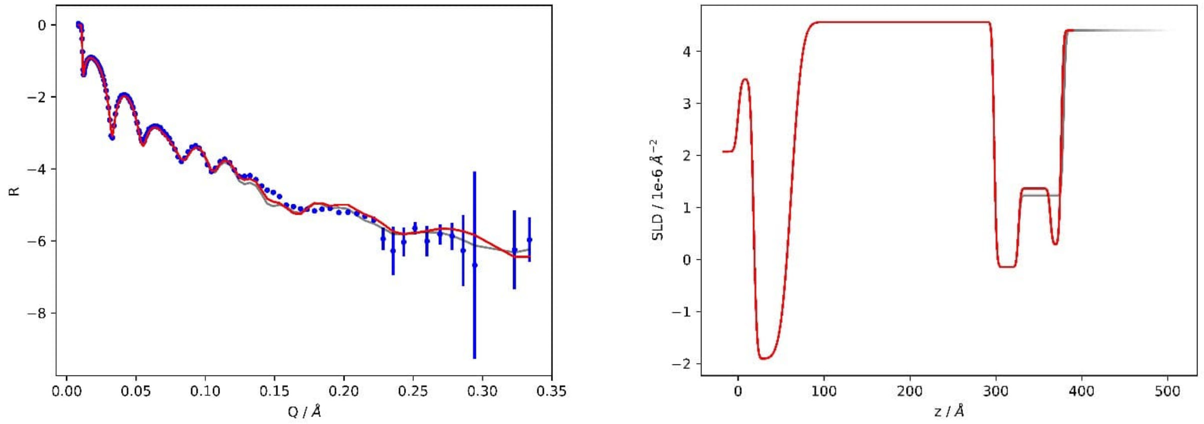

Fig. S66 EA thiol and 0.5 cmc h-SDS

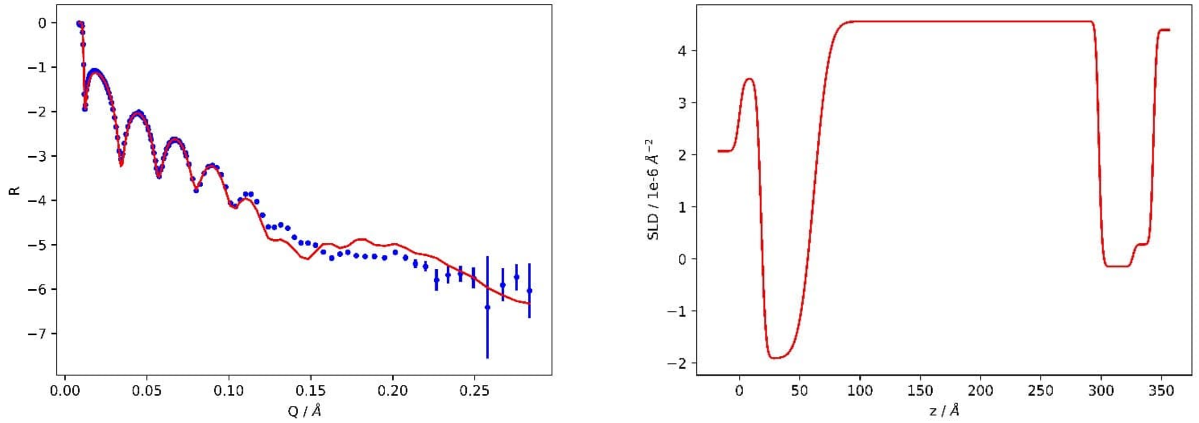

Fig. S67 EA thiol and 20 cmc h-SDS

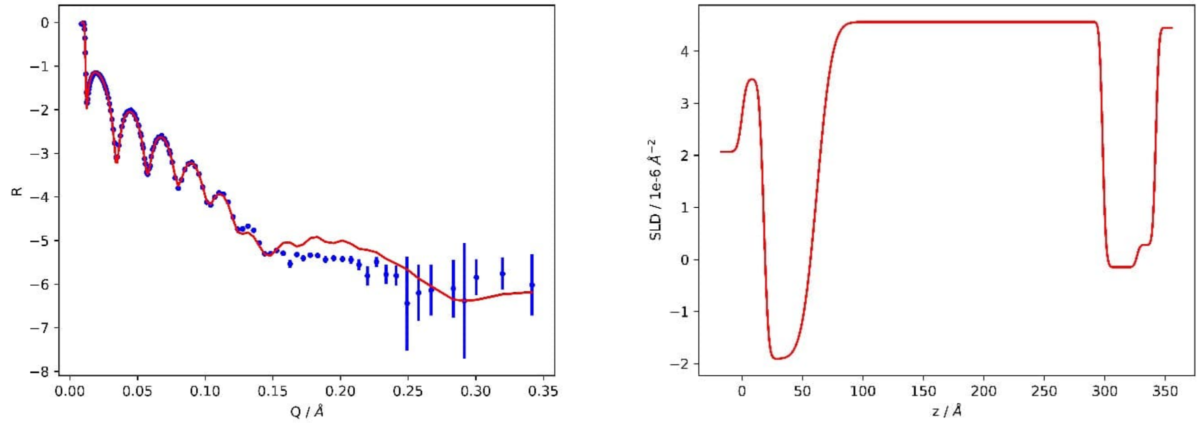

Fig. S68 EA thiol after final rinse

Table S7 Bulk SLD values and fitted layer thickness, roughness and SLD values for adsorbed layers on EA thiol surface (sequence NR1). Numbers in parenthesis indicate the error on the parameter value, equal to 2.5σ. The values in italics were manually adjusted

	bulk SLD ($*10^{-6}$Å^{-2})	SLD ($*10^{-6}$Å^{-2})	Thickness (Å)	Roughness (Å)
Thiol layer	4.540 (0.004)	*-0.004*	33.4 (0.4)	9.6 (0.6)
0.1 cmc d25-SDS	*4.48*	*6.2*	2 (1)	11 (3)
0.5 cmc d25-SDS	*4.51*	*6.2*	8 (1)	9 (3)
2 cmc d25-SDS	*4.51*	*6.2*	15.0 (0.7)	5.1 (0.2)
20 cmc d25-SDS	*4.52*	*6.2*	17.3 (0.8)	5.1 (0.2)
d25-SDS/oligomer	*4.52*	6.2 (0.3)	17 (2)	5 (4)
Rinse	*4.52*	6.19 (0.03)	13.4 (0.8)	5.2 (0.4)
20 cmc d25-SDS	*4.52*	6.2 (0.3)	18 (2)	5 (4)
Rinse	*4.52*	6 (2)	12 (17)	*5*

Table S8 Bulk SLD values and fitted layer thickness, roughness and SLD values for adsorbed layers on EA thiol surface (sequence NR2). Numbers in parenthesis indicate the error on the parameter value, equal to 2.5σ. The values in italics were manually adjusted. Up to 20 cmc d42-CTAC, the second slab of the thiol layer (EA chains) was fitted, plus an additional layer, after that the fit refers only to an additional slab

	bulk SLD ($*10^{-6}$Å^{-2})	SLD ($*10^{-6}$Å^{-2})	Thickness (Å)	Roughness (Å)
Thiol layer	*4.52*	*0.3*	*3*	*2*
		-0.450 (0.001)	36 (2)	2.0 (0.1)
		4 (3)	1 (3)	2 (2)
0.1 cmc d42-CTAC	*4.52*	-0.450 (0.001)	37.64 (0.07)	5.4 (0.1)
		4.75 (0.01)	67 (1)	2.2 (0.5)
0.5 cmc d42-CTAC	*4.53*	-0.448 (0.008)	34.80 (0.07)	6.8 (0.1)
		4.827 (0.009)	66 (1)	7 (3)
2 cmc d42-CTAC	*4.5*	-0.449 (0.003)	32.79 (0.07)	6.0 (0.1)
		4.89 (0.01)	64 (1)	16 (2)
20 cmc d42-CTAC	*4.5*	-0.15 (0.02)	25.54 (0.09)	*2*
		4.80 (0.01)	65 (1)	10 (2)
d42-CTAC/oligomer 1	*4.5*	4.96 (0.02)	47 (2)	24.9 (0.4)
d42-CTAC/oligomer 2	*4.4*	5.42 (0.03)	26.7 (0.8)	17.0 (0.6)
Rinse	*4.53*	4.800 (0.008)	68 (1)	8 (2)
Chitosan oligomer	*4.47*	4.582 (0.006)	75 (2)	2 (1)
Rinse	*4.55*	3.6 (0.1)	8.6 (0.8)	2.2 (0.6)
Chitosan polymer	*4.42*	3.71 (0.03)	14.9 (0.5)	2.0 (0.1)
20 cmc d25-SDS	*4.42*	4.899 (0.005)	65.2 (0.5)	2.0 (0.1)
Rinse	*4.42*	3.63 (0.02)	16.6 (0.4)	2.02 (0.06)
pDADMAC	*4.53*	2.90 (0.03)	21.8 (0.3)	*10*
Rinse	*4.45*	2.0 (0.1)	13.8 (0.6)	4 (1)
0.5 cmc h-SDS	*4.4*	1.36 (0.01)	37.0 (0.3)	2.0 (0.1)
		0.28	12.7 (0.2)	2.03 (0.09)
20 cmc h-SDS	*4.4*	0.280 (0.001)	16.97 (0.07)	2.01 (0.02)
Rinse	*4.45*	0.280 (0.001)	15.61 (0.07)	2.01 (0.03)

S5.3 Mixed PS:18-MEA thiol surface

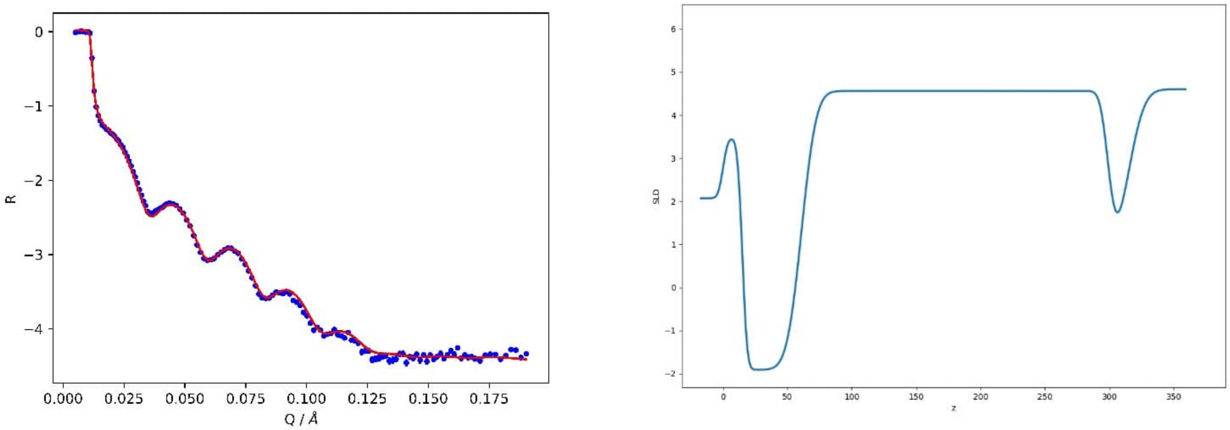

Fig. S69 80:20 PS:18-MEA thiol

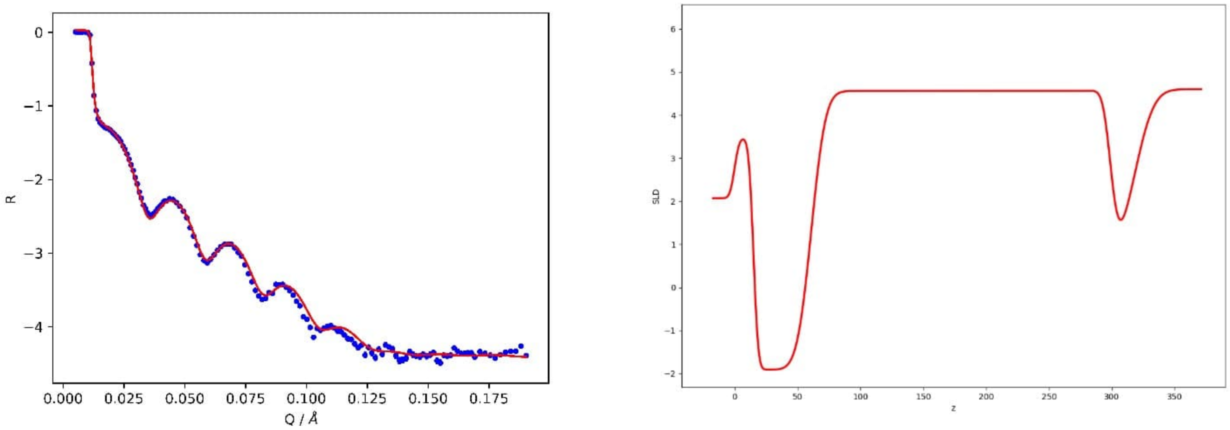

Fig. S70 80:20 PS:18-MEA thiol and 2 cmc d25-SDS

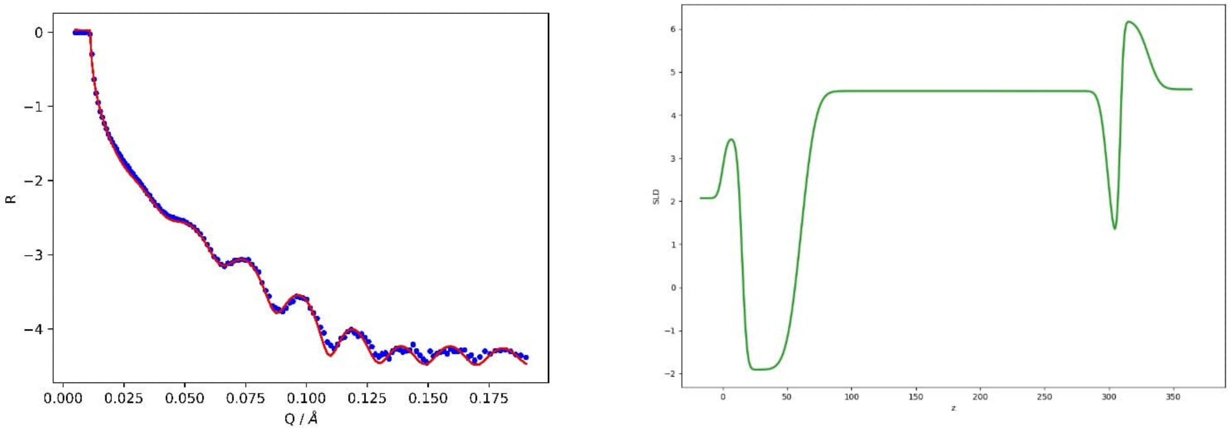

Fig. S71 80:20 PS:18-MEA thiol and 20 cmc d25-SDS

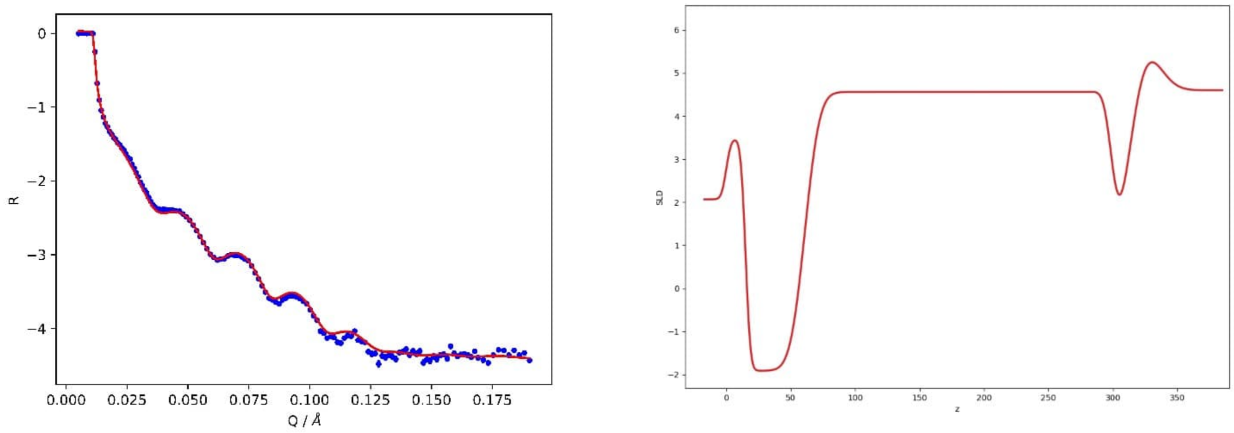

Fig. S72 80:20 PS:18 MEA thiol after rinse of d25-SDS

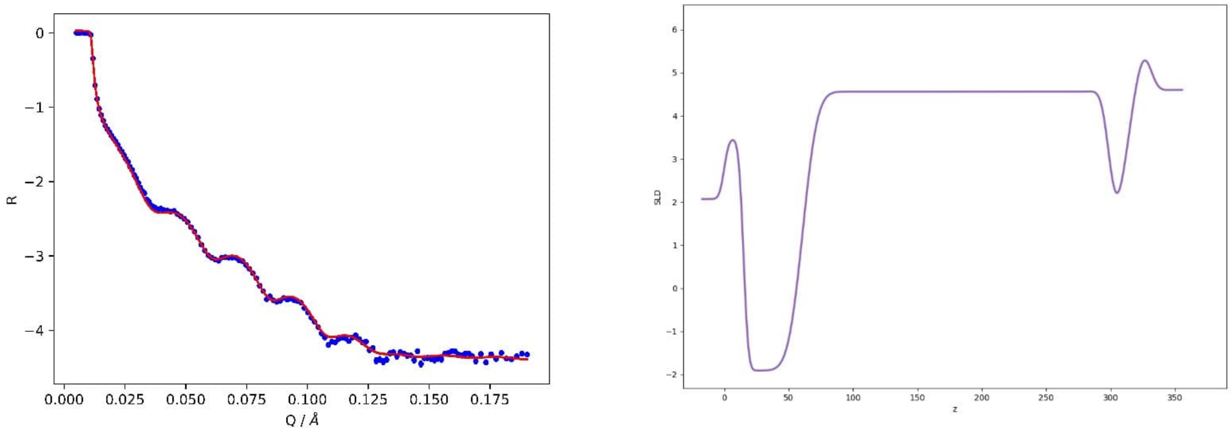

Fig. S73 80:20 PS:18-MEA thiol and 100 ppm chitosan oligomer

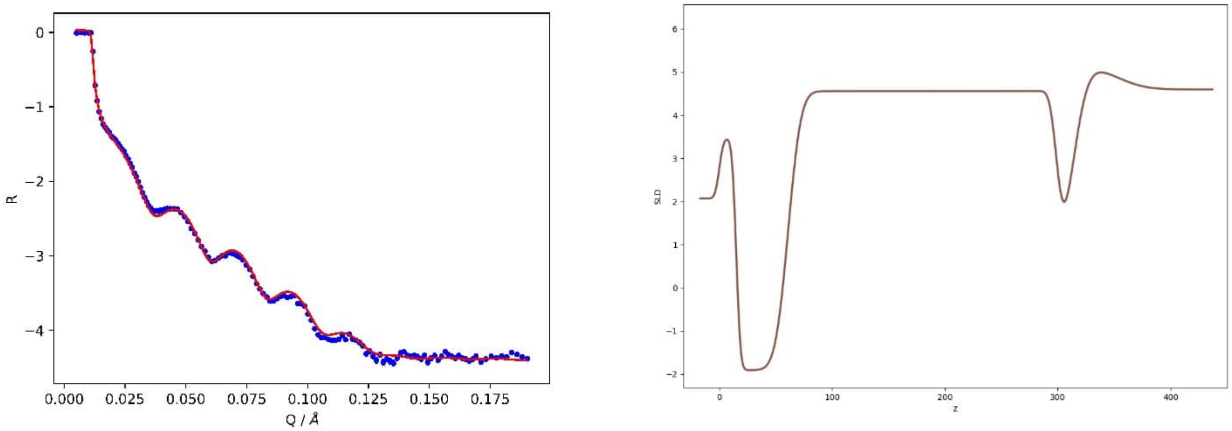

Fig. S74 80:20 PS:18-MEA thiol after rinse of chitosan oligomer

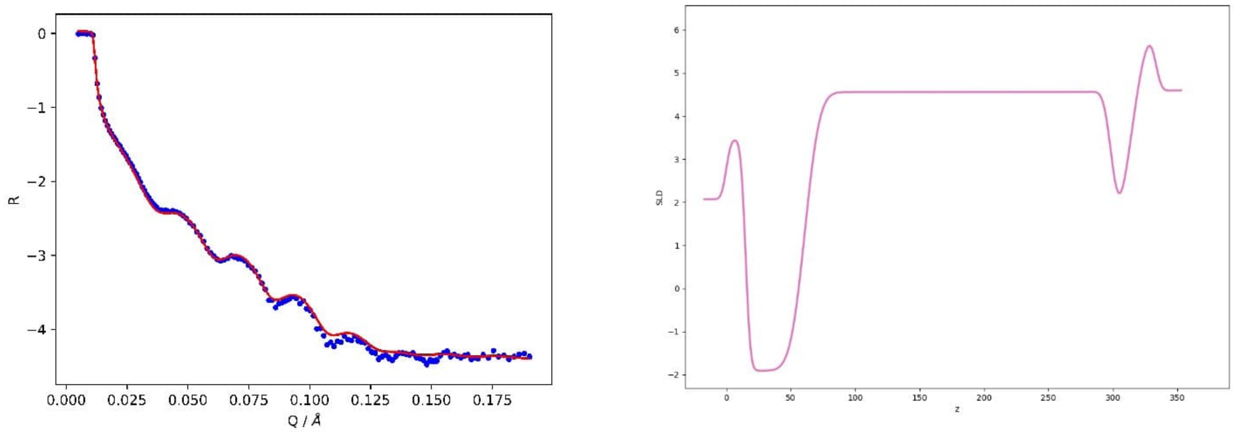

Fig. S75 80:20 PS:18 MEA thiol and 2 cmc d25-SDS (after rinse)

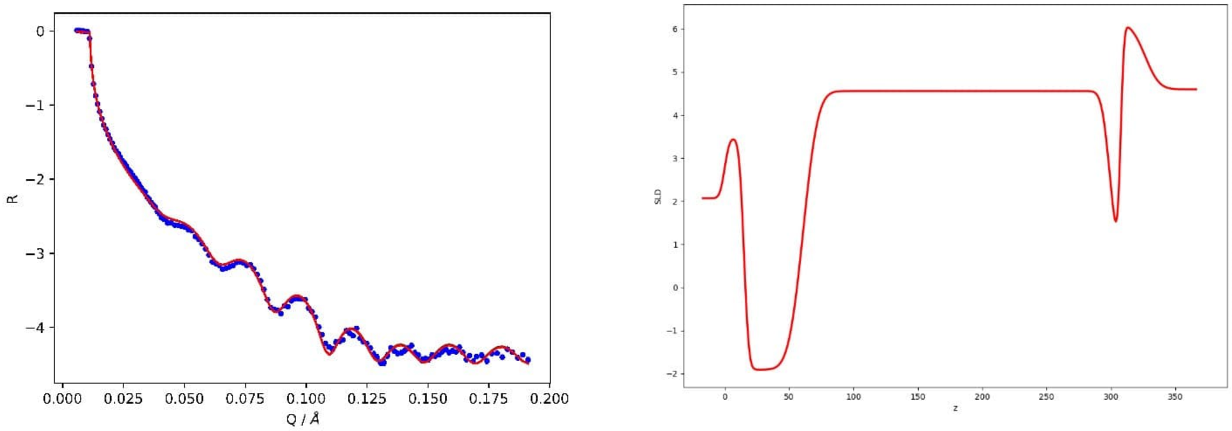

Fig. S76 80:20 PS:18-MEA thiol and 20 cmc d25-SDS + 100 ppm chitosan oligomer

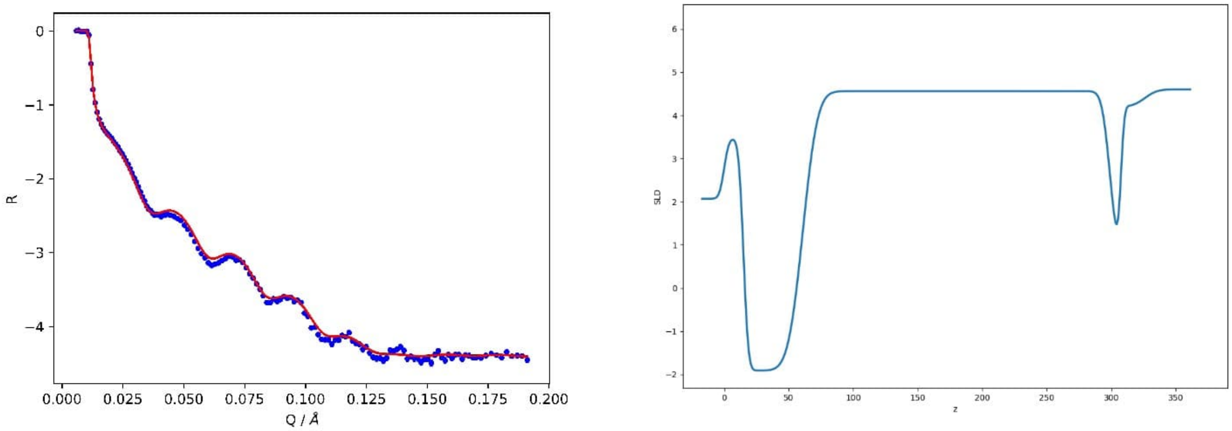

Fig. S77 80:20 PS:18-MEA thiol after rinse of d25-SDS + chitosan oligomer

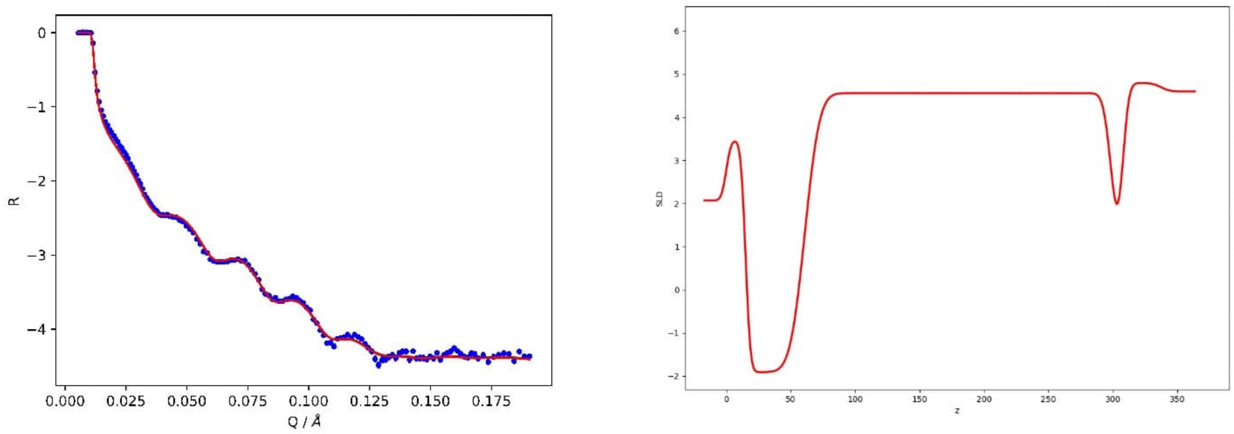

Fig. S78 80:20 PS:18 MEA thiol and 0.1 cmc d42-CTAC

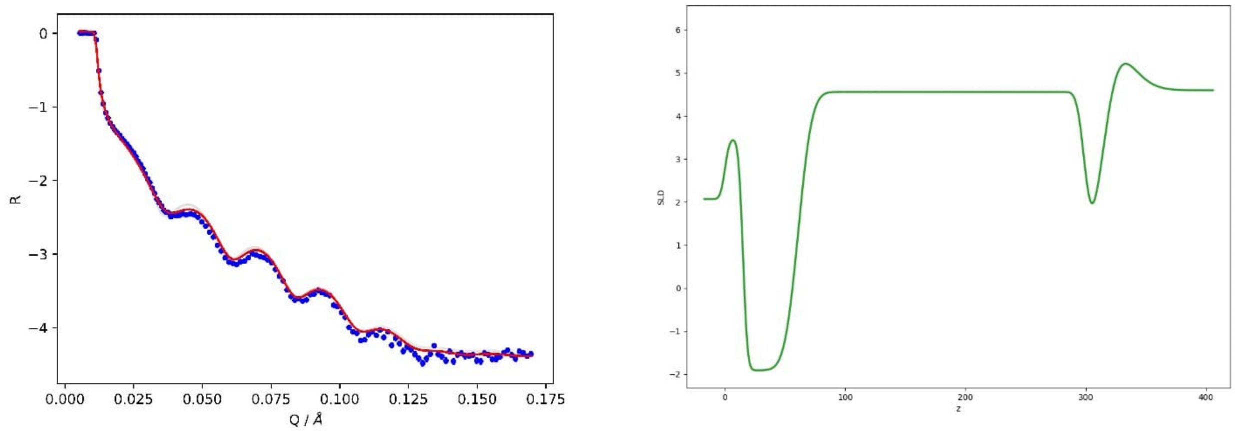

Fig. S79 80:20 PS:18-MEA thiol and 0.5 cmc d42-CTAC

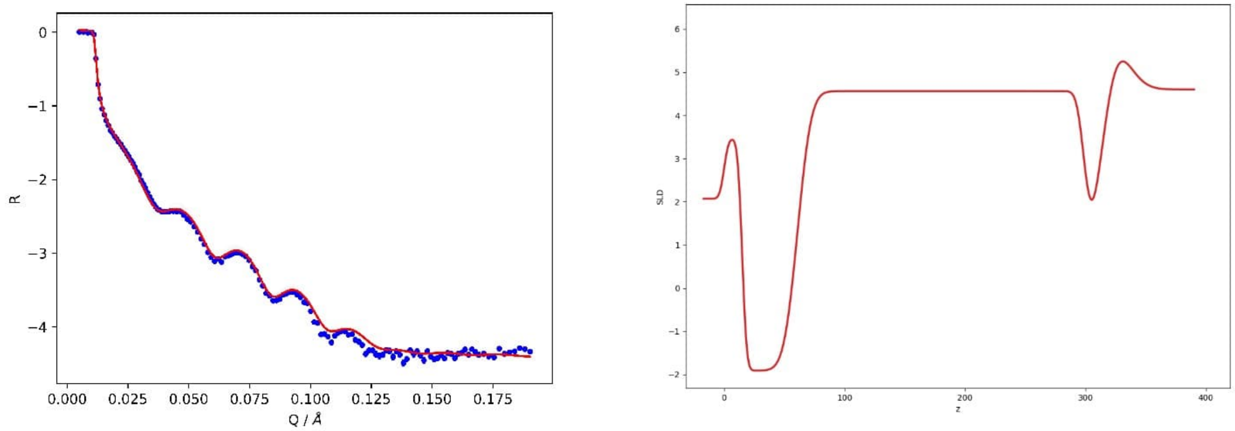

Fig. S80 80:20 PS:18-MEA thiol and 2 cmc d42-CTAC

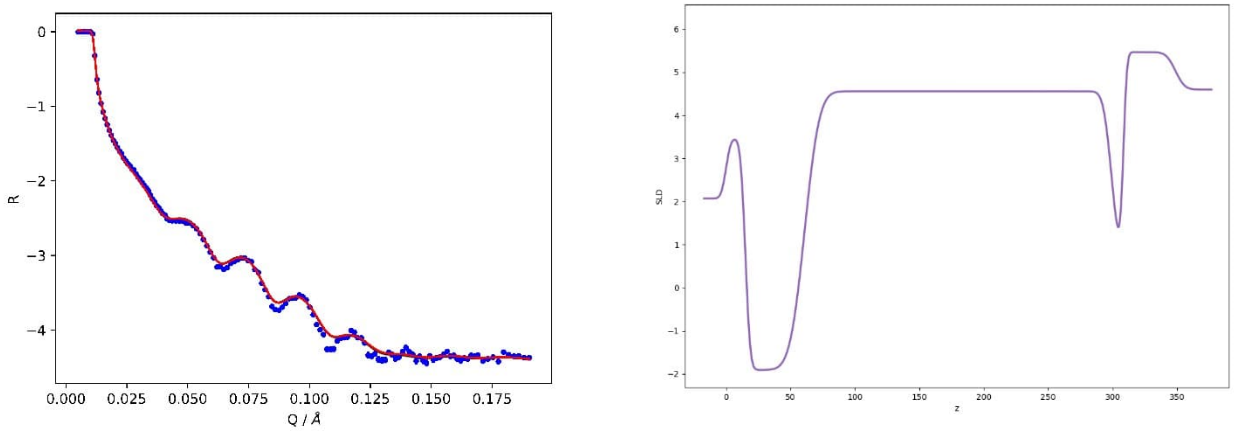

Fig. S81 80:20 PS:18 MEA thiol and 20 cmc d42-CTAC

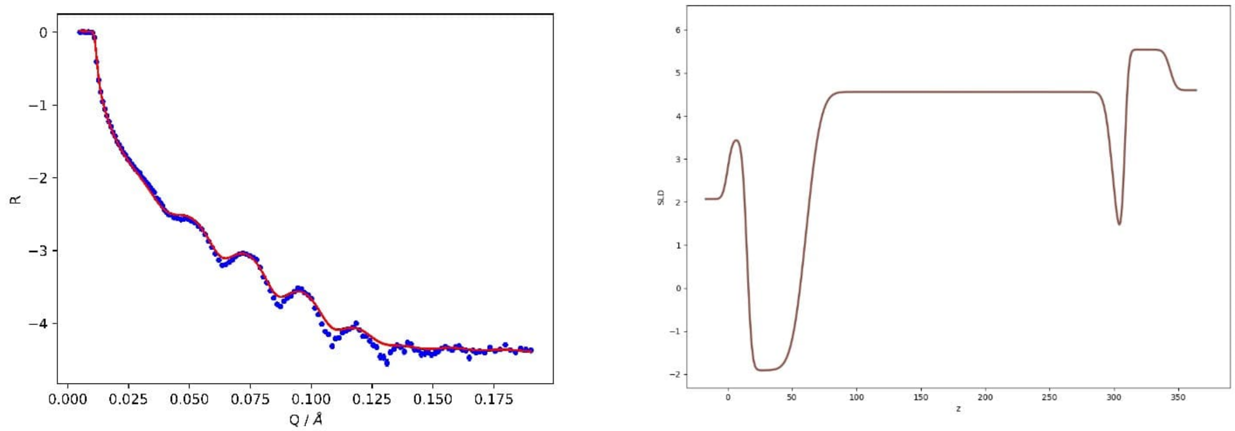

Fig. S82 80:20 PS:18-MEA thiol and 20 cmc d42-CTAC + 100 ppm chitosan oligomer

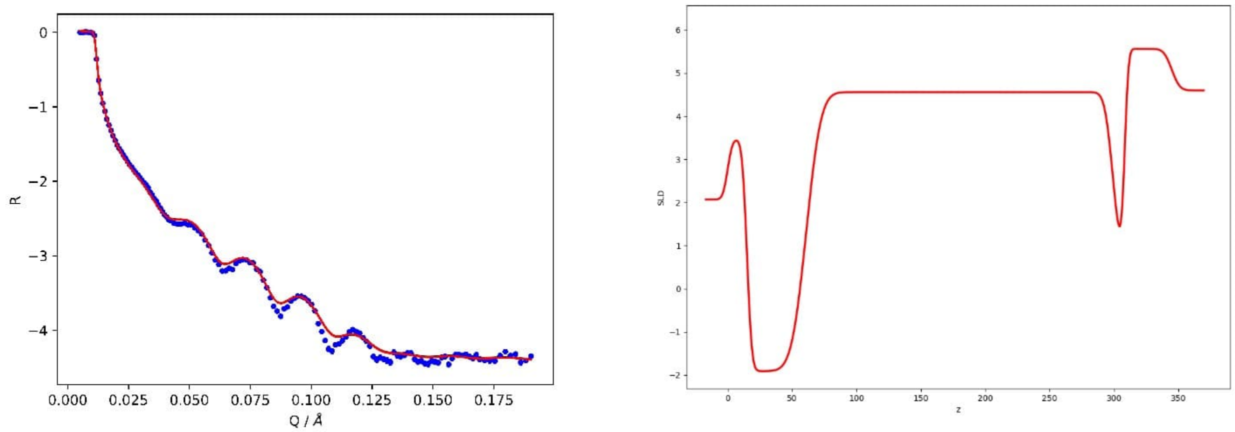

Fig. S83 80:20 PS:18-MEA thiol and 62.5 cmc d42-CTAC + 12.5 ppm chitosan oligomer

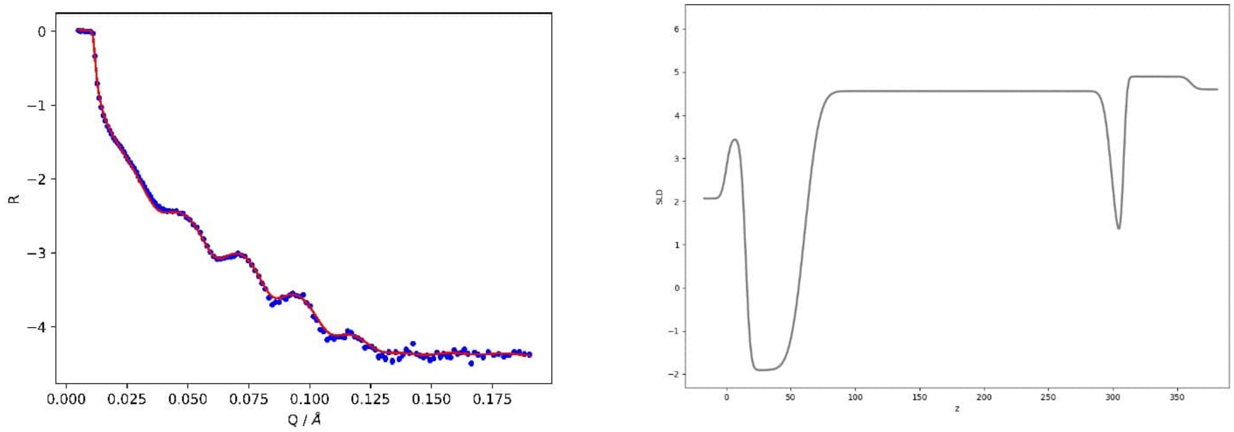

Fig. S84 80:20 PS:18 MEA thiol after rinse of d42-CTAC + chitosan oligomer

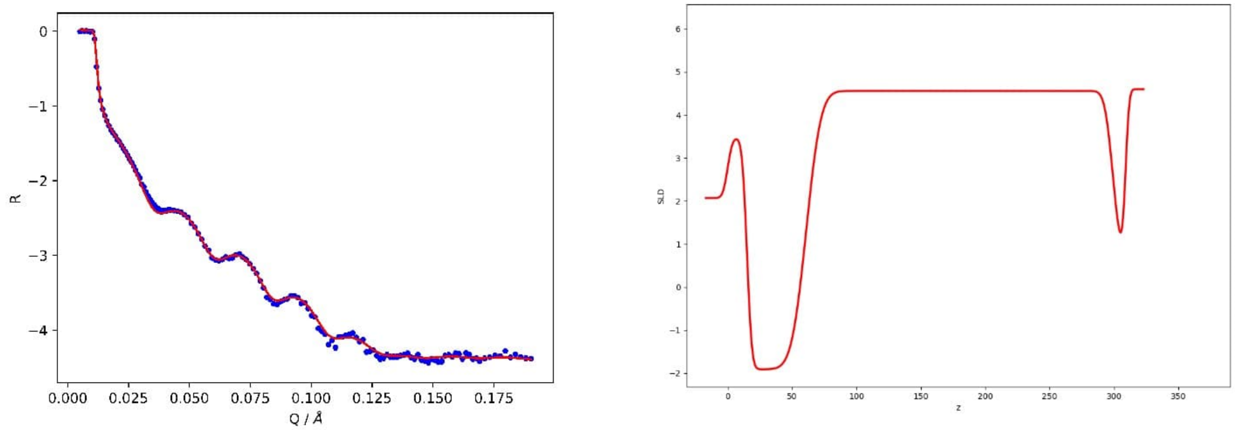

Fig. S85 80:20 PS:18-MEA thiol and 100 ppm chitosan polymer

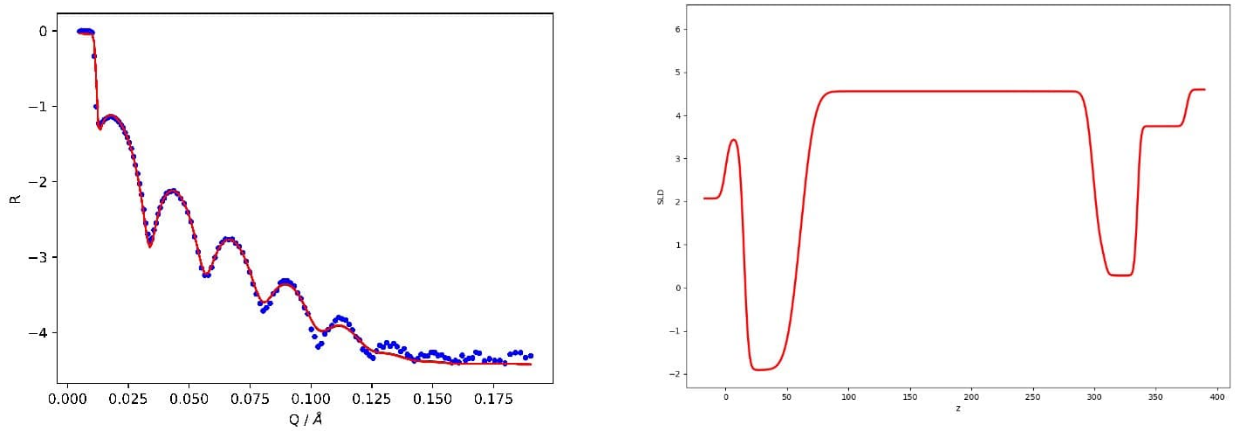

Fig. S86 80:20 PS:18-MEA thiol and 20 cmc h-SDS

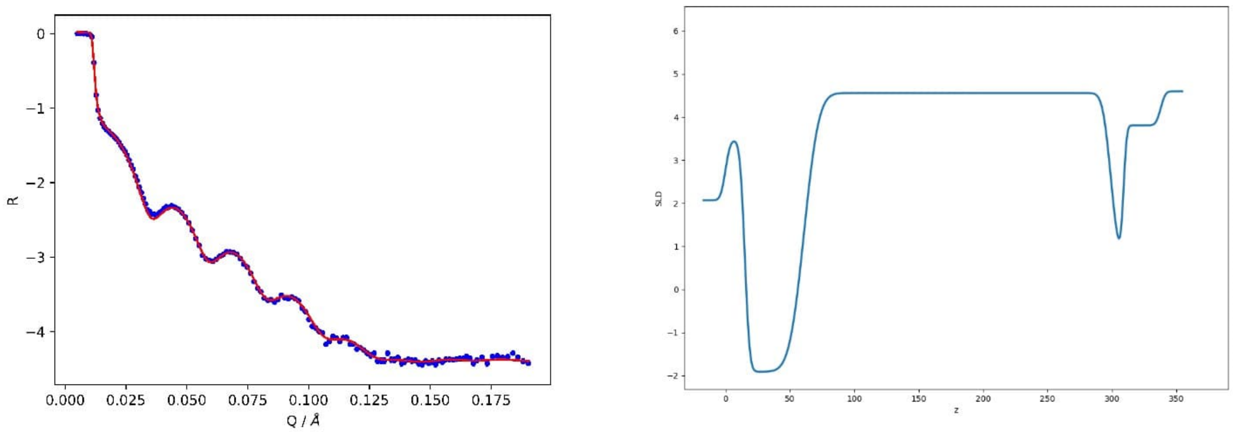

Fig. S87 80:20 PS:18 MEA thiol after rinse of h-SDS

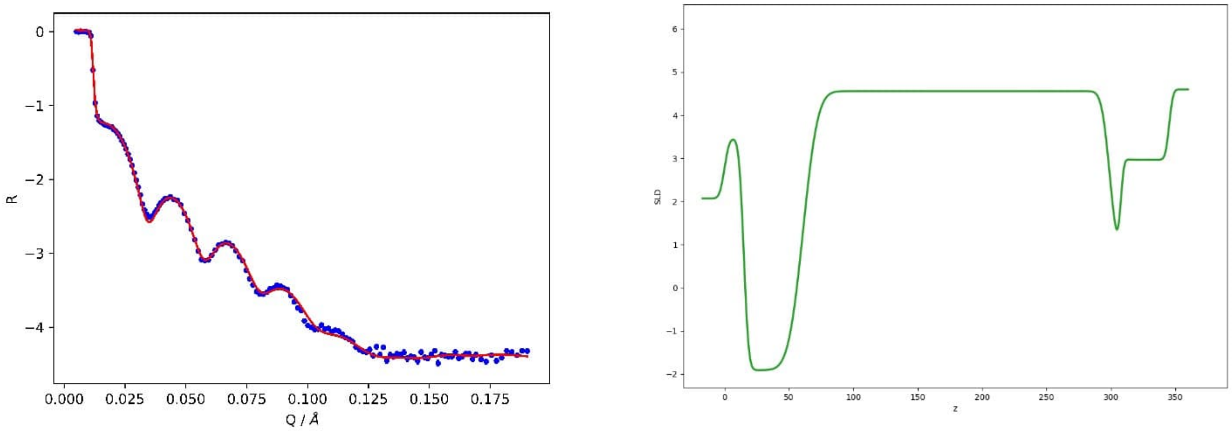

Fig. S88 80:20 PS:18-MEA thiol and 100 ppm pDADMAC

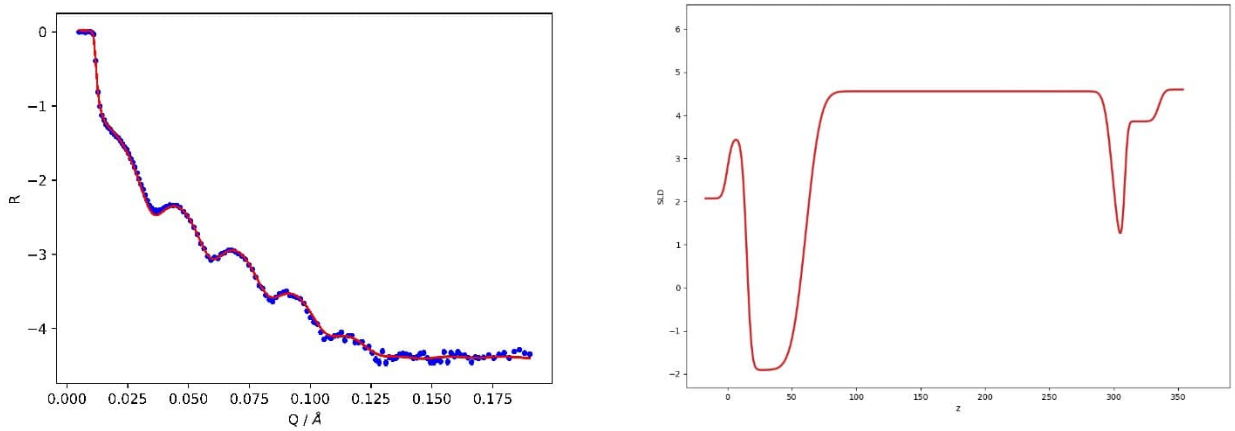

Fig. S89 80:20 PS:18-MEA thiol after final rinse

Table S9 Bulk SLD values and fitted layer thickness, roughness and SLD values for adsorbed layers on 80:20 PS:18-MEA thiol surface (sequence NR3). Numbers in parenthesis indicate the error on the parameter value, equal to 2.5σ. The values in italics were manually adjusted. When not indicated, the thiol layer (layer of SLD 0.7*10^{-6}Å^{-2}) is the same as in the previous step

	bulk SLD ($*10^{-6}$Å^{-2})	SLD ($*10^{-6}$Å^{-2})	Thickness (Å)	Roughness (Å)
Thiol layer	*4.6*	0.7 (0.2)	15 (1)	10 (1)
2 cmc d25-SDS	*4.6*	*0.7*	19.2 (0.2)	11.98 (0.05)
20 cmc d25-SDS	*4.6*	*0.7*	9.6 (0.2)	2.2 (0.5)
		6.2	21.8 (0.7)	7 (2)
Rinse	*4.6*	*0.7*	14.6 (0.7)	10 (1)
		6.2	19 (2)	11.9 (0.4)
Chitosan oligomer	*4.6*	6.19 (0.03)	16.8 (0.7)	2 (1)
Rinse	*4.6*	5.1 (0.1)	39 (10)	19.8 (0.5)
2 cmc d25-SDS	*4.6*	6.19 (0.01)	19.4 (0.6)	4 (2)
d25-SDS/oligomer	*4.6*	*0.7*	8.4 (0.3)	2.1 (0.2)
		6.1 (0.1)	19 (2)	9 (2)
Rinse	*4.6* 4.2 (0.2)	18 (10)	8 (6)	
0.1 cmc d42-CTAC	*4.6*	4.79 (0.07)	30 (10)	5 (6)
0.5 cmc d42-CTAC	*4.6*	*0.7*	*15.5*	10.0 (0.1)
		6.2 (0.5)	18 (6)	17 (2)
2 cmc d42-CTAC	*4.6*	6.9 (0.5)	14 (3)	14 (1)
20 cmc d42-CTAC	*4.6*	*0.7*	9.3 (0.4)	2.2 (0.5)
		5.5 (0.1)	40 (3)	6 (6)
d42-CTAC/oligomer 1	*4.6*	5.54 (0.06)	35 (2)	4 (3)
d42-CTAC/oligomer 2	*4.6*	5.56 (0.06)	36 (2)	5 (5)
Rinse	*4.6*	4.90 (0.03)	52 (4)	4 (4)
Chitosan polymer	*4.6*	*0.7*	9.9 (0.2)	2.2 (0.6)
20 cmc h-SDS	*4.6*	0.282 (0.007)	26.1 (0.7)	2.1 (0.2)
		3.75 (0.08)	40 (2)	2 (1)
Rinse	*4.6*	3.81 (0.07)	29 (2)	3 (2)
pDADMAC	*4.6*	*0.7*	8.5 (0.2)	*2*
		2.97 (0.05)	37.6 (0.9)	2 (1)
Rinse	*4.6*	*0.7*	9.4 (0.9)	*2*
		3.9 (0.2)	27 (4)	3 (4)

S5.4 PS surface

Unfortunately, the NR curves relative to the lower concentrations of d42-CTAC are very close to the curve in pure solvent (see Figure S85 below) so that it is not possible to reliably define an additional slab to see how adsorption builds up with concentration. Similarly, adsorption could not be defined in some other case indicated in Figure S90. Fits for the other steps can be found from Figure S91 on.

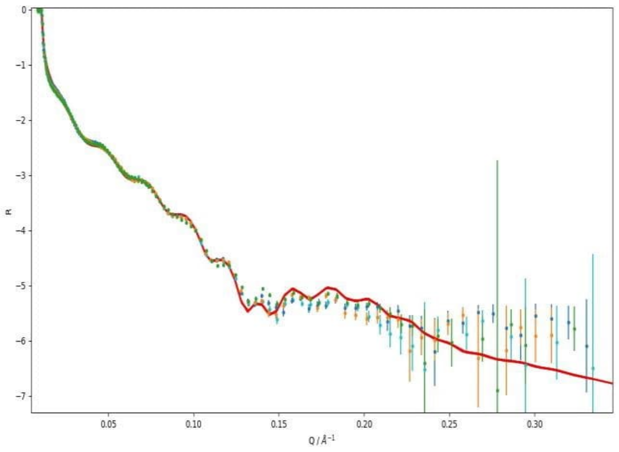

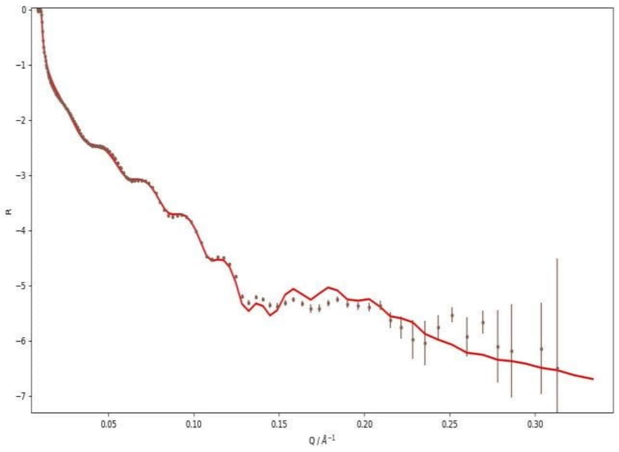

(a)

(b)

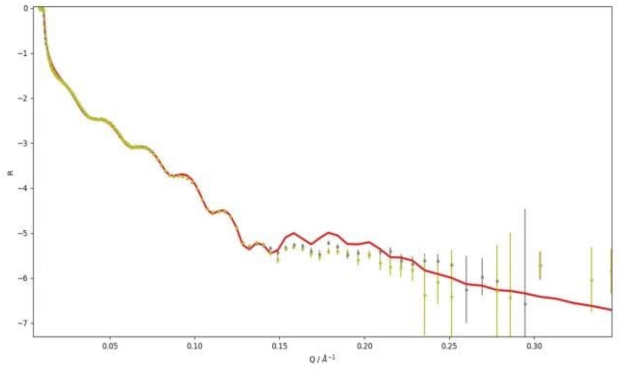

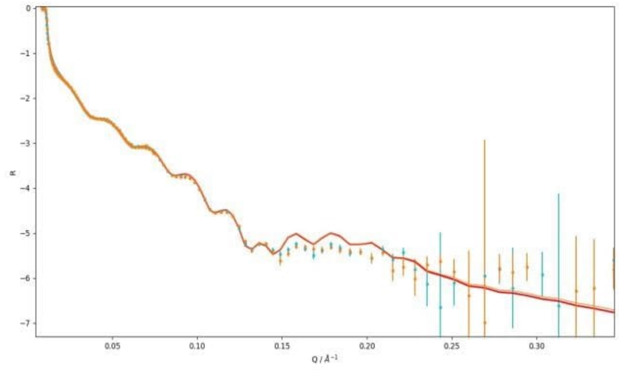

(c)

(d)

Fig. S90 Fitting of NR data of PS in GCMW and 100 mM NaCl in the presence of a) 0.1 (orange), 0.5 (blue) and 2 (green) cmc d42CTAC, b) 62.5 d42-CTAC and 12.5 chitosan oligomer, c) 100 ppm chitosan oligomer (grey) and corresponding rinse (yellow), d) 100 ppm chitosan polymer (blue) and rinse (orange - adsorption of d25-SDS in between). The fit is the same as in pure solvent.

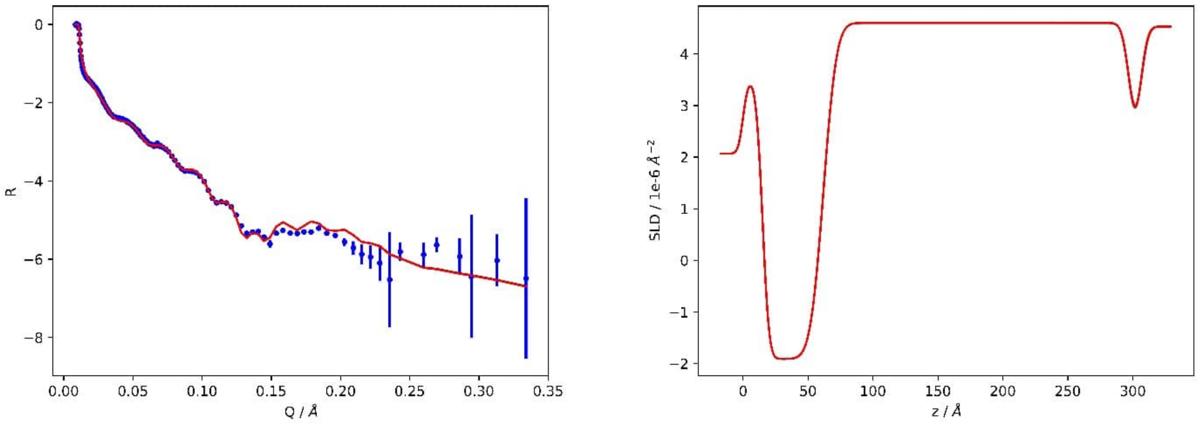

Fig. S91 PS

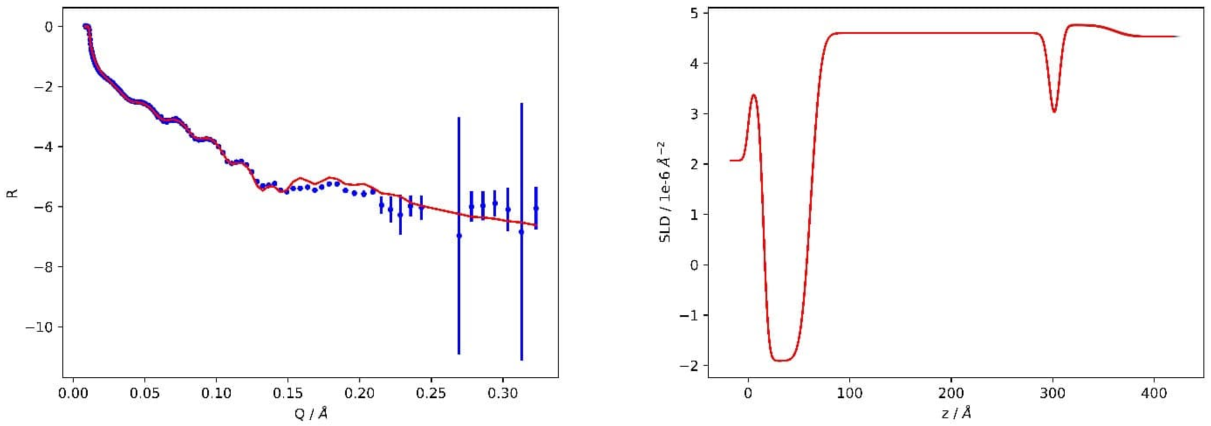

Fig. S92 PS and 20 cmc d42-CTAC

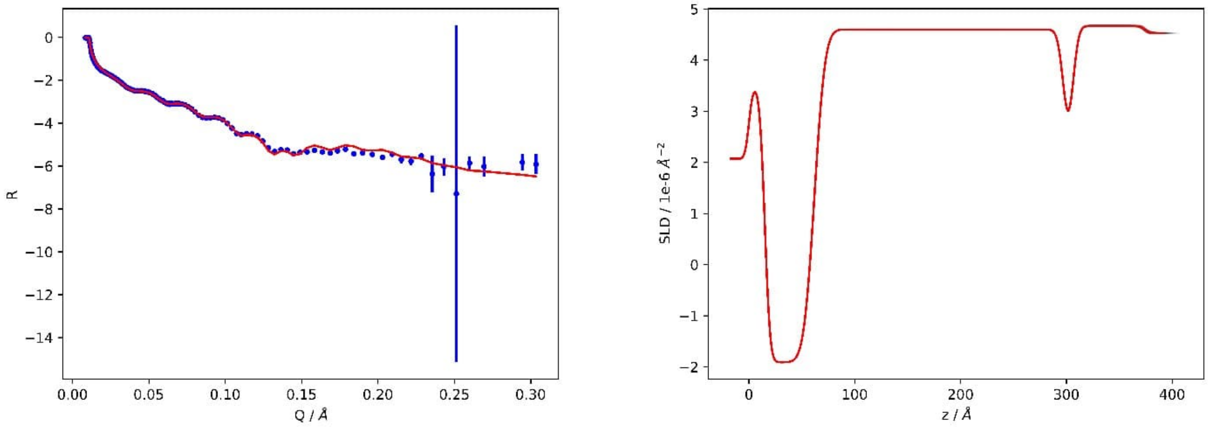

Fig. S93 PS and 20 cmc d42-CTAC + 100 ppm chitosan oligomer

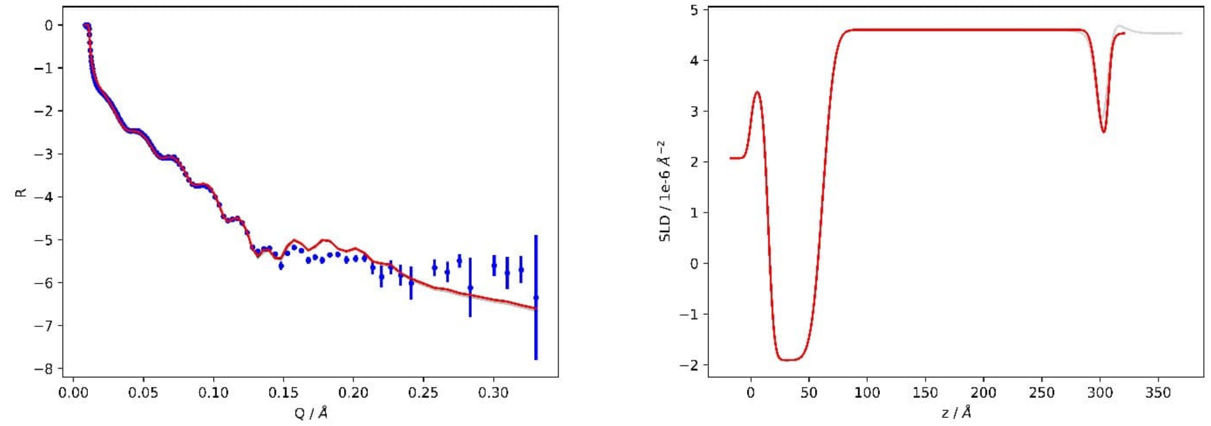

Fig. S94 PS after rinse of d42-CTAC + chitosan oligomer

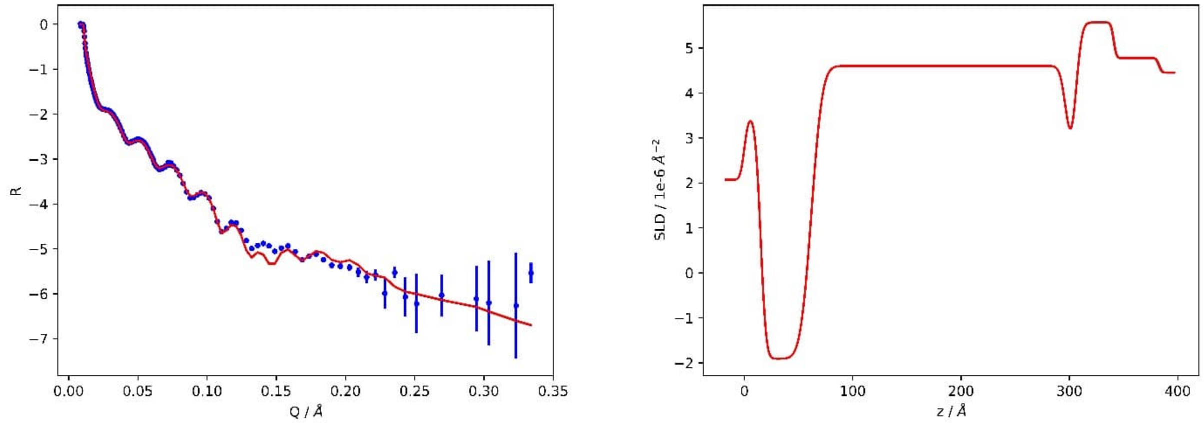

Fig. S95 PS and 20 cmc d25-SDS (after chitosan polymer)

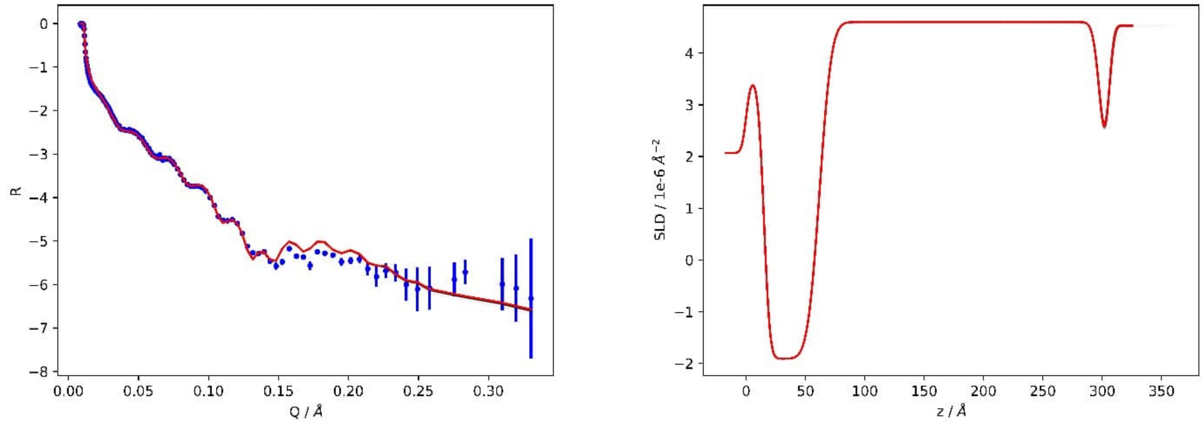

Fig. S96 PS and 100 ppm pDADMAC

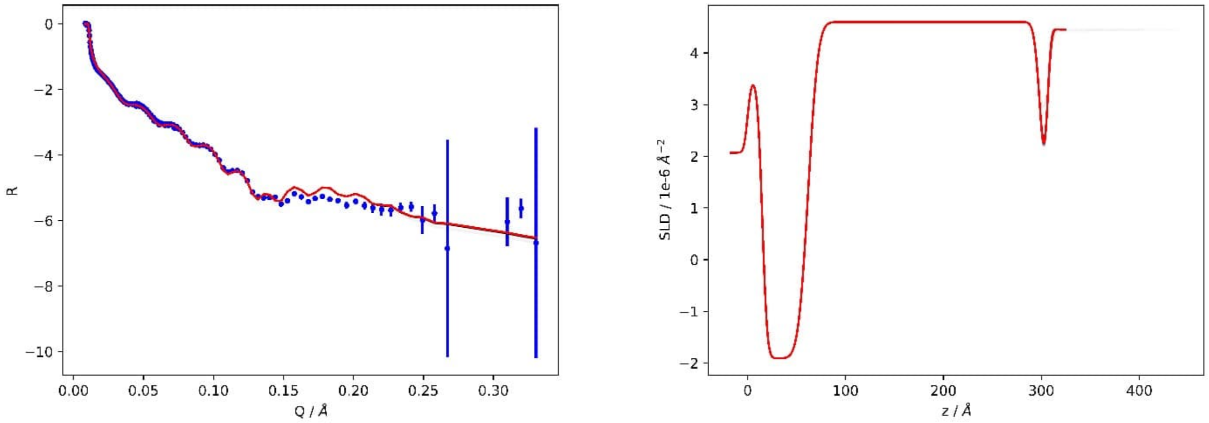

Fig. S97 PS after rinse of pDADMAC

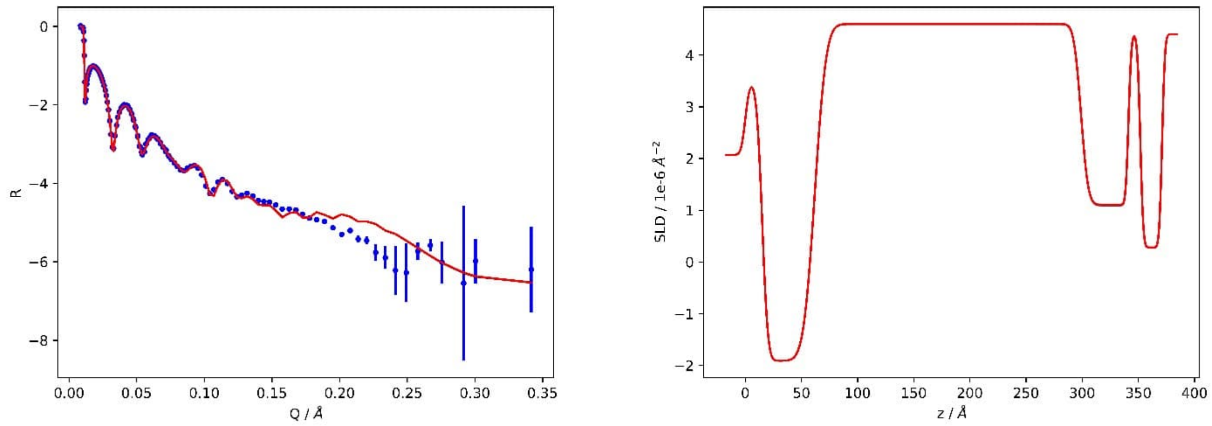

Fig. S98 PS and 0.5 cmc h-SDS

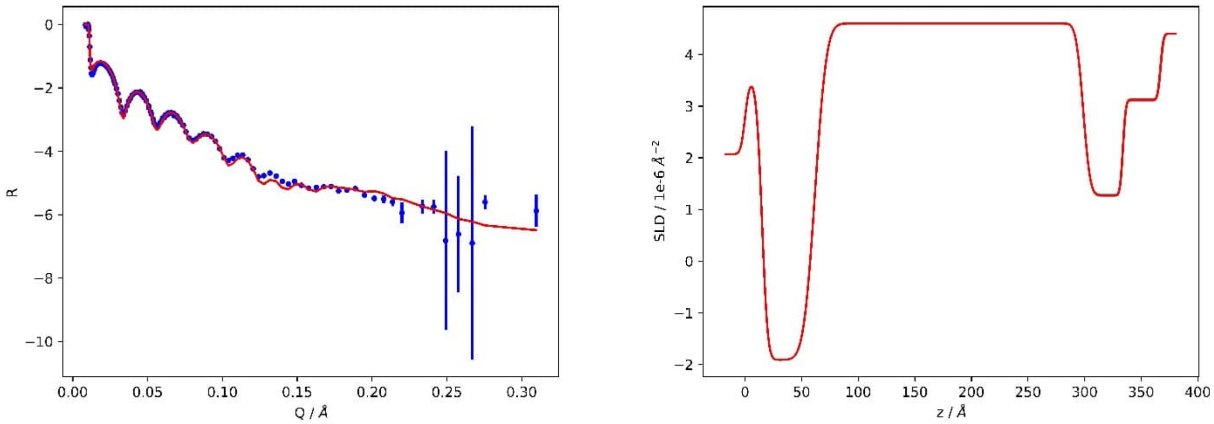

Fig. S99 PS and 20 cmc h-SDS

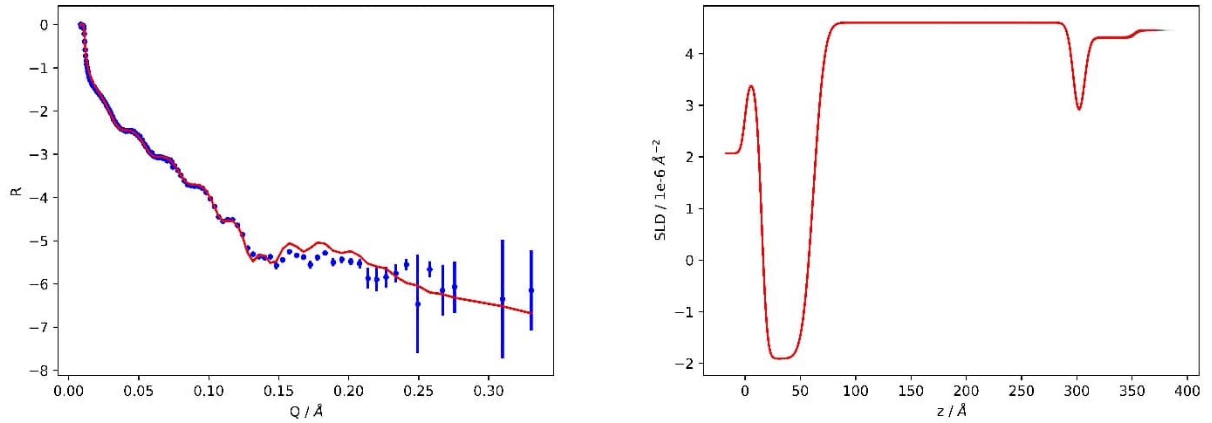

Fig. S100 PS after final rinse

Table S10 Bulk SLD values and fitted layer thickness, roughness and SLD values for adsorbed layers on PS surface (sequence NR2). Numbers in parenthesis indicate the error on the parameter value, equal to 2.5σ. The values in italics were manually adjusted, while *steps* in italics have a large associated error (poor contrast to bulk). Steps that are not shown fit the same model as PS in pure solvent

	bulk SLD ($*10^{-6}$Å^{-2})	SLD ($*10^{-6}$Å^{-2})	Thickness (Å)	Roughness (Å)
Thiol layer	*4.53*	1.16 (0.6)	6.02 (0.07)	4.7 (0.3)
20 cmc d42-CTAC	*4.53*	4.76 (0.01)	56 (2)	13 (2)
d42-CTAC/oligomer 1	*4.53*	4.668 (0.009)	69 (2)	3 (3)
Rinse	*4.53*	3.20 (0.02)	2.5 (0.2)	2.0 (0.1)
Chitosan oligomer	*4.5*	3.2	3	2
Rinse	*4.53*	3.2	3	2
Chitosan polymer	*4.53*	3.2	3	4
20 cmc d25-SDS	*4.45*	5.57 (0.01)	36.0 (0.4)	2.02 (0.07)
		4.78 (0.01)	41.9 (0.8)	2.1 (0.3)
Rinse	*4.53*	3.2	3	2
pDADMAC	*4.53*	0.5	0.5	4
Rinse	*4.45*	0.2	1	3
0.5 cmc h-SDS	*4.4*	1.10 (0.01)	36.3 (0.1)	2.01 (0.03)
		4.4	10.51 (0.08)	*2*
		0.28	19.4 (0.1)	2.01 (0.02)
20 cmc h-SDS	*4.4*	1.27 (0.02)	29.0 (0.3)	2.02 (0.06)
		3.12 (0.02)	33.2 (0.3)	2.03 (0.08)
Rinse	*4.45*	4.31 (0.01)	47 (2)	3 (3)

References

1 R. A. Campbell, *Current opinion in colloid & interface science*, 2018, 37, 49–60.

2 A. M. Poskanzer and F. Goodrich, *The Journal of Physical Chemistry*, 1975, 79, 2122–2126.

3 I. Umlong and K. Ismail, *Colloids and Surfaces A: Physicochemical and Engineering Aspects*, 2007, 299, 8–14.

4 N. Li, R. K. Thomas and A. R. Rennie, *Journal of Colloid and Interface Science*, 2012, 378, 152–158.

5 S. Cozzolino, P. Gutfreund, A. Vorobiev, R. J. L. Welbourn, A. Greaves, F. Zuttion, M. W. Rutland and G. S. Luengo, *submitted*, 2024.

6 S. Cozzolino, P. Gutfreund, A. Vorobiev, A. Devishvili, A. Greaves, A. Nelson, N. Yepuri, G. S. Luengo and M. W. Rutland, *Soft Matter*, 2024, 20, 7634–7645.

7 G. S. Luengo and A. J. Greaves, *Surface Science and Adhesion in Cosmetics*, 2021, 183–213.
